# Identification of *TMEM106B* as a Shared Potential Drug Target for Depression and Stroke Through Comprehensive Genetic Analyses

**DOI:** 10.1155/da/5250758

**Published:** 2025-09-05

**Authors:** Wenqiang Zhang, Lingli Qiu, Yunjie Liu, Yutong Wang, Mingshuang Tang, Lin Chen, Ben Zhang, Xia Jiang

**Affiliations:** ^1^Jiangxi Provincial Key Laboratory of Molecular Medicine, The Second Affiliated Hospital of Nanchang University, Nanchang, Jiangxi, China; ^2^Department of Medical Genetics, The Second Affiliated Hospital of Nanchang University, Nanchang, Jiangxi, China; ^3^Department of Epidemiology and Biostatistics, West China School of Public Health and West China Fourth Hospital, Sichuan University, Chengdu, Sichuan, China; ^4^Hainan General Hospital and Hainan Affiliated Hospital, Hainan Medical University, Haikou, Hainan, China;, West China School of Public Health and West China Fourth Hospital, Sichuan University, Chengdu, Sichuan, China; ^5^Department of Nutrition and Food Hygiene, West China School of Public Health and West China Fourth Hospital, Sichuan University, Chengdu, Sichuan, China; ^6^Department of Clinical Neuroscience, Karolinska Institutet, Stockholm, Sweden

**Keywords:** depression, genetic correlation, genome-wide cross-trait analysis, Mendelian randomization, stroke

## Abstract

**Background:** The coexistence of depression and stroke has long been observed; however, their intrinsic link has not been fully understood. We aimed to inform the importance of depression intervention as a primary prevention of stroke by investigating shared genetic etiology and causal relationship underlying depression and stroke.

**Methods:** Leveraging summary statistics from the hitherto largest genome-wide association studies (GWAS) in European-ancestry individuals for depression (*N*_case_/*N*_control_ = 294,322/741,438) and stroke (*N*_case_/*N*_control_ = 73,652/1,234,808), we performed cross-trait linkage-disequilibrium (LD) score regression and SUPERGNOVA to quantify global and local genetic correlations, cross-trait meta-analysis to identify shared genetic loci, transcriptome-wide association study (TWAS) to detect shared tissue-specific gene expression, and Mendelian randomization (MR) analysis to make causal inference between the two conditions.

**Results:** We observed a significant positive global genetic correlation between depression and stroke (rg = 0.18, *p*=2.92 ×10^−9^). Partitioning the whole genome, we observed one genomic region (11q23.2) presenting a significant local genetic correlation. Cross-trait meta-analysis and TWAS identified two shared genetic loci (*TMEM106B* and *FES*) revealing potential shared biological mechanisms involving lysosome localization. MR identified a putative causal association of genetically predicted depression on stroke (odds ratio [OR] = 1.13, 95% confidence interval [CI] = 1.07−1.19, *p*=1.12 ×10^−5^). A considerable proportion of this association was mediated through smoking initiation (proportion-mediated [PM] = 44.0%, 95% CI = 19.9%–68.1%, *p*=3.42 ×10^−4^), hypertension (PM = 34.0%, 95% CI = 14.5%–53.5%, *p*=6.46 ×10^−4^), type 2 diabetes (PM = 19.0%, 95% CI = 8.5%–29.5%, *p*=3.78 ×10^−4^), and atrial fibrillation (PM = 10.9%, 95% CI = 0.7%–21.1%, *p*=3.61 ×10^−2^), respectively. MR in the reverse direction identified a putative association of genetically predicted stroke on depression (OR = 1.05, 95% CI = 1.01−1.09, *p*=1.73 ×10^−2^), which attenuated to nonsignificant when correcting for both correlated and uncorrelated pleiotropy (OR = 1.00, 95% CI = 0.98−1.03, *p*=0.88). Drug target MR identified causal associations of genetically predicted *TMEM106B* level on depression (OR = 0.92, 95% CI = 0.90−0.94, *p*=2.04 ×10^−12^) and stroke (OR = 0.90, 95% CI = 0.86−0.95, *p*=3.53 ×10^−5^).

**Conclusion:** Our work highlights a shared genetic basis and a putative causal relationship between depression and stroke, providing novel insights into the primary prevention of stroke by depression intervention.

## 1. Introduction

Depression and stroke rank among the leading causes of global disability attributable to neuropsychiatric conditions, with depression accounting for the largest disability-adjusted life-years (DALYs) among mental disorders, and stroke for the largest DALYs among neurological conditions [1, 2]. The coexistence of these two common neuropsychiatric conditions has long been observed [3, 4]. Findings from a large-scale meta-analysis of cohort studies have suggested a bidirectional association between depression and stroke, manifested by a 41% significantly increased risk of stroke (hazard ratio [HR] = 1.41, 95% confidence interval [CI] = 1.32−1.50) for depression in 2,379,274 participants from 44 cohort studies [3], and a 162% significantly increased risk of depression (HR = 2.62, 95% CI = 2.09−3.29) for stroke in 93,076 participants from nine cohort studies [4]. In light of these observed phenotypic associations, the European Society of Cardiology and the American Heart Association have acknowledged screening and management of depression in patients with cardiovascular disease [5, 6]. Nevertheless, recommendations on depression intervention as a primary prevention of stroke remain sparse [5–7]. Indeed, current evidence supporting the causal role of depression in stroke remains inconclusive due to unmeasured confounding or reverse causation—inherent limitations of observational studies.

Phenotypic associations usually indicate shared genetic and/or environmental components. Investigating the genetic contributions to the phenotypic associations can help clarify the precise mechanisms underpinning the coexistence of complex diseases, which may aid in public health and clinical practice [8]. Leveraging summary statistics from genome-wide association studies (GWASs), a positive genetic correlation (rg = 0.07) between depression and stroke has been observed [9, 10], agreeing with the direction of phenotypic associations. Multiple genetic loci have been previously associated with both depression and stroke (e.g., *RPL31P12*, *BORCS7*) [9]. Mendelian randomization (MR) that uses genetic variants associated with exposure as instrumental variables (IVs) to estimate the putative causal effect of exposure on outcome, has identified a causal effect of depression on stroke (odds ratio [OR] = 1.21, 95% CI = 1.05−1.40 [9]; OR = 1.22, 95% CI = 1.09−1.38 [11]; OR = 1.12, 95% CI = 1.05−1.19 [12]), but no effect of stroke on depression (OR = 1.01, 95% CI = 0.97−1.06 [9]; OR = 1.01, 95% CI = 0.99−1.04 [11]).

Despite knowledge gained from postGWAS analyses advancing our understanding regarding genetic contributions to the association underlying depression and stroke, several gaps remain to be addressed. First, previous genetic studies have leveraged GWAS data with a small sample size and limited statistical power, preventing reliable characterization of the genetic association between depression and stroke [9–11, 13]. Second, previous MRs have shown directional inconsistency in the causal effects across varying methods [11, 13], and reported no effect of depression on ischemic stroke, the main etiological subtype of stroke [12, 14–16], preventing robust inference of the causal association between depression and stroke. Third, previous cross-phenotype association (CPASSOC) analysis has not considered heterogeneity across datasets for different phenotypes, preventing promising detection of shared associations between depression and stroke [9]. To our knowledge, no large-scale genetic analysis has been performed to comprehensively investigate the degree and nature of shared genetic etiology underlying depression and stroke, taking into account the etiological heterogeneity of stroke.

**Figure 1:**
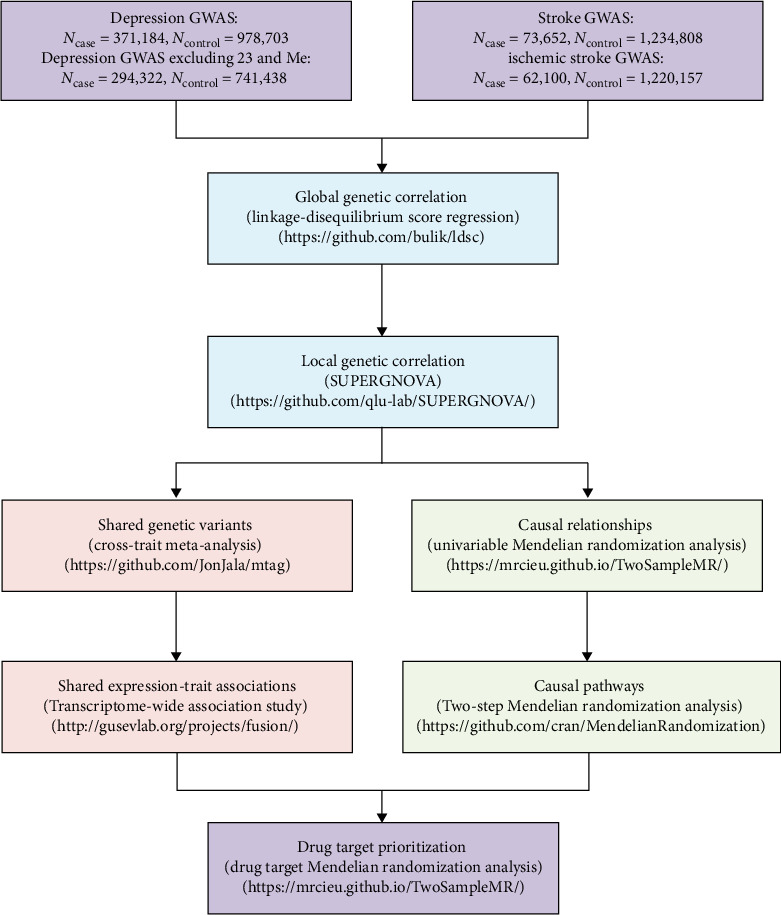
Flowchart of overall study design. GWAS, genome-wide association analysis.

Therefore, we performed a comprehensive genome-wide cross-trait analysis to investigate the shared genetic etiology and causal relationship underlying depression and stroke. Specifically, we used a variety of advanced analytical approaches, including genome-wide genetic correlation analysis to quantify global and local genetic overlap, cross-trait meta-analysis to identify shared genetic loci, transcriptome-wide association study (TWAS) to detect shared tissue-specific gene expressions, and MR analysis to infer causality between the two conditions. The overarching goal of our study was to reveal genetic underpinnings behind the observed phenotypic association, which may open new avenues for precision prevention and treatment for neuropsychiatric comorbidities. The overall study design is depicted in Figure 1.

## 2. Materials and Methods

### 2.1. Data Source

The hitherto largest GWAS of depression in European ancestry was conducted by meta-analyzing data from four study collections (iPSYCH2015, FinnGen, Million Veteran Program, meta-analysis of 23andMe, UK Biobank, and Psychiatric Genomics Consortium excluding iPSYCH2012) totaling 371,184 cases and 978,703 controls [17]. Depression was defined according to registered reports or self-reports. We obtained the full set GWAS summary statistics (294,322 cases and 741,438 controls, excluding 23andMe) for genome-wide cross-trait analysis.

The hitherto largest GWAS of stroke in European ancestry was conducted by the GIGASTROKE consortium, meta-analyzing data from 44 participating studies totaling 73,652 cases and 1,234,808 controls [18]. Stroke was defined according to the World Health Organization criteria or registered reports. Since stroke is predominantly caused by cerebral infarction, to increase the robustness of findings, we also included the most common etiological subtype, ischemic stroke. The hitherto largest GWAS of ischemic stroke in European ancestry was conducted by the GIGASTROKE consortium, meta-analyzing data from 38 participating studies totaling 62,100 cases and 1,220,157 controls [18]. We obtained the full set GWAS summary statistics for genome-wide cross-trait analysis.

### 2.2. Statistical Analysis

#### 2.2.1. Global and Local Genetic Correlation Analyses

We first evaluated global genetic correlation (rg) across the genome using cross-trait linkage-disequilibrium (LD) score regression to quantify the magnitude of shared genetic association between pairs of traits [19]. This algorithm requires only GWAS summary statistics, assuming that the effect for a given SNP aggregates the effects of all SNPs in LD with that SNP. A Bonferroni-corrected *p*-value (*p* < 0.025 = 0.05/2) was considered statistically significant.

We next evaluated local genetic correlation in 2353 predefined LD-independent regions using SUPERGNOVA to precisely quantify the shared genetic associations at these local genomic regions [20]. A Bonferroni-corrected *p*- value (*p* < 2.12 × 10^−5^ = 0.05/2353) was considered statistically significant.

#### 2.2.2. Cross-Trait Meta-Analysis

We performed a multitrait analysis of GWAS (MTAG) to identify shared genetic variants influencing pairs of traits [21]. MTAG combines GWAS summary statistics of multiple correlated traits to output trait-specific effect estimates for each SNP. To evaluate the robustness of MTAG results under modest genetic correlation, we calculated the upper bound of the false discovery rate (FDR) (maxFDR), which reflects the potential impact of violating the assumption that SNPs share a common variance–covariance matrix of effect sizes across traits. For each trait, independent lead SNPs were identified based on (1) reaching a genome-wide significance (*P*_MTAG_ < 5 × 10^−8^), (2) being 500 kb away from each other, and (3) being independent (*r*2 < 0.1). A lead SNP was considered to be novel if the SNP did not reach genome-wide significance in the original single-trait GWAS or the SNP was independent (*r*2 < 0.1) of those previously reported genome-wide significant SNPs for single traits. Shared genetic variants were defined if the lead SNPs associated with respective traits (e.g., depression and stroke) were in LD (*r*2 ≥ 0.8). We applied the Ensembl Variant Effect Predictor to annotate and map these loci by their physical position to genes [22]. Given that MTAG is unable to examine the association of one SNP with multiple traits, we further performed a CPASSOC through the statistic *S*_Het_ to evaluate the robustness of shared genetic variants identified by MTAG [23]. This algorithm combines GWAS summary statistics across correlated traits, while accounting for population structure and cryptic relatedness.

We next performed a colocalization analysis using Coloc to examine whether the shared loci colocalized at the same causal variant [24]. A locus was considered to be colocalized if the posterior probability of H4 ([PPH4], a shared causal variant) was greater than 0.7.

#### 2.2.3. TWAS Analysis

We performed a TWAS analysis using FUSION to identify shared independent genes whose expression pattern across tissues implicates shared etiology or biological mechanisms [25]. This algorithm combines GWAS summary statistics with precomputed gene expression weights to test the association of each gene to disease. We first performed single-trait TWAS by combining GWAS summary statistics with precomputed expression reference weights from 49 postmortem Genotype-Tissue Expression project (GTEx version 8) tissues to identify Bonferroni significant expression-trait associations (*p*_Bonferroni_ < 0.05) within each tissue. We next performed joint/conditional tests for loci with multiple associated features to identify independent genes at each locus. We further performed a colocalization analysis using Coloc to examine whether GWAS signals and GTEx expression quantitative trait loci signals colocalized at the same causal variant. We incorporated single-trait TWAS results to identify shared tissue–gene pairs across traits. To gain biological insights into the identified shared genes, we applied the WebGestalt (WEB-based Gene SeT AnaLysis Toolkit) tool to examine the enrichment of shared genes in Gene Ontology (GO) biological processes [26].

#### 2.2.4. Univariable MR Analysis

We performed a bidirectional two-sample MR analysis using TwoSampleMR to evaluate the potential causality [27]. For depression, a total of 251 independent depression-associated SNPs (*p* < 5 × 10^−8^, *r*2 < 0.1 within 3 Mb windows) were identified [17] and used as IVs (Supporting Information 1: Tables S1,2). For stroke phenotypes, a total of 23 independent stroke-associated SNPs (*p* < 5 × 10^−8^) and 27 independent ischemic stroke-associated SNPs were identified [18] and used as IVs (Supporting Information 1: Tables S3,4). We applied the inverse-variance weighted (IVW) method as our primary method to achieve the greatest statistical power. We performed sensitivity analyses using weighted-median [28], MR-Egger regression [29], and MR Pleiotropy RESidual Sum and Outlier (MR-PRESSO) [30] to evaluate the robustness of primary results. We repeated IVW excluding pleiotropic IVs (SNPs associated with potential confounders according to the GWAS Catalog) or palindromic IVs (A/T or G/C alleles). We further performed a causal analysis using summary effect (CAUSE) estimates as a complementary analysis while accounting for both correlated and uncorrelated pleiotropy, guaranteeing the “exclusion restriction” and “exchangeability” assumptions [31]. Compared to traditional MR methods, CAUSE further corrects correlated pleiotropy by constructing a Bayesian model that distinguishes whether the observed effect of a genetic variant on the outcome is truly mediated through the exposure of interest or instead reflects a shared confounding factor. A Bonferroni-corrected *p* value (*p* < 0.025 = 0.05/2) was considered statistically significant.

We estimated the phenotypic variance in the exposure trait explained by IVs [32] and calculated the *F*-statistic to evaluate the strength of IVs, validating the “relevance” assumption [33]. We calculated the statistical power of MR [34].

#### 2.2.5. MR Mediation Analysis

We performed a two-step MR analysis to evaluate the mediation effects of smoking [35], hypertension (ukb-b-14057) [36], type 2 diabetes [37], and atrial fibrillation [38], four potential important mediators of the association between depression and stroke [39–42]. We first performed an univariable MR analysis to estimate the effect of the exposure on the mediator. We next performed a multivariable MR analysis to estimate the independent effect of the mediator on the outcome after adjusting for the exposure. We applied the “product of coefficients” method to estimate the indirect effects [43]. The proportion-mediated (PM) was then derived by dividing the indirect effect by the total effect. CIs were estimated using the delta method.

#### 2.2.6. Drug Target MR Analysis

To investigate the therapeutic potential of the shared genes identified through prior analyses, we performed a drug target MR analysis to evaluate the effect of the encoded protein level on depression and stroke, along with potential mediators of the association between these two conditions. In this framework, genetic variants near or within druggable genes (often cis-acting variants) serve as proxies for therapeutic modulation. Using the lead cis-acting protein quantitative trait loci (*cis*-pQTL; the top variant within 300 kb of the protein-encoding gene) [44] as IV, we applied the Wald ratio method to estimate the drug target effect.

## 3. Results

### 3.1. Global and Local Genetic Correlation

**Figure 2:**
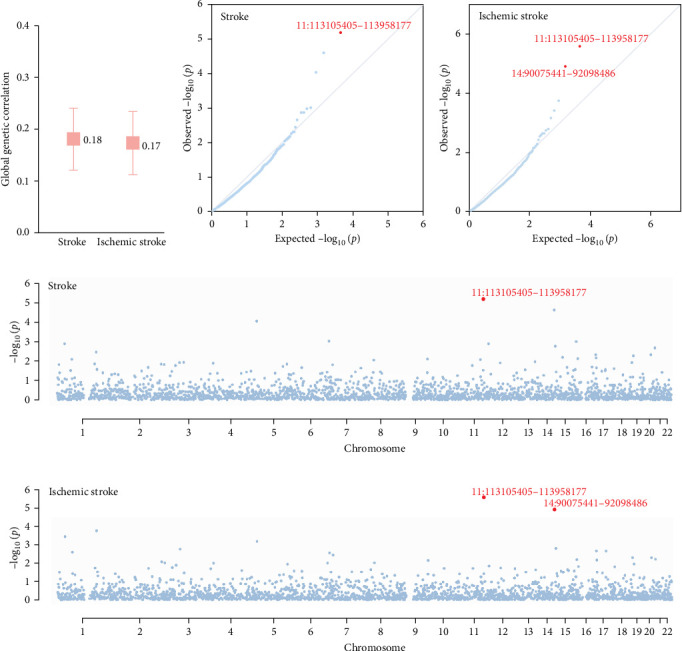
Genome-wide genetic correlation between depression and stroke. The boxes (A) denote the point estimate of the global genetic correlation, and the error bars denote 95% confidence intervals. In the QQ plots (B, C) and Manhattan plots (D, E), red points represent genomic regions that contribute significant local genetic correlation as estimated by SUPERGNOVA (*p* < 0.05/2353).

We observed a positive global genetic correlation between depression and stroke (rg = 0.18, *p*=2.92 × 10^−9^) (Figure 2A). A similar magnitude was observed for depression and ischemic stroke (rg = 0.17, *p*=2.48 × 10^−8^).

Partitioning the whole genome into 2353 LD-independent regions (Figure 2B–E and Supporting Information 1: Table S5), we identified one genomic region (11q23.2) presenting a significant local genetic correlation for depression and stroke. This genomic region that harbors multiple known depression-associated genes (e.g., *NCAM1*, *TTC12*, *DRD2*) [17] remained significant for depression and ischemic stroke. An additional genomic region (14q32.11-q32.12) showed a significant local signal specific to depression and ischemic stroke. The *RPS6KA5* locus on 14q32.11-q32.12 was associated with depression [17].

### 3.2. Cross-Trait Meta-Analysis

**Figure 3:**
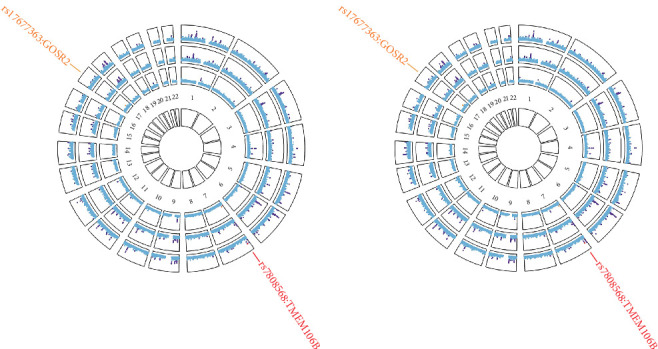
Shared genetic variants between depression and stroke. In the circular Manhattan plots (A, B), the outermost circle shows the Cross-Phenotype Association (CPASSOC) results between depression and stroke (or ischemic stroke); from the periphery to the center, each circle shows the multitrait analysis of GWAS (MTAG) results on depression and stroke (or ischemic stroke), respectively. The purple points represent genome-wide significant variants (*p* < 5 × 10^−8^) whereas the blue points represent non-genome-wide significant variants. The red points represent shared genetic variants between depression and stroke (or ischemic stroke) whereas the orange points represent novel genetic variants associated with stroke (or ischemic stroke).

MTAG analysis identified 194 independent lead SNPs for depression and 23 independent lead SNPs for stroke (Supporting Information 1: Tables S6–9), of which two loci (rs7808568 and rs17677363) have not to our knowledge been previously reported in GWASs of stroke (Figure 3A,B). Only the *TMEM106B* locus was shared between depression and stroke, although with different, yet highly correlated lead SNPs (rs6460906 for depression and rs7808568 for stroke, *r*2 = 0.96) (Supporting Information 1: Table S10). Of note, this locus colocalized at the same candidate causal variant (PPH4 = 0.86), which was replicated by CPASSOC (*p*_CPASSOC_ < 5 × 10^−8^) and also shared between depression and ischemic stroke. The maxFDR was 1.17 × 10^−4^ for depression, 0.06 for stroke, and 0.06 for ischemic stroke, suggesting that any bias due to MTAG assumption violations is likely negligible.

### 3.3. TWAS Analysis

TWAS analysis identified three significant tissue–gene pairs shared between depression and stroke, including *TMEM106B* in the transverse colon, *FES* in the heart atrial appendage, and *FES* in the stomach (Supporting Information 1: Figure S1A), which were also shared between depression and ischemic stroke (Supporting Information 1: Table S11). Of note, *TMEM106B* was also identified by our cross-trait meta-analysis.

GO analysis (Supporting Information 1: Figure S1B) across the two shared genes revealed a significant enrichment in the lysosome localization.

### 3.4. Univariable MR Analysis

**Figure 4:**
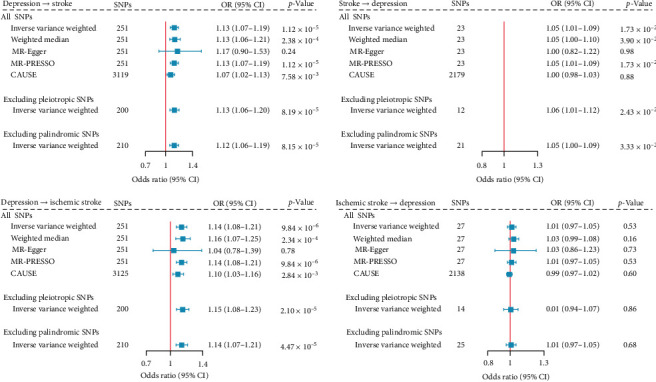
Bidirectional mendelian randomization analysis between depression and stroke. The boxes denote the point estimate of the causal effects (odds ratio [OR]), and the error bars denote 95% confidence intervals (95% CI).

Using IVW, MR analysis identified a putative causal effect of depression on stroke (OR = 1.13, 95% CI = 1.07−1.19, *p*=1.12 × 10^−5^). While the MR-Egger estimate did not reach statistical significance, the effect remained directionally consistent across the weighted-median, MR-Egger regression, and MR-PRESSO methods, and was not affected by both correlated and uncorrelated pleiotropy (CAUSE: OR = 1.07, 95% CI = 1.02−1.13, *p*=7.58 × 10^−3^) (Figure 4). When we limited the outcome to ischemic stroke, the effect remained consistent (IVW: OR = 1.14, 95% CI = 1.08−1.21, *p*=9.84 × 10^−6^), corroborating the robustness of the findings.

Conversely, using IVW, MR analysis also identified a putative causal effect of stroke on depression (OR = 1.05, 95% CI = 1.01−1.09, *p*=1.73 × 10^−2^), which attenuated to null when correcting for both correlated and uncorrelated pleiotropy (CAUSE: OR = 1.00, 95% CI = 0.98−1.03, *p*=0.88) or limiting the exposure to ischemic stroke (IVW: OR = 1.01, 95% CI = 0.97−1.05, *p*=0.53).

The mean *F*-statistics of our IVs ranged from 38.02 to 42.82 (Supporting Information 1: Table S12), indicating strong instruments. With the current sample size of outcome, assuming 0.79% (depression), 0.07% (stroke), and 0.08% (ischemic stroke) of the phenotypic variance explained by IVs, we had 80% power to detect an OR of 1.12 for depression on stroke, 1.13 for depression on ischemic stroke, 1.25 for stroke on depression, and 1.23 for ischemic stroke on depression, respectively.

### 3.5. MR Mediation Analysis

**Figure 5:**
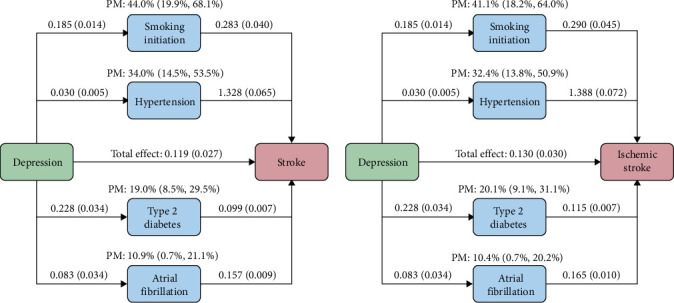
Mediation analysis between depression and stroke. The effect estimates (beta coefficients) are provided with standard errors. The proportions-mediated (PM) are provided with 95% confidence intervals.

Of the total effect of depression with stroke, the PM was estimated as 44.0% (95% CI = 19.9%–68.1%, *p*=3.42 × 10^−4^) through smoking initiation, 34.0% (95% CI = 14.5%–53.5%, *p*=6.46 × 10^−4^) through hypertension, 19.0% (95% CI = 8.5%–29.5%, *p*=3.78 × 10^−4^) through type 2 diabetes, and 10.9% (95% CI = 0.7%–21.1%, *p*=3.61 × 10^−2^) through atrial fibrillation (Figure 5). Similar patterns were observed for the association of depression with ischemic stroke, 41.1% (95% CI = 18.2%−64.0%, *p*=4.31 × 10^−4^) mediated through smoking initiation, 32.4% (95% CI = 13.8%−50.9%, *p*=6.29 × 10^−4^) through hypertension, 20.1% (95% CI = 9.1%−31.1%, *p*=3.40 × 10^−4^) through type 2 diabetes, and 10.4% (95% CI = 0.7%–20.2%, *p*=3.60 × 10^−2^) through atrial fibrillation. After adjusting for each mediator individually in multivariable MR, the direct effect of depression on stroke was attenuated and no longer statistically significant (Supporting Information 1: Table S13), suggesting that these risk factors may partially mediate the observed association.

### 3.6. Drug Target MR Analysis

**Figure 6:**
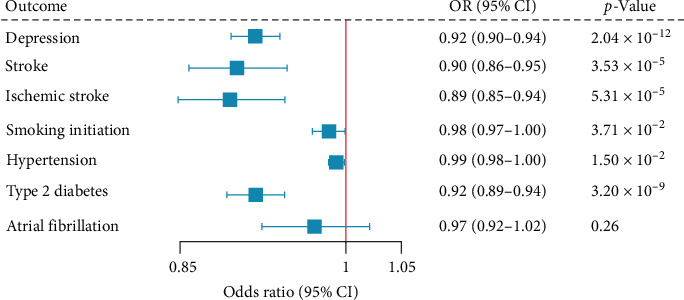
Drug target Mendelian randomization analysis between serum TMEM106B levels, depression, and stroke. The boxes denote the point estimate of the causal effects (odds ratio [OR]), and the error bars denote 95% confidence intervals (95% CI).

Drug target MR (Figure 6) identified causal associations of serum *TMEM106B* level on depression (OR = 0.92, 95% CI = 0.90−0.94, *p*=2.04 × 10^−12^), stroke (OR = 0.90, 95% CI = 0.86−0.95, *p*=3.53 × 10^−5^), ischemic stroke (OR = 0.89, 95% CI = 0.85−0.94, *p*=5.31 × 10^−5^), smoking initiation (OR = 0.98, 95% CI = 0.97−1.00, *p*=3.71 × 10^−2^), hypertension (OR = 0.99, 95% CI = 0.98−1.00, *p*=1.50 × 10^−2^), and type 2 diabetes (OR = 0.92, 95% CI = 0.89−0.94, *p*=3.20 × 10^−9^). Genetically predicted *TMEM106B* level was not associated with atrial fibrillation (OR = 0.97, 95% CI = 0.92−1.02, *p*=0.26).

## 4. Discussion

This is a comprehensive large-scale genome-wide cross-trait analysis to investigate the genetic correlation, shared genetic loci, shared tissue-specific gene expressions, and causal relationship between depression and stroke. We found a significant genetic correlation at global and local genomic levels, indicating a shared genetic basis between depression and stroke. Such genetic overlap was further substantiated by one shared independent locus, three shared expression-trait associations, and a putative causal relationship. A considerable proportion of this causal relationship was mediated by smoking initiation, hypertension, type 2 diabetes, and atrial fibrillation. These findings advance our understanding of the complicated relationship underlying these two common neuropsychiatric disorders, and provide important implications for their prevention and treatment.

Our findings are primarily in line with those from existing studies, yet remarkably expand previous work in several critical aspects. First, depression and stroke share a genetic basis at global and local genomic levels. The positive significant global genetic correlation (rg = 0.18, *p*=2.92 × 10^−9^) observed by us showed a more pronounced magnitude and significance than that of two existing analysis reporting an estimate of 0.07 (*p*=0.12; *p*=0.10) [9, 10]. Different from and extending those findings, we further partitioned the whole genome into LD-independent regions and identified one significant local signal, corroborating the genome-wide genetic correlations. Second, genetic liability to depression appears to increase the risk of stroke. Our MR analyses provided consistent evidence supporting a causal role of depression in stroke and substantially improved statistical power compared to prior MR analyses. This was achieved by leveraging the most up-to-date GWAS data on depression and stroke in individuals of European ancestry and by incorporating 251 independent SNPs as IVs—considerably more than those used in prior studies (46 [9], 83 [11],137 [12], and 49 [13])—thereby increasing the proportion of variance explained and the robustness of the causal inference. When restricting the outcome to ischemic stroke, we found a consistent causal association with even more pronounced magnitude and significance, further corroborating the reliability of the findings [14–16]. Our MR mediation analysis further uncovered the role of smoking initiation, hypertension, type 2 diabetes, and atrial fibrillation in mediating the causal effect of depression on stroke, which promotes our understanding of causal pathways underlying depression and cardiovascular diseases [9–16]. Furthermore, our reverse MR analyses detected neither a causal effect of stroke nor ischemic stroke on depression [9, 11, 14, 15], suggesting that the observed phenotypic association may be driven by residual confounding or reverse causation [4]. Collectively, our MR findings support depression as a risk factor rather than as an adverse clinical outcome in the pathogenesis of stroke.

In addition to a significant genetic correlation and causal relationship identified by us, results from cross-trait meta-analysis and TWAS suggest that the observed phenotypic association between depression and stroke can be somewhat explained by shared genetic loci. Specifically, the *TMEM106B* locus was not only identified by both cross-trait meta-analysis at the variant-based association and TWAS at the gene-based association to be shared between depression and stroke, but MR pinpointed its encoding protein as a shared drug target. *TMEM106B* encodes a transmembrane protein 106B located on late endosome and lysosome, involved in dendrite morphogenesis and lysosome localization. Deficiency of *TMEM106B* triggers abnormal lysosomal morphology and function, inducing hypomyelination in oligodendrocytes, further activating chronic neuroinflammation, and disrupting signal transduction, thereby driving the neuropsychiatric lesions [45]. Previous protein-wide and transcriptome-wide MR have consistently identified *TMEM106B* as a candidate causal gene for depression [46], and our results further identify its role in the development of stroke consolidating findings from a previous cross-trait meta-analysis [9]. Despite the absence of evidence for drug development specifically targeting *TMEM106B* in depression or stroke, this gene nonetheless emerges as a prioritized and promising shared drug target for the two conditions. Additionally, the *FES* locus was identified by TWAS to be shared between depression and stroke. *FES* encodes a nonreceptor protein-tyrosine kinase, involved in maintaining cellular transformation. Deficiency of *FES* in mice increases atherosclerotic plaque sizes and within-plaque abundance of monocytes/macrophages and smooth muscle cells, both of which are well-established players in atherogenesis as an underlying mechanism of stroke [47, 48]. Previous TWAS has identified *FES* to be shared between carotid intima–media thickness and stroke [49], and our results further identify its role in the development of depression. Of note, these two shared loci were identified to be significantly enriched in the biological process of lysosome localization (GO:0032418), driving novel biological insights into the shared genetic susceptibility to depression and stroke. Future functional exploration is warranted to reveal the potential pathophysiological mechanism.

Our findings provide translational implications for public health and clinical practice. First, genetic liability to depression may increase the risk of stroke. Given that depression is potentially treatable, this highlights the clinical relevance of integrating effective psychological interventions and mental healthcare into stroke prevention strategies. Such an approach may help reduce stroke risk in individuals with depression and address existing evidence gaps regarding the prevention of cardiovascular disease in populations with mental disorders, as acknowledged by the European Society of Cardiology guidelines [6]. Second, depression and stroke are inherently linked through shared genetic components. The identification of *TMEM106B* highlights a biologically plausible candidate gene that may contribute to the underlying mechanisms connecting these conditions. While not yet a validated therapeutic target, *TMEM106B* warrants further investigation to explore its potential role in the pathophysiology of neuropsychiatric comorbidities. Future large-scale GWAS efforts may help clarify shared genetic pathways and inform the development of novel therapeutics or repurposing strategies.

We acknowledge several potential limitations. First, our primary findings were restricted to participants of European ancestry, which may not be generalizable to other ethnic populations. Second, UK Biobank and FinnGen study participants were included in both depression GWAS and stroke GWAS, resulting in an estimated 41.7% sample overlap. However, the lower limit of the one-sided 95% CI for the *F*-statistic remained high (41.47 for depression IVs, 35.62 for stroke IVs, and 34.20 for ischemic stroke IVs), thus considerable bias due to participant overlap in our two-sample MR was not expected [50]. Moreover, we applied CAUSE, a robust method that explicitly accounts for sample overlap as well as correlated and uncorrelated pleiotropy [31]. The consistency of CAUSE results with our primary IVW estimates further supports the validity of our findings. Additionally, other genome-wide cross-trait analysis methods we have used are robust to sample overlap [19–21]. Third, we observed little evidence of shared genetic loci between depression and stroke, which may result from pathogenetic heterogeneity in stroke diagnoses. However, the replication using data from the largest European GWAS of ischemic stroke indicates the robustness of the findings. Future GWAS of stroke with larger sample sizes is still needed to achieve a more accurate picture of the shared genetic etiology between depression and stroke.

## 5. Conclusions

To conclude, leveraging the largest GWAS data of European ancestry with cutting-edge postGWAS analytical methods, our work confirms a putative causal effect of depression on stroke acting partially through smoking initiation, hypertension, type 2 diabetes, and atrial fibrillation, and reveals *TMEM106B* as a shared potential drug target for depression and stroke. Our findings clarify the shared genetic etiology and causal relationship underlying the observed phenotypic link between depression and stroke, and provide novel insight into precision prevention and medicine for common neuropsychiatric comorbidities.

## Data Availability

The full GWAS summary statistics for depression, stroke, and ischemic stroke are available on https://ipsych.dk/en/research/downloads/, https://www.ebi.ac.uk/gwas/studies/GCST90104539, and https://www.ebi.ac.uk/gwas/studies/GCST90104540, respectively.

## Supporting Information

Supporting Information 1 Figure S1. Shared TWAS significant genes and biological processes between carotid intima–media thickness and stroke. Supporting Information 1: Table S1. Characteristics of genetic instruments of depression and their effect sizes with stroke. Supporting Information 1: Table S2. Characteristics of genetic instruments of depression and their effect sizes with ischemic stroke. Supporting Information 1: Table S3. Characteristics of genetic instruments of stroke and their effect sizes with depression. Supporting Information 1: Table S4. Characteristics of genetic instruments of ischemic stroke and their effect sizes with depression. Supporting Information 1: Table S5. Local heritability of depression and stroke, and regions with significant local genetic covariance estimated by SUPERGNOVA. Supporting Information 1: Table S6. Lead SNPs for depression from MTAG analysis between depression and stroke. Supporting Information 1: Table S7. Lead SNPs for stroke from MTAG analysis between depression and stroke. Supporting Information Table S8. Lead SNPs for depression from MTAG analysis between depression and ischemic stroke. Supporting Information 1: Table S9. Lead SNPs for ischemic stroke from MTAG analysis between depression and ischemic stroke. Supporting Information 1: Table S10. Shared genetic variants identified by MTAG analysis between depression and stroke. Supporting Information 1: Table S11. Shared TWAS significant genes between depression and stroke across 49 GTEx Tissues (version 8). Supporting Information 1: Table S12. Data sources, sample sizes, number of instruments, and *F*-statistics. Supporting Information 1: Table S13. Mendelian randomization mediation analysis between depression and stroke.

Supporting Information 2 Reporting checklist for genetic association study.
