# Supplementary material for: Identification of TMEM106B as a Shared Potential Drug Target for Depression and Stroke Through Comprehensive Genetic Analyses
Source: Depress Anxiety. 2025 Sep 5;2025:5250758. doi: 10.1155/da/5250758 (PMC12431815; doi:10.1155/da/5250758)
Supplement: Supporting Information 1 — Figure S1. Shared TWAS significant genes and biological processes between carotid intima–media thickness and stroke. Supporting Information 1: Table S1. Characteristics of genetic instruments of depression and their effect sizes with stroke. Supporting Information 1: Table S2. Characteristics of genetic instruments of depression and their effect sizes with ischemic stroke. Supporting Information 1: Table S3. Characteristics of genetic instruments of stroke and their effect sizes with depression. Supporting Information 1: Table S4. Characteristics of genetic instruments of ischemic stroke and their effect sizes with depression. Supporting Information 1: Table S5. Local heritability of depression and stroke, and regions with significant local genetic covariance estimated by SUPERGNOVA. Supporting Information 1: Table S6. Lead SNPs for depression from MTAG analysis between depression and stroke. Supporting Information 1: Table S7. Lead SNPs for stroke from MTAG analysis between depression and stroke. Supporting Information Table S8. Lead SNPs for depression from MTAG analysis between depression and ischemic stroke. Supporting Information 1: Table S9. Lead SNPs for ischemic stroke from MTAG analysis between depression and ischemic stroke. Supporting Information 1: Table S10. Shared genetic variants identified by MTAG analysis between depression and stroke. Supporting Information 1: Table S11. Shared TWAS significant genes between depression and stroke across 49 GTEx Tissues (version 8). Supporting Information 1: Table S12. Data sources, sample sizes, number of instruments, and F-statistics. Supporting Information 1: Table S13. Mendelian randomization mediation analysis between depression and stroke. [file 5250758.f1.docx]

**Supplementary materials**

**Supplementary Figure 1. Shared TWAS significant genes and biological processes between carotid intima-media thickness and stroke.**

**Supplementary Table 1. Characteristics of genetic instruments of depression and their effect sizes with stroke.**

**Supplementary Table 2. Characteristics of genetic instruments of depression and their effect sizes with ischemic stroke.**

**Supplementary Table 3. Characteristics of genetic instruments of stroke and their effect sizes with depression.**

**Supplementary Table 4. Characteristics of genetic instruments of ischemic stroke and their effect sizes with depression.**

**Supplementary Table 5. Local heritability of depression and stroke, and regions with significant local genetic covariance estimated by SUPERGNOVA.**

**Supplementary Table 6. Lead SNPs for depression from MTAG analysis between depression and stroke.**

**Supplementary Table 7. Lead SNPs for stroke from MTAG analysis between depression and stroke.**

**Supplementary Table 8. Lead SNPs for depression from MTAG analysis between depression and ischemic stroke.**

**Supplementary Table 9. Lead SNPs for ischemic stroke from MTAG analysis between depression and ischemic stroke.**

**Supplementary Table 10. Shared genetic variants identified by MTAG analysis between depression and stroke.**

**Supplementary Table 11. Shared TWAS significant genes between depression and stroke across 49 GTEx Tissues (version 8).**

**Supplementary Table 12. Data sources, sample sizes, number of instruments and F-statistics.**

**Supplementary Table 13. Mendelian randomization mediation analysis between depression and stroke.
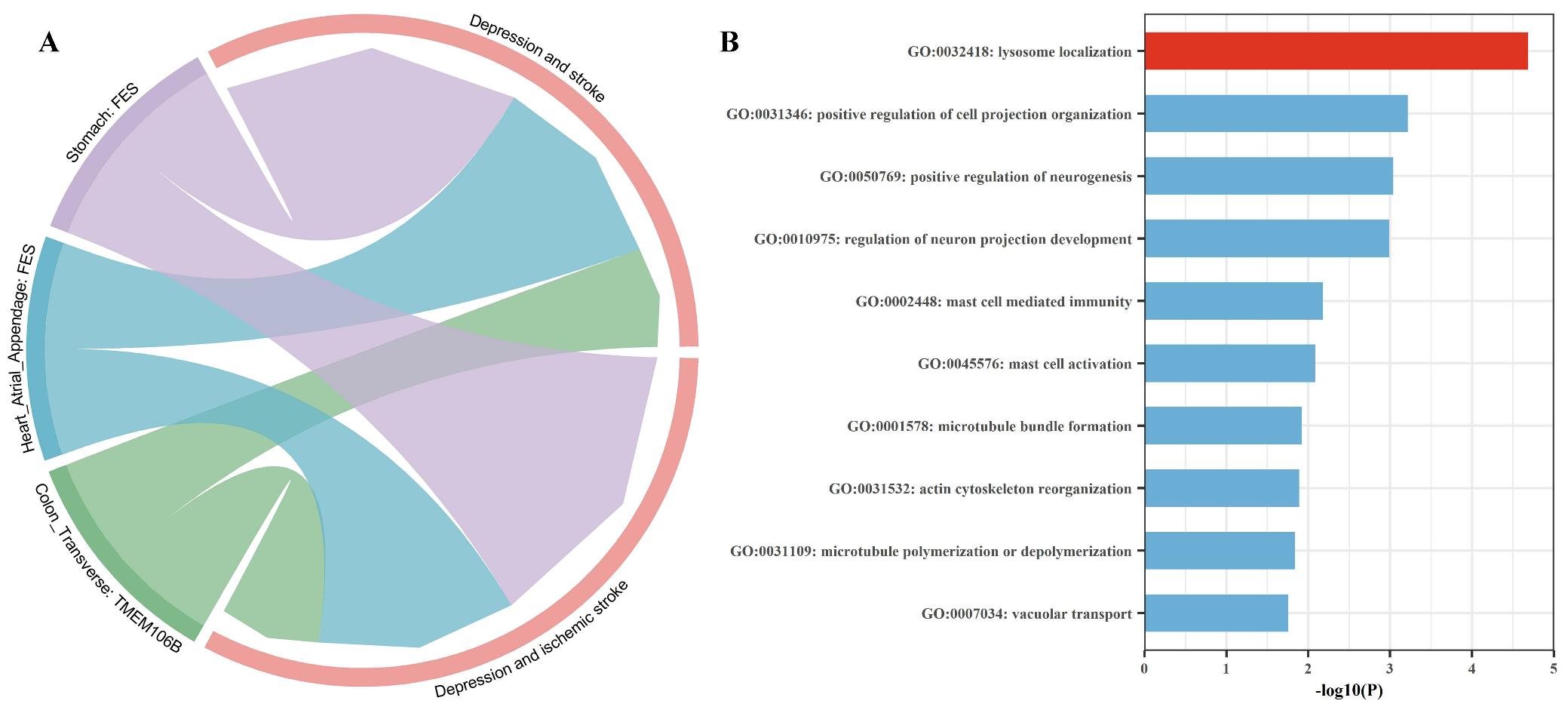
**

**Supplementary Figure 1. Shared TWAS significant genes and biological processes between carotid intima-media thickness and stroke.** The chord diagram **(A)** shows shared significant tissue-gene pairs for depression and stroke. The bar graph **(B)** shows the enrichment of shared genes in Gene Ontology biological processes. The red bar represents significant enrichment after controling the false discovery rate (FDR) (*P*_FDR_ < 0.05) whereas the blue bars represent suggestive enrichment (*P*_FDR_ > 0.05).

| **Supplementary Table 1. Characteristics of genetic instruments of depression and their effect sizes with stroke.** | | | | | | | | | | | | |
| --- | --- | --- | --- | --- | --- | --- | --- | --- | --- | --- | --- | --- |
| SNP | CHR | BP | A1 | A2 | EAF | Exposure | | | Outcome | | | Pleiotropic traits |
|  |  |  |  |  |  | Beta | SE | P | Beta | SE | P |  |
| rs301817 | 1 | 8503379 | C | A | 0.408 | 0.024 | 0.003 | 3.53E-13 | -0.021 | 0.007 | 0.002 |  |
| rs2089358 | 1 | 37194103 | C | T | 0.305 | 0.025 | 0.003 | 2.65E-15 | 0.005 | 0.008 | 0.556 | Insomnia |
| rs1466887 | 1 | 37709328 | C | T | 0.441 | 0.016 | 0.003 | 1.06E-08 | 0.008 | 0.006 | 0.209 |  |
| rs12141363 | 1 | 47696581 | C | A | 0.399 | 0.016 | 0.003 | 1.91E-08 | 0.017 | 0.007 | 0.010 |  |
| rs11579246 | 1 | 50559162 | A | G | 0.894 | 0.028 | 0.005 | 3.71E-09 | 0.012 | 0.010 | 0.254 |  |
| rs1890946 | 1 | 52342427 | T | C | 0.469 | -0.019 | 0.003 | 4.81E-12 | 0.000 | 0.006 | 0.982 |  |
| rs7531118 | 1 | 72837239 | T | C | 0.464 | -0.029 | 0.003 | 4.90E-24 | 0.009 | 0.007 | 0.207 | Weight, Waist circumference, Obesity, Intelligence, Body mass index, Hip circumference |
| rs4571923 | 1 | 73736562 | G | A | 0.513 | -0.023 | 0.003 | 9.77E-17 | -0.001 | 0.006 | 0.839 |  |
| rs10874020 | 1 | 79593241 | T | C | 0.300 | -0.017 | 0.003 | 4.90E-08 | 0.009 | 0.008 | 0.252 |  |
| rs2389015 | 1 | 80799190 | G | A | 0.731 | -0.022 | 0.003 | 3.16E-12 | -0.012 | 0.008 | 0.124 |  |
| rs7513962 | 1 | 96952532 | C | T | 0.480 | -0.018 | 0.003 | 4.30E-09 | -0.011 | 0.006 | 0.074 |  |
| rs16846561 | 1 | 173869111 | C | G | 0.896 | 0.029 | 0.005 | 2.15E-10 | -0.014 | 0.011 | 0.188 |  |
| rs10913112 | 1 | 175913828 | C | T | 0.628 | 0.022 | 0.003 | 7.69E-13 | 0.004 | 0.007 | 0.572 |  |
| rs72724899 | 1 | 177408112 | C | T | 0.924 | -0.034 | 0.005 | 6.71E-12 | 0.016 | 0.013 | 0.208 |  |
| rs74934992 | 1 | 191255088 | G | T | 0.956 | -0.043 | 0.007 | 3.05E-09 | -0.012 | 0.016 | 0.463 |  |
| rs2358493 | 1 | 197816439 | G | A | 0.491 | -0.023 | 0.003 | 8.97E-17 | 0.006 | 0.006 | 0.324 |  |
| rs57691884 | 1 | 198234477 | G | A | 0.924 | -0.030 | 0.005 | 1.35E-08 | -0.008 | 0.012 | 0.521 |  |
| rs12131852 | 1 | 214433323 | C | G | 0.712 | 0.017 | 0.003 | 2.52E-08 | -0.005 | 0.008 | 0.521 |  |
| rs2576241 | 1 | 217100192 | C | A | 0.461 | 0.017 | 0.003 | 5.45E-10 | -0.007 | 0.007 | 0.284 |  |
| rs4846898 | 1 | 230252218 | A | G | 0.590 | -0.017 | 0.003 | 8.16E-10 | 0.000 | 0.007 | 0.952 |  |
| rs12619197 | 2 | 22566548 | G | A | 0.540 | -0.021 | 0.003 | 2.14E-13 | -0.003 | 0.006 | 0.681 |  |
| rs4666020 | 2 | 28046028 | G | A | 0.799 | 0.019 | 0.004 | 4.39E-08 | 0.004 | 0.008 | 0.607 |  |
| rs13031157 | 2 | 51216810 | T | C | 0.571 | 0.019 | 0.003 | 1.40E-11 | 0.012 | 0.006 | 0.057 |  |
| rs7599039 | 2 | 51578355 | T | C | 0.790 | 0.020 | 0.003 | 6.22E-09 | 0.006 | 0.008 | 0.492 |  |
| rs4971586 | 2 | 52315585 | T | A | 0.523 | 0.019 | 0.003 | 3.81E-11 | 0.000 | 0.006 | 0.960 |  |
| rs11682175 | 2 | 57987593 | T | C | 0.540 | -0.021 | 0.003 | 2.85E-14 | -0.006 | 0.006 | 0.372 | Sleep duration, Schizophrenia, Autism spectrum disorder or schizophrenia, Irritable mood |
| rs34829058 | 2 | 60182065 | T | C | 0.754 | -0.018 | 0.003 | 1.68E-08 | -0.003 | 0.007 | 0.711 |  |
| rs4671458 | 2 | 63374063 | C | T | 0.784 | 0.020 | 0.004 | 1.16E-08 | -0.001 | 0.008 | 0.860 | Subjective well-being |
| rs10173072 | 2 | 71660804 | C | T | 0.566 | 0.016 | 0.003 | 1.09E-08 | -0.010 | 0.006 | 0.133 |  |
| rs7585722 | 2 | 86819128 | T | C | 0.857 | -0.022 | 0.004 | 2.29E-08 | 0.010 | 0.009 | 0.293 |  |
| rs2570485 | 2 | 104464467 | C | T | 0.753 | 0.022 | 0.003 | 1.72E-11 | 0.007 | 0.008 | 0.398 |  |
| rs62170849 | 2 | 125012926 | T | C | 0.710 | 0.020 | 0.003 | 1.31E-10 | 0.004 | 0.007 | 0.601 |  |
| rs13020607 | 2 | 127163515 | T | C | 0.843 | 0.022 | 0.004 | 1.20E-08 | 0.009 | 0.009 | 0.326 |  |
| rs12477455 | 2 | 144079060 | C | T | 0.547 | 0.017 | 0.003 | 6.35E-09 | -0.001 | 0.007 | 0.847 |  |
| rs1320138 | 2 | 144158287 | T | C | 0.433 | 0.019 | 0.003 | 7.50E-12 | 0.007 | 0.007 | 0.282 |  |
| rs835302 | 2 | 157123393 | G | T | 0.215 | -0.024 | 0.003 | 1.28E-12 | 0.004 | 0.008 | 0.646 |  |
| rs10205665 | 2 | 161480003 | G | A | 0.669 | -0.017 | 0.003 | 2.55E-08 | -0.007 | 0.007 | 0.292 |  |
| rs1267079 | 2 | 162033854 | T | C | 0.695 | 0.022 | 0.003 | 2.47E-13 | 0.004 | 0.007 | 0.583 |  |
| rs908670 | 2 | 172690180 | T | C | 0.729 | -0.017 | 0.003 | 2.49E-08 | 0.012 | 0.007 | 0.104 |  |
| rs4972656 | 2 | 175240292 | C | T | 0.425 | -0.021 | 0.003 | 2.12E-13 | -0.004 | 0.007 | 0.562 |  |
| rs6713469 | 2 | 195882599 | A | G | 0.832 | 0.021 | 0.004 | 1.93E-08 | 0.005 | 0.009 | 0.561 |  |
| rs6715105 | 2 | 198445601 | T | C | 0.337 | 0.019 | 0.003 | 4.31E-10 | 0.011 | 0.007 | 0.129 |  |
| rs55772859 | 2 | 208042581 | C | A | 0.677 | -0.022 | 0.003 | 2.46E-13 | 0.006 | 0.007 | 0.361 | Insomnia |
| rs7576085 | 2 | 210187488 | C | T | 0.451 | -0.016 | 0.003 | 1.42E-08 | 0.005 | 0.006 | 0.446 |  |
| rs1801251 | 2 | 233633460 | G | A | 0.669 | -0.018 | 0.003 | 5.79E-10 | -0.015 | 0.007 | 0.032 | Coronary artery disease |
| rs4663414 | 2 | 235352188 | T | G | 0.378 | 0.016 | 0.003 | 1.60E-08 | 0.005 | 0.007 | 0.452 |  |
| rs6443178 | 3 | 8278215 | C | T | 0.462 | 0.016 | 0.003 | 4.57E-08 | -0.008 | 0.007 | 0.274 |  |
| rs6800583 | 3 | 16851755 | G | A | 0.632 | -0.016 | 0.003 | 1.41E-08 | 0.001 | 0.007 | 0.946 |  |
| rs77463213 | 3 | 43432024 | C | T | 0.849 | -0.022 | 0.004 | 1.16E-08 | -0.014 | 0.009 | 0.116 |  |
| rs13058913 | 3 | 44732013 | A | T | 0.682 | -0.025 | 0.003 | 2.46E-16 | -0.007 | 0.007 | 0.321 |  |
| rs7636868 | 3 | 45373208 | G | A | 0.291 | 0.018 | 0.003 | 4.26E-09 | -0.005 | 0.008 | 0.545 |  |
| rs7617480 | 3 | 49210732 | A | C | 0.216 | 0.032 | 0.003 | 1.21E-20 | -0.004 | 0.008 | 0.681 | Smoking cessation, Subjective well-being, Menarche (age at onset) |
| rs1010553 | 3 | 52540773 | T | C | 0.523 | -0.019 | 0.003 | 1.63E-11 | -0.004 | 0.007 | 0.526 | Hematocrit |
| rs17216573 | 3 | 56282978 | T | C | 0.853 | 0.022 | 0.004 | 4.16E-08 | 0.016 | 0.009 | 0.088 | Lung function (FEV1/FVC) |
| rs1470035 | 3 | 61184321 | G | A | 0.591 | -0.017 | 0.003 | 2.09E-09 | -0.002 | 0.006 | 0.738 |  |
| rs116310555 | 3 | 71814431 | T | C | 0.882 | -0.025 | 0.004 | 8.96E-09 | -0.024 | 0.010 | 0.014 |  |
| rs1597076 | 3 | 108484536 | G | A | 0.673 | -0.017 | 0.003 | 2.34E-08 | -0.008 | 0.007 | 0.263 |  |
| rs6783233 | 3 | 117509984 | C | T | 0.731 | -0.019 | 0.003 | 6.38E-10 | 0.002 | 0.007 | 0.832 |  |
| rs7618264 | 3 | 132680873 | A | T | 0.453 | 0.016 | 0.003 | 8.93E-09 | -0.007 | 0.007 | 0.255 |  |
| rs827137 | 3 | 158008124 | C | T | 0.559 | -0.026 | 0.003 | 3.48E-16 | 0.012 | 0.007 | 0.085 |  |
| rs536445 | 3 | 173120103 | C | T | 0.475 | -0.019 | 0.003 | 1.49E-11 | -0.020 | 0.007 | 0.003 |  |
| rs2298969 | 4 | 3186244 | A | G | 0.483 | 0.017 | 0.003 | 3.05E-09 | 0.003 | 0.007 | 0.616 |  |
| rs9306914 | 4 | 34552161 | A | T | 0.838 | -0.025 | 0.005 | 2.77E-08 | -0.009 | 0.009 | 0.309 |  |
| rs114851235 | 4 | 59833409 | A | G | 0.946 | -0.043 | 0.007 | 9.34E-11 | -0.010 | 0.014 | 0.458 |  |
| rs34207047 | 4 | 80199323 | C | A | 0.478 | 0.021 | 0.003 | 1.42E-13 | -0.002 | 0.007 | 0.807 |  |
| rs6813752 | 4 | 91099481 | T | A | 0.373 | -0.018 | 0.003 | 1.05E-09 | 0.000 | 0.007 | 0.972 |  |
| rs6845263 | 4 | 102276682 | T | C | 0.500 | 0.015 | 0.003 | 3.79E-08 | 0.011 | 0.007 | 0.114 | Superior crus of antihelix expression |
| rs45510091 | 4 | 123186393 | A | G | 0.949 | 0.045 | 0.007 | 4.03E-12 | 0.021 | 0.015 | 0.152 | Feeling miserable |
| rs35553410 | 4 | 131237381 | T | C | 0.748 | -0.021 | 0.004 | 1.52E-09 | -0.008 | 0.008 | 0.297 |  |
| rs6536630 | 4 | 140919035 | T | A | 0.465 | 0.018 | 0.003 | 1.12E-09 | 0.014 | 0.007 | 0.034 |  |
| rs17611770 | 4 | 176851045 | A | T | 0.799 | -0.020 | 0.004 | 1.78E-08 | -0.013 | 0.008 | 0.119 | Neuroticism |
| rs7659414 | 4 | 177350956 | A | C | 0.591 | -0.017 | 0.003 | 8.54E-09 | -0.003 | 0.007 | 0.675 |  |
| rs2220599 | 5 | 7378854 | C | G | 0.649 | -0.019 | 0.003 | 2.40E-10 | 0.000 | 0.007 | 0.999 | Subjective well-being |
| rs7700453 | 5 | 20126890 | G | C | 0.472 | 0.016 | 0.003 | 3.09E-08 | 0.016 | 0.007 | 0.019 |  |
| rs10805794 | 5 | 27024536 | C | A | 0.487 | 0.017 | 0.003 | 2.55E-09 | -0.007 | 0.006 | 0.303 |  |
| rs12517438 | 5 | 30842054 | T | G | 0.469 | -0.019 | 0.003 | 3.05E-11 | 0.004 | 0.006 | 0.564 | Smoking status (ever vs never smokers), Smoking status, Smoking initiation (ever regular vs never regular), Smoking initiation (ever regular vs never regular), Age of smoking initiation, Well-being spectrum |
| rs4262121 | 5 | 31078958 | G | C | 0.541 | -0.017 | 0.003 | 2.54E-09 | 0.005 | 0.007 | 0.460 |  |
| rs12521969 | 5 | 61512846 | A | C | 0.489 | -0.022 | 0.003 | 1.68E-15 | 0.000 | 0.007 | 0.956 |  |
| rs389879 | 5 | 77339101 | G | A | 0.809 | 0.021 | 0.004 | 3.52E-09 | -0.002 | 0.008 | 0.852 |  |
| rs1560196 | 5 | 77701982 | G | C | 0.788 | 0.021 | 0.003 | 1.41E-09 | -0.005 | 0.008 | 0.551 |  |
| rs17548339 | 5 | 87687578 | T | G | 0.754 | -0.023 | 0.003 | 4.04E-12 | 0.000 | 0.008 | 0.968 |  |
| rs6882046 | 5 | 87968864 | A | G | 0.739 | -0.020 | 0.003 | 4.58E-10 | 0.005 | 0.008 | 0.486 | General cognitive ability, Educational attainment (years of education), Educational attainment (college completion), Educational attainment, Neuroticism, General factor of neuroticism, Intelligence, Sunburns |
| rs4489042 | 5 | 92428154 | G | C | 0.617 | 0.019 | 0.003 | 1.31E-10 | 0.007 | 0.006 | 0.263 |  |
| rs13167027 | 5 | 93071862 | T | C | 0.814 | 0.028 | 0.004 | 6.13E-12 | -0.002 | 0.009 | 0.815 |  |
| rs40465 | 5 | 103981726 | T | G | 0.676 | -0.032 | 0.003 | 1.57E-20 | -0.008 | 0.007 | 0.279 | Morningness, Number of sexual partners |
| rs288181 | 5 | 107349285 | C | T | 0.670 | 0.017 | 0.003 | 2.26E-08 | 0.011 | 0.007 | 0.118 |  |
| rs62379847 | 5 | 120109119 | A | C | 0.653 | -0.018 | 0.003 | 2.87E-09 | 0.010 | 0.007 | 0.154 |  |
| rs2408225 | 5 | 124262051 | T | C | 0.554 | 0.019 | 0.003 | 3.07E-11 | -0.012 | 0.007 | 0.072 |  |
| rs330340 | 5 | 124911548 | A | C | 0.279 | 0.018 | 0.003 | 1.30E-08 | -0.009 | 0.008 | 0.236 |  |
| rs3756334 | 5 | 140213796 | G | A | 0.482 | -0.017 | 0.003 | 1.03E-09 | 0.020 | 0.007 | 0.002 |  |
| rs2964003 | 5 | 153216733 | A | G | 0.822 | 0.024 | 0.004 | 3.21E-10 | 0.002 | 0.008 | 0.842 |  |
| rs2279020 | 5 | 161322889 | G | A | 0.355 | 0.018 | 0.003 | 1.02E-09 | 0.003 | 0.007 | 0.642 |  |
| rs4434398 | 5 | 164480866 | G | C | 0.462 | -0.029 | 0.003 | 5.98E-19 | 0.009 | 0.007 | 0.190 |  |
| rs4869058 | 5 | 166992247 | T | A | 0.351 | 0.018 | 0.003 | 6.16E-10 | 0.006 | 0.007 | 0.402 | Smoking status (ever vs never smokers) |
| rs12661596 | 6 | 17016400 | A | G | 0.771 | 0.019 | 0.003 | 4.94E-09 | -0.002 | 0.008 | 0.791 |  |
| rs35883476 | 6 | 28368508 | G | C | 0.916 | 0.047 | 0.005 | 1.42E-19 | -0.017 | 0.012 | 0.156 |  |
| rs115938232 | 6 | 29725013 | A | G | 0.946 | -0.046 | 0.006 | 1.14E-14 | 0.002 | 0.017 | 0.896 |  |
| rs10947690 | 6 | 37631768 | A | G | 0.755 | -0.020 | 0.003 | 3.15E-09 | -0.019 | 0.008 | 0.016 | Insomnia symptoms (never/rarely vs. sometimes/usually), Insomnia, Insomnia symptoms (never/rarely vs. usually) |
| rs537362 | 6 | 50650700 | G | C | 0.088 | 0.027 | 0.005 | 2.27E-08 | 0.022 | 0.013 | 0.088 |  |
| rs17752199 | 6 | 51406848 | A | G | 0.891 | 0.028 | 0.005 | 2.30E-09 | 0.012 | 0.010 | 0.242 | Glaucoma (primary open-angle), Glaucoma, Intraocular pressure |
| rs632024 | 6 | 64630933 | T | G | 0.298 | -0.020 | 0.003 | 1.91E-11 | -0.003 | 0.007 | 0.677 |  |
| rs4574611 | 6 | 66602204 | T | A | 0.623 | 0.021 | 0.003 | 7.83E-11 | 0.004 | 0.007 | 0.627 |  |
| rs1414592 | 6 | 76249698 | C | G | 0.492 | -0.017 | 0.003 | 2.69E-09 | 0.005 | 0.007 | 0.433 |  |
| rs12530388 | 6 | 101329173 | A | C | 0.488 | 0.020 | 0.003 | 4.34E-13 | 0.000 | 0.007 | 0.995 | Smoking initiation (ever regular vs never regular) |
| rs1933802 | 6 | 105365891 | C | G | 0.447 | -0.020 | 0.003 | 3.04E-12 | 0.004 | 0.006 | 0.492 |  |
| rs7752418 | 6 | 111787885 | C | T | 0.770 | -0.019 | 0.003 | 2.38E-08 | -0.001 | 0.008 | 0.867 |  |
| rs728017 | 6 | 124292594 | A | G | 0.401 | -0.019 | 0.003 | 7.81E-11 | -0.009 | 0.007 | 0.219 | Insomnia |
| rs12192791 | 6 | 130552266 | G | A | 0.709 | 0.018 | 0.003 | 9.66E-09 | 0.015 | 0.007 | 0.037 |  |
| rs2876520 | 6 | 142996618 | C | G | 0.534 | -0.018 | 0.003 | 4.88E-11 | 0.008 | 0.007 | 0.234 | Well-being spectrum |
| rs9479138 | 6 | 152215199 | T | G | 0.353 | 0.025 | 0.003 | 3.55E-18 | 0.006 | 0.007 | 0.417 |  |
| rs12190823 | 6 | 163092643 | G | A | 0.778 | -0.022 | 0.003 | 1.19E-10 | -0.016 | 0.008 | 0.040 |  |
| rs2322702 | 6 | 165178877 | G | A | 0.555 | 0.018 | 0.003 | 3.16E-10 | 0.009 | 0.007 | 0.213 |  |
| rs10268609 | 7 | 1962163 | G | T | 0.190 | -0.032 | 0.004 | 2.00E-18 | -0.016 | 0.009 | 0.057 |  |
| rs957360 | 7 | 3660918 | C | G | 0.705 | 0.019 | 0.003 | 6.64E-10 | 0.006 | 0.007 | 0.384 |  |
| rs6460905 | 7 | 12283227 | A | G | 0.595 | -0.023 | 0.003 | 3.04E-16 | -0.027 | 0.007 | 2.96E-05 |  |
| rs66974425 | 7 | 13954631 | C | T | 0.751 | 0.018 | 0.003 | 1.64E-08 | -0.004 | 0.008 | 0.599 |  |
| rs7799431 | 7 | 32828281 | G | A | 0.642 | 0.019 | 0.003 | 2.98E-10 | 0.001 | 0.007 | 0.872 |  |
| rs57389877 | 7 | 68412422 | G | A | 0.567 | -0.016 | 0.003 | 1.11E-08 | -0.007 | 0.007 | 0.307 |  |
| rs12707530 | 7 | 82497972 | G | A | 0.660 | -0.020 | 0.003 | 5.12E-11 | -0.002 | 0.007 | 0.774 |  |
| rs9690219 | 7 | 109106510 | C | T | 0.425 | -0.019 | 0.003 | 8.77E-12 | -0.003 | 0.006 | 0.650 |  |
| rs8180817 | 7 | 114047542 | G | C | 0.554 | 0.019 | 0.003 | 2.57E-11 | 0.008 | 0.007 | 0.238 | Insomnia |
| rs1548461 | 7 | 117567576 | T | C | 0.479 | -0.019 | 0.003 | 1.78E-11 | -0.013 | 0.007 | 0.050 |  |
| rs1986692 | 7 | 133743393 | A | G | 0.611 | 0.017 | 0.003 | 2.31E-09 | 0.003 | 0.007 | 0.619 |  |
| rs3812281 | 7 | 135082751 | T | C | 0.605 | -0.017 | 0.003 | 1.49E-09 | -0.004 | 0.007 | 0.545 | Educational attainment (years of education) |
| rs34269011 | 8 | 14220114 | C | T | 0.726 | 0.019 | 0.003 | 3.50E-10 | -0.002 | 0.007 | 0.827 |  |
| rs7017108 | 8 | 20772332 | C | T | 0.570 | -0.018 | 0.003 | 2.28E-10 | -0.002 | 0.007 | 0.761 |  |
| rs6471757 | 8 | 59805738 | G | A | 0.395 | 0.019 | 0.003 | 4.57E-11 | -0.009 | 0.007 | 0.184 |  |
| rs1425794 | 8 | 64806567 | G | T | 0.551 | -0.016 | 0.003 | 6.46E-09 | 0.003 | 0.007 | 0.639 |  |
| rs7837935 | 8 | 65562019 | T | G | 0.145 | -0.030 | 0.004 | 1.33E-14 | -0.003 | 0.010 | 0.770 | Neurociticism |
| rs1433965 | 8 | 77633344 | T | C | 0.836 | -0.022 | 0.004 | 5.12E-09 | -0.009 | 0.009 | 0.306 |  |
| rs4478545 | 8 | 94672542 | G | A | 0.712 | 0.018 | 0.003 | 2.93E-09 | 0.005 | 0.007 | 0.475 |  |
| rs6470670 | 8 | 129913448 | T | C | 0.299 | -0.020 | 0.003 | 2.57E-10 | -0.009 | 0.007 | 0.203 |  |
| rs10441718 | 9 | 2989299 | C | T | 0.402 | -0.018 | 0.003 | 1.57E-10 | -0.007 | 0.007 | 0.293 |  |
| rs1931388 | 9 | 11203149 | A | G | 0.594 | 0.024 | 0.003 | 4.36E-16 | 0.004 | 0.007 | 0.597 |  |
| rs6474807 | 9 | 14061876 | G | C | 0.290 | -0.017 | 0.003 | 4.46E-08 | -0.006 | 0.008 | 0.460 |  |
| rs12003380 | 9 | 17135434 | G | C | 0.562 | 0.017 | 0.003 | 6.68E-09 | 0.006 | 0.007 | 0.401 |  |
| rs3793577 | 9 | 23737627 | A | G | 0.469 | -0.022 | 0.003 | 2.07E-15 | -0.004 | 0.007 | 0.580 | Well-being spectrum, Neuroticism, Positive affect, Life satisfaction |
| rs4531131 | 9 | 26723702 | A | G | 0.819 | 0.022 | 0.004 | 3.41E-08 | 0.017 | 0.009 | 0.051 |  |
| rs13296641 | 9 | 31160243 | G | A | 0.682 | 0.021 | 0.003 | 1.49E-12 | 0.003 | 0.007 | 0.703 |  |
| rs1329572 | 9 | 37001471 | T | A | 0.600 | -0.025 | 0.003 | 1.41E-17 | -0.009 | 0.007 | 0.177 | Well-being spectrum |
| rs11138500 | 9 | 82737534 | G | A | 0.295 | -0.018 | 0.003 | 3.21E-08 | 0.006 | 0.008 | 0.444 |  |
| rs7023933 | 9 | 96225478 | A | T | 0.834 | -0.026 | 0.004 | 2.31E-12 | -0.009 | 0.009 | 0.294 | Worry |
| rs182923402 | 9 | 98299677 | T | A | 0.915 | -0.030 | 0.005 | 4.73E-09 | -0.025 | 0.011 | 0.027 |  |
| rs2418449 | 9 | 119731359 | T | C | 0.709 | 0.022 | 0.003 | 1.15E-12 | -0.001 | 0.007 | 0.882 |  |
| rs913930 | 9 | 120484009 | G | A | 0.359 | 0.018 | 0.003 | 1.41E-09 | -0.005 | 0.007 | 0.510 | Neuroticism |
| rs12553064 | 9 | 122660054 | C | A | 0.390 | -0.020 | 0.003 | 3.36E-12 | -0.002 | 0.007 | 0.754 | Educational attainment |
| rs667138 | 9 | 126658613 | A | C | 0.852 | -0.025 | 0.004 | 4.23E-09 | -0.001 | 0.010 | 0.955 |  |
| rs10858298 | 9 | 137839527 | G | A | 0.299 | 0.018 | 0.003 | 1.02E-08 | -0.001 | 0.008 | 0.930 |  |
| rs112535944 | 9 | 140278206 | C | A | 0.841 | -0.045 | 0.007 | 1.77E-10 | -0.022 | 0.012 | 0.062 |  |
| rs4880918 | 10 | 1780944 | C | G | 0.484 | 0.017 | 0.003 | 1.98E-09 | 0.013 | 0.007 | 0.053 |  |
| rs1993849 | 10 | 67796008 | G | A | 0.194 | 0.022 | 0.004 | 5.72E-10 | 0.003 | 0.008 | 0.733 |  |
| rs4515864 | 10 | 68530073 | G | A | 0.201 | 0.020 | 0.004 | 9.08E-09 | 0.007 | 0.008 | 0.397 |  |
| rs3088142 | 10 | 76854564 | C | T | 0.575 | -0.018 | 0.003 | 4.92E-11 | -0.003 | 0.007 | 0.618 | Body mass index |
| rs1909696 | 10 | 77582203 | G | T | 0.330 | -0.018 | 0.003 | 2.77E-09 | -0.007 | 0.007 | 0.318 |  |
| rs61691222 | 10 | 104420754 | C | T | 0.774 | -0.019 | 0.003 | 3.72E-09 | 0.008 | 0.008 | 0.321 |  |
| rs17186548 | 10 | 106550944 | G | A | 0.788 | 0.020 | 0.003 | 9.13E-09 | -0.009 | 0.008 | 0.273 |  |
| rs1021363 | 10 | 106610839 | A | G | 0.339 | 0.027 | 0.003 | 5.32E-20 | -0.003 | 0.007 | 0.688 |  |
| rs111442587 | 10 | 114984089 | C | G | 0.976 | -0.053 | 0.009 | 1.54E-08 | -0.036 | 0.024 | 0.131 |  |
| rs10749240 | 10 | 118914837 | T | C | 0.596 | -0.016 | 0.003 | 4.96E-08 | -0.002 | 0.007 | 0.831 |  |
| rs12285422 | 11 | 24591463 | G | C | 0.627 | 0.019 | 0.003 | 1.04E-08 | 0.006 | 0.007 | 0.351 |  |
| rs11030380 | 11 | 28591168 | A | T | 0.399 | -0.022 | 0.003 | 1.02E-14 | -0.004 | 0.007 | 0.502 | Alcohol consumption (drinks per week) |
| rs324300 | 11 | 29674831 | G | A | 0.619 | -0.017 | 0.003 | 1.80E-09 | 0.003 | 0.007 | 0.676 |  |
| rs3026389 | 11 | 31813529 | C | G | 0.205 | 0.023 | 0.003 | 4.90E-12 | -0.005 | 0.008 | 0.581 | Neurociticism, Feeling worry |
| rs80067584 | 11 | 32597748 | C | A | 0.947 | 0.038 | 0.006 | 1.10E-09 | 0.002 | 0.015 | 0.901 |  |
| rs1825119 | 11 | 38556991 | A | G | 0.646 | -0.018 | 0.003 | 1.15E-09 | 0.001 | 0.007 | 0.858 |  |
| rs112181005 | 11 | 47010491 | G | A | 0.855 | 0.025 | 0.004 | 1.51E-09 | -0.020 | 0.009 | 0.032 |  |
| rs2509805 | 11 | 57650796 | T | C | 0.328 | 0.023 | 0.003 | 5.31E-14 | 0.014 | 0.007 | 0.054 |  |
| rs102275 | 11 | 61557803 | T | C | 0.640 | -0.020 | 0.003 | 1.96E-12 | 0.010 | 0.007 | 0.130 | Serum metabolite ratios in chronic kidney disease, Plasma omega-6 polyunsaturated fatty acid levels (arachidonic acid), Plasma omega-3 polyunsaturated fatty acid levels (docosapentaenoic acid), Oleic acid (18:1n-9) levels, Glycerophospholipid levels, Trans fatty acid levels, Serum metabolite concentrations in chronic kidney disease, Metabolic syndrome, Red blood cell count, Plasma omega-3 polyunsaturated fatty acid levels (alphalinolenic acid), Palmitoleic acid (16:1n-7) levels, Irritable mood, Stearic acid (18:0) levels, Phospholipid levels (plasma), Crohn's disease, Metabolite levels |
| rs11600628 | 11 | 63976027 | C | T | 0.845 | 0.022 | 0.004 | 1.25E-08 | 0.019 | 0.009 | 0.041 |  |
| rs12789028 | 11 | 65326154 | G | A | 0.825 | -0.022 | 0.004 | 8.96E-10 | -0.024 | 0.009 | 0.006 |  |
| rs7117514 | 11 | 70544937 | A | G | 0.547 | -0.015 | 0.003 | 4.51E-08 | -0.007 | 0.007 | 0.304 |  |
| rs4753209 | 11 | 88743208 | T | A | 0.439 | 0.024 | 0.003 | 9.57E-16 | 0.012 | 0.007 | 0.110 |  |
| rs4937872 | 11 | 112827715 | A | G | 0.533 | -0.016 | 0.003 | 1.53E-08 | -0.018 | 0.008 | 0.021 | Feeling tense, Worry |
| rs61902811 | 11 | 113370758 | G | A | 0.665 | 0.026 | 0.003 | 1.04E-18 | 0.010 | 0.007 | 0.138 |  |
| rs7130926 | 11 | 113496928 | T | C | 0.863 | -0.027 | 0.004 | 1.10E-10 | -0.030 | 0.010 | 0.003 |  |
| rs7101595 | 11 | 126629031 | C | T | 0.437 | -0.016 | 0.003 | 1.74E-08 | -0.003 | 0.006 | 0.699 |  |
| rs57344483 | 11 | 127022560 | A | G | 0.915 | -0.031 | 0.005 | 1.30E-08 | -0.001 | 0.012 | 0.942 |  |
| rs612823 | 11 | 133834104 | T | C | 0.591 | 0.021 | 0.003 | 1.07E-12 | 0.010 | 0.007 | 0.173 |  |
| rs11062170 | 12 | 2348844 | G | C | 0.675 | -0.017 | 0.003 | 9.56E-09 | -0.020 | 0.007 | 0.003 |  |
| rs10491990 | 12 | 11939528 | G | A | 0.838 | 0.022 | 0.004 | 2.75E-08 | 0.007 | 0.009 | 0.473 |  |
| rs7968921 | 12 | 23960177 | T | C | 0.405 | -0.018 | 0.003 | 3.99E-10 | -0.010 | 0.007 | 0.154 |  |
| rs16926971 | 12 | 24169797 | T | C | 0.792 | 0.021 | 0.004 | 2.30E-09 | 0.000 | 0.008 | 0.993 |  |
| rs11612312 | 12 | 52349088 | T | C | 0.798 | -0.021 | 0.004 | 8.87E-10 | 0.015 | 0.008 | 0.073 | Positive affect |
| rs73123350 | 12 | 58050250 | G | T | 0.579 | -0.016 | 0.003 | 1.91E-08 | -0.013 | 0.007 | 0.042 |  |
| rs56051015 | 12 | 84497937 | C | G | 0.811 | -0.022 | 0.004 | 2.24E-10 | 0.006 | 0.008 | 0.482 |  |
| rs2292996 | 12 | 103556972 | T | C | 0.483 | -0.016 | 0.003 | 1.66E-08 | 0.002 | 0.007 | 0.724 |  |
| rs521048 | 12 | 118830958 | A | G | 0.265 | -0.018 | 0.003 | 2.98E-08 | 0.000 | 0.008 | 0.972 |  |
| rs3213572 | 12 | 121205078 | G | A | 0.530 | -0.021 | 0.003 | 1.02E-13 | 0.005 | 0.006 | 0.453 |  |
| rs7334101 | 13 | 31900245 | G | A | 0.217 | -0.021 | 0.003 | 9.42E-10 | 0.001 | 0.008 | 0.902 |  |
| rs9536383 | 13 | 53865563 | C | T | 0.686 | -0.024 | 0.003 | 9.92E-15 | 0.014 | 0.007 | 0.046 |  |
| rs7988498 | 13 | 66946477 | C | T | 0.503 | 0.019 | 0.003 | 1.88E-11 | -0.007 | 0.007 | 0.325 |  |
| rs7982263 | 13 | 80061074 | T | C | 0.404 | -0.015 | 0.003 | 4.30E-08 | -0.003 | 0.007 | 0.689 |  |
| rs508502 | 13 | 80921519 | C | T | 0.697 | 0.020 | 0.003 | 3.06E-10 | 0.001 | 0.008 | 0.941 |  |
| rs73532287 | 13 | 85234525 | G | T | 0.800 | -0.020 | 0.004 | 2.12E-08 | -0.009 | 0.008 | 0.305 |  |
| rs9561328 | 13 | 94009312 | G | T | 0.873 | -0.025 | 0.004 | 1.51E-09 | -0.015 | 0.010 | 0.117 |  |
| rs7317645 | 13 | 97130674 | T | C | 0.162 | -0.024 | 0.004 | 1.35E-09 | -0.020 | 0.009 | 0.030 |  |
| rs4772087 | 13 | 99115041 | C | T | 0.620 | -0.020 | 0.003 | 8.25E-12 | 0.002 | 0.007 | 0.757 |  |
| rs11157241 | 14 | 42051771 | T | C | 0.506 | 0.023 | 0.003 | 2.12E-16 | 0.008 | 0.007 | 0.250 |  |
| rs2274794 | 14 | 57283934 | C | T | 0.666 | 0.021 | 0.003 | 8.09E-12 | 0.010 | 0.007 | 0.148 |  |
| rs10148598 | 14 | 60147659 | C | T | 0.241 | 0.019 | 0.003 | 1.56E-09 | 0.003 | 0.008 | 0.673 |  |
| rs10142766 | 14 | 64780454 | A | G | 0.445 | -0.019 | 0.003 | 2.77E-11 | 0.010 | 0.007 | 0.113 |  |
| rs11621186 | 14 | 75120628 | C | T | 0.500 | -0.022 | 0.003 | 1.45E-15 | -0.006 | 0.006 | 0.341 |  |
| rs3008497 | 14 | 85038175 | G | T | 0.682 | 0.016 | 0.003 | 4.57E-08 | 0.001 | 0.007 | 0.901 |  |
| rs1286060 | 14 | 91458156 | G | A | 0.250 | -0.018 | 0.003 | 4.34E-08 | 0.024 | 0.008 | 0.001 |  |
| rs7141014 | 14 | 98667928 | T | C | 0.803 | -0.021 | 0.004 | 3.00E-09 | -0.006 | 0.008 | 0.478 |  |
| rs9671376 | 14 | 103370163 | C | T | 0.763 | -0.020 | 0.003 | 2.99E-09 | -0.015 | 0.008 | 0.045 |  |
| rs10149470 | 14 | 104017953 | A | G | 0.485 | -0.023 | 0.003 | 1.96E-15 | 0.008 | 0.007 | 0.252 | Extremely high intelligence, Autism and major depressive disorder, Cognitive ability, Intelligence |
| rs12898464 | 15 | 29853387 | C | T | 0.118 | 0.025 | 0.005 | 4.26E-08 | -0.007 | 0.012 | 0.583 |  |
| rs11073153 | 15 | 36425448 | G | T | 0.403 | -0.016 | 0.003 | 4.94E-08 | 0.003 | 0.007 | 0.669 |  |
| rs8037355 | 15 | 37643831 | C | T | 0.462 | 0.018 | 0.003 | 1.07E-10 | -0.010 | 0.007 | 0.140 |  |
| rs56059718 | 15 | 38836777 | C | A | 0.806 | -0.021 | 0.004 | 2.67E-09 | 0.002 | 0.008 | 0.773 |  |
| rs34488670 | 15 | 47684936 | T | C | 0.785 | -0.024 | 0.003 | 2.40E-12 | 0.003 | 0.008 | 0.721 | Age of smoking initiation, Risk-taking tendency (4-domain principal component model), Number of sexual partners |
| rs8040348 | 15 | 47999319 | C | T | 0.615 | 0.016 | 0.003 | 2.76E-08 | 0.001 | 0.007 | 0.910 |  |
| rs79714693 | 15 | 63993463 | C | T | 0.794 | -0.023 | 0.004 | 4.81E-11 | -0.017 | 0.008 | 0.030 |  |
| rs4776729 | 15 | 66399197 | A | G | 0.869 | -0.026 | 0.004 | 4.13E-10 | -0.006 | 0.010 | 0.571 |  |
| rs2060337 | 15 | 70615407 | T | G | 0.472 | 0.018 | 0.003 | 6.36E-10 | 0.010 | 0.007 | 0.142 | Highest math class taken, Self-reported math ability |
| rs8030745 | 15 | 74133091 | T | C | 0.879 | 0.029 | 0.004 | 1.53E-11 | 0.003 | 0.010 | 0.735 |  |
| rs12900865 | 15 | 78075910 | G | A | 0.425 | -0.017 | 0.003 | 1.43E-09 | -0.004 | 0.007 | 0.522 |  |
| rs11855600 | 15 | 88963259 | C | T | 0.828 | 0.021 | 0.004 | 2.55E-08 | 0.005 | 0.009 | 0.564 |  |
| rs4702 | 15 | 91426560 | G | A | 0.452 | 0.020 | 0.003 | 1.66E-12 | -0.016 | 0.007 | 0.018 | Insomnia, Autism spectrum disorder or schizophrenia, General risk tolerance, Number of sexual partners, Age at first sexual intercourse, Schizophrenia, Feeling hurt |
| rs7193263 | 16 | 6315880 | G | A | 0.340 | 0.020 | 0.003 | 3.51E-11 | 0.005 | 0.008 | 0.525 |  |
| rs11077206 | 16 | 7667332 | G | C | 0.373 | -0.016 | 0.003 | 2.63E-08 | 0.001 | 0.007 | 0.940 |  |
| rs7200826 | 16 | 13066833 | C | T | 0.730 | -0.024 | 0.003 | 1.07E-13 | 0.007 | 0.008 | 0.346 | Well-being spectrum, Neuroticism |
| rs7184632 | 16 | 13750997 | C | G | 0.284 | 0.027 | 0.004 | 4.88E-14 | 0.001 | 0.007 | 0.917 |  |
| rs2369818 | 16 | 21614009 | C | T | 0.574 | -0.021 | 0.003 | 3.25E-13 | 0.011 | 0.007 | 0.133 |  |
| rs4785307 | 16 | 49494029 | A | G | 0.426 | 0.018 | 0.003 | 8.54E-10 | -0.001 | 0.007 | 0.877 |  |
| rs4784296 | 16 | 51479349 | G | A | 0.532 | 0.015 | 0.003 | 3.94E-08 | 0.000 | 0.007 | 0.992 |  |
| rs11643192 | 16 | 72214276 | C | A | 0.594 | -0.017 | 0.003 | 8.51E-09 | -0.009 | 0.007 | 0.196 | Ease of getting up in the morning |
| rs11644513 | 16 | 82868852 | C | T | 0.662 | 0.017 | 0.003 | 1.44E-08 | 0.001 | 0.007 | 0.939 |  |
| rs2585399 | 17 | 8054759 | G | A | 0.395 | -0.017 | 0.003 | 1.21E-08 | -0.007 | 0.007 | 0.336 |  |
| rs75581564 | 17 | 27363750 | G | A | 0.883 | -0.025 | 0.004 | 1.06E-08 | 0.000 | 0.010 | 0.977 |  |
| rs412891 | 17 | 31545583 | G | T | 0.264 | -0.020 | 0.003 | 1.93E-10 | 0.018 | 0.008 | 0.020 |  |
| rs60856912 | 17 | 65892343 | G | T | 0.819 | -0.026 | 0.004 | 1.61E-11 | -0.013 | 0.009 | 0.130 | Endometrial cancer, Smoking initiation (ever regular vs never regular) |
| rs10460051 | 18 | 31413679 | C | T | 0.491 | -0.019 | 0.003 | 4.19E-12 | 0.000 | 0.006 | 0.967 | Neurociticism, Neuroticism |
| rs12967855 | 18 | 35138245 | A | G | 0.321 | 0.026 | 0.003 | 2.86E-18 | -0.001 | 0.007 | 0.881 | Educational attainment (years of education), Depressed affect |
| rs73428490 | 18 | 36614279 | C | A | 0.913 | 0.028 | 0.005 | 7.47E-09 | 0.023 | 0.011 | 0.036 |  |
| rs4267411 | 18 | 39305256 | C | T | 0.821 | 0.022 | 0.004 | 4.84E-10 | 0.016 | 0.009 | 0.067 |  |
| rs7230285 | 18 | 50723080 | G | A | 0.499 | -0.024 | 0.003 | 1.17E-17 | -0.002 | 0.006 | 0.772 |  |
| rs1819881 | 18 | 52471573 | C | G | 0.209 | -0.022 | 0.004 | 1.08E-09 | 0.009 | 0.008 | 0.301 |  |
| rs4131791 | 18 | 52747871 | C | T | 0.587 | 0.021 | 0.003 | 4.80E-14 | 0.009 | 0.007 | 0.154 |  |
| rs12967143 | 18 | 53099012 | G | C | 0.294 | 0.025 | 0.003 | 5.94E-16 | -0.008 | 0.007 | 0.302 | Well-being spectrum |
| rs7241572 | 18 | 77580712 | G | A | 0.797 | -0.026 | 0.004 | 1.31E-11 | 0.002 | 0.009 | 0.824 |  |
| rs1555133 | 20 | 31048382 | G | A | 0.656 | -0.017 | 0.003 | 9.91E-09 | 0.004 | 0.007 | 0.574 | Monocyte count |
| rs6131010 | 20 | 44724305 | A | G | 0.268 | 0.024 | 0.003 | 9.85E-14 | 0.022 | 0.008 | 0.004 | Well-being spectrum |
| rs910187 | 20 | 45841052 | G | A | 0.638 | 0.020 | 0.003 | 6.25E-11 | -0.006 | 0.007 | 0.374 | Insomnia, Neuroticism, Migraine, Well-being spectrum |
| rs2273653 | 20 | 47770756 | A | C | 0.587 | 0.018 | 0.003 | 2.48E-10 | 0.012 | 0.007 | 0.062 | Well-being spectrum |
| rs520838 | 20 | 59002955 | C | A | 0.756 | -0.018 | 0.003 | 2.95E-08 | 0.008 | 0.007 | 0.277 |  |
| rs9974713 | 21 | 38817699 | T | C | 0.233 | -0.019 | 0.004 | 3.85E-08 | -0.005 | 0.008 | 0.524 |  |
| rs2838788 | 21 | 46542374 | T | C | 0.612 | 0.016 | 0.003 | 2.09E-08 | -0.004 | 0.007 | 0.562 |  |
| rs2273085 | 22 | 41615376 | C | T | 0.710 | -0.025 | 0.003 | 2.85E-16 | -0.010 | 0.007 | 0.178 | Neuroticism |
| rs9627391 | 22 | 46447097 | C | T | 0.683 | 0.017 | 0.003 | 1.28E-08 | 0.001 | 0.008 | 0.918 |  |
| SNP, single nucleotide polymorphisms; CHR, chromosome; BP, physical position of SNP (base-pairs); A1, effect allele; A2, alternative allele; EAF: effect allele frequency; Beta, effect allele beta coefficient; SE, standard error of the beta coefficient. | | | | | | | | | | | | |

| **Supplementary Table 2. Characteristics of genetic instruments of depression and their effect sizes with ischemic stroke.** | | | | | | | | | | | | |
| --- | --- | --- | --- | --- | --- | --- | --- | --- | --- | --- | --- | --- |
| SNP | CHR | BP | A1 | A2 | EAF | Exposure | | | Outcome | | | Pleiotropic traits |
|  |  |  |  |  |  | Beta | SE | P | Beta | SE | P |  |
| rs301817 | 1 | 8503379 | C | A | 0.408 | 0.024 | 0.003 | 3.53E-13 | -0.023 | 0.008 | 0.002 |  |
| rs2089358 | 1 | 37194103 | C | T | 0.305 | 0.025 | 0.003 | 2.65E-15 | 0.003 | 0.008 | 0.735 | Insomnia |
| rs1466887 | 1 | 37709328 | C | T | 0.441 | 0.016 | 0.003 | 1.06E-08 | 0.008 | 0.007 | 0.263 |  |
| rs12141363 | 1 | 47696581 | C | A | 0.399 | 0.016 | 0.003 | 1.91E-08 | 0.022 | 0.007 | 0.002 |  |
| rs11579246 | 1 | 50559162 | A | G | 0.894 | 0.028 | 0.005 | 3.71E-09 | 0.008 | 0.011 | 0.456 |  |
| rs1890946 | 1 | 52342427 | T | C | 0.469 | -0.019 | 0.003 | 4.81E-12 | -0.003 | 0.007 | 0.638 |  |
| rs7531118 | 1 | 72837239 | T | C | 0.464 | -0.029 | 0.003 | 4.90E-24 | 0.006 | 0.008 | 0.400 | Weight, Waist circumference, Obesity, Intelligence, Body mass index, Hip circumference |
| rs4571923 | 1 | 73736562 | G | A | 0.513 | -0.023 | 0.003 | 9.77E-17 | -0.001 | 0.007 | 0.874 |  |
| rs10874020 | 1 | 79593241 | T | C | 0.300 | -0.017 | 0.003 | 4.90E-08 | 0.010 | 0.009 | 0.244 |  |
| rs2389015 | 1 | 80799190 | G | A | 0.731 | -0.022 | 0.003 | 3.16E-12 | -0.018 | 0.009 | 0.032 |  |
| rs7513962 | 1 | 96952532 | C | T | 0.480 | -0.018 | 0.003 | 4.30E-09 | -0.013 | 0.007 | 0.067 |  |
| rs16846561 | 1 | 173869111 | C | G | 0.896 | 0.029 | 0.005 | 2.15E-10 | -0.016 | 0.012 | 0.169 |  |
| rs10913112 | 1 | 175913828 | C | T | 0.628 | 0.022 | 0.003 | 7.69E-13 | 0.011 | 0.008 | 0.177 |  |
| rs72724899 | 1 | 177408112 | C | T | 0.924 | -0.034 | 0.005 | 6.71E-12 | 0.019 | 0.014 | 0.177 |  |
| rs74934992 | 1 | 191255088 | G | T | 0.956 | -0.043 | 0.007 | 3.05E-09 | -0.013 | 0.018 | 0.454 |  |
| rs2358493 | 1 | 197816439 | G | A | 0.491 | -0.023 | 0.003 | 8.97E-17 | 0.009 | 0.007 | 0.199 |  |
| rs57691884 | 1 | 198234477 | G | A | 0.924 | -0.030 | 0.005 | 1.35E-08 | -0.005 | 0.013 | 0.678 |  |
| rs12131852 | 1 | 214433323 | C | G | 0.712 | 0.017 | 0.003 | 2.52E-08 | -0.007 | 0.008 | 0.378 |  |
| rs2576241 | 1 | 217100192 | C | A | 0.461 | 0.017 | 0.003 | 5.45E-10 | -0.003 | 0.007 | 0.720 |  |
| rs4846898 | 1 | 230252218 | A | G | 0.590 | -0.017 | 0.003 | 8.16E-10 | -0.003 | 0.007 | 0.626 |  |
| rs12619197 | 2 | 22566548 | G | A | 0.540 | -0.021 | 0.003 | 2.14E-13 | -0.004 | 0.007 | 0.563 |  |
| rs4666020 | 2 | 28046028 | G | A | 0.799 | 0.019 | 0.004 | 4.39E-08 | 0.003 | 0.009 | 0.757 |  |
| rs13031157 | 2 | 51216810 | T | C | 0.571 | 0.019 | 0.003 | 1.40E-11 | 0.012 | 0.007 | 0.101 |  |
| rs7599039 | 2 | 51578355 | T | C | 0.790 | 0.020 | 0.003 | 6.22E-09 | 0.001 | 0.009 | 0.911 |  |
| rs4971586 | 2 | 52315585 | T | A | 0.523 | 0.019 | 0.003 | 3.81E-11 | 0.000 | 0.007 | 0.950 |  |
| rs11682175 | 2 | 57987593 | T | C | 0.540 | -0.021 | 0.003 | 2.85E-14 | -0.007 | 0.007 | 0.288 | Sleep duration, Schizophrenia, Autism spectrum disorder or schizophrenia, Irritable mood |
| rs34829058 | 2 | 60182065 | T | C | 0.754 | -0.018 | 0.003 | 1.68E-08 | -0.005 | 0.008 | 0.557 |  |
| rs4671458 | 2 | 63374063 | C | T | 0.784 | 0.020 | 0.004 | 1.16E-08 | -0.002 | 0.008 | 0.781 | Subjective well-being |
| rs10173072 | 2 | 71660804 | C | T | 0.566 | 0.016 | 0.003 | 1.09E-08 | -0.010 | 0.007 | 0.141 |  |
| rs7585722 | 2 | 86819128 | T | C | 0.857 | -0.022 | 0.004 | 2.29E-08 | 0.008 | 0.010 | 0.426 |  |
| rs2570485 | 2 | 104464467 | C | T | 0.753 | 0.022 | 0.003 | 1.72E-11 | 0.006 | 0.008 | 0.449 |  |
| rs62170849 | 2 | 125012926 | T | C | 0.710 | 0.020 | 0.003 | 1.31E-10 | 0.005 | 0.008 | 0.484 |  |
| rs13020607 | 2 | 127163515 | T | C | 0.843 | 0.022 | 0.004 | 1.20E-08 | 0.005 | 0.010 | 0.622 |  |
| rs12477455 | 2 | 144079060 | C | T | 0.547 | 0.017 | 0.003 | 6.35E-09 | 0.001 | 0.007 | 0.899 |  |
| rs1320138 | 2 | 144158287 | T | C | 0.433 | 0.019 | 0.003 | 7.50E-12 | 0.001 | 0.007 | 0.855 |  |
| rs835302 | 2 | 157123393 | G | T | 0.215 | -0.024 | 0.003 | 1.28E-12 | -0.001 | 0.009 | 0.906 |  |
| rs10205665 | 2 | 161480003 | G | A | 0.669 | -0.017 | 0.003 | 2.55E-08 | -0.010 | 0.007 | 0.196 |  |
| rs1267079 | 2 | 162033854 | T | C | 0.695 | 0.022 | 0.003 | 2.47E-13 | 0.007 | 0.008 | 0.356 |  |
| rs908670 | 2 | 172690180 | T | C | 0.729 | -0.017 | 0.003 | 2.49E-08 | 0.004 | 0.008 | 0.635 |  |
| rs4972656 | 2 | 175240292 | C | T | 0.425 | -0.021 | 0.003 | 2.12E-13 | 0.000 | 0.007 | 0.976 |  |
| rs6713469 | 2 | 195882599 | A | G | 0.832 | 0.021 | 0.004 | 1.93E-08 | 0.008 | 0.010 | 0.415 |  |
| rs6715105 | 2 | 198445601 | T | C | 0.337 | 0.019 | 0.003 | 4.31E-10 | 0.013 | 0.008 | 0.076 |  |
| rs55772859 | 2 | 208042581 | C | A | 0.677 | -0.022 | 0.003 | 2.46E-13 | 0.007 | 0.008 | 0.373 | Insomnia |
| rs7576085 | 2 | 210187488 | C | T | 0.451 | -0.016 | 0.003 | 1.42E-08 | 0.008 | 0.007 | 0.278 |  |
| rs1801251 | 2 | 233633460 | G | A | 0.669 | -0.018 | 0.003 | 5.79E-10 | -0.018 | 0.008 | 0.014 | Coronary artery disease |
| rs4663414 | 2 | 235352188 | T | G | 0.378 | 0.016 | 0.003 | 1.60E-08 | 0.003 | 0.008 | 0.690 |  |
| rs6443178 | 3 | 8278215 | C | T | 0.462 | 0.016 | 0.003 | 4.57E-08 | -0.006 | 0.007 | 0.428 |  |
| rs6800583 | 3 | 16851755 | G | A | 0.632 | -0.016 | 0.003 | 1.41E-08 | 0.003 | 0.007 | 0.676 |  |
| rs77463213 | 3 | 43432024 | C | T | 0.849 | -0.022 | 0.004 | 1.16E-08 | -0.015 | 0.010 | 0.126 |  |
| rs13058913 | 3 | 44732013 | A | T | 0.682 | -0.025 | 0.003 | 2.46E-16 | -0.006 | 0.008 | 0.456 |  |
| rs7636868 | 3 | 45373208 | G | A | 0.291 | 0.018 | 0.003 | 4.26E-09 | -0.003 | 0.008 | 0.684 |  |
| rs7617480 | 3 | 49210732 | A | C | 0.216 | 0.032 | 0.003 | 1.21E-20 | -0.005 | 0.009 | 0.571 | Smoking cessation, Subjective well-being, Menarche (age at onset) |
| rs1010553 | 3 | 52540773 | T | C | 0.523 | -0.019 | 0.003 | 1.63E-11 | -0.006 | 0.007 | 0.430 | Hematocrit |
| rs17216573 | 3 | 56282978 | T | C | 0.853 | 0.022 | 0.004 | 4.16E-08 | 0.015 | 0.010 | 0.129 | Lung function (FEV1/FVC) |
| rs1470035 | 3 | 61184321 | G | A | 0.591 | -0.017 | 0.003 | 2.09E-09 | -0.005 | 0.007 | 0.497 |  |
| rs116310555 | 3 | 71814431 | T | C | 0.882 | -0.025 | 0.004 | 8.96E-09 | -0.023 | 0.011 | 0.030 |  |
| rs1597076 | 3 | 108484536 | G | A | 0.673 | -0.017 | 0.003 | 2.34E-08 | -0.009 | 0.007 | 0.224 |  |
| rs6783233 | 3 | 117509984 | C | T | 0.731 | -0.019 | 0.003 | 6.38E-10 | -0.003 | 0.008 | 0.691 |  |
| rs7618264 | 3 | 132680873 | A | T | 0.453 | 0.016 | 0.003 | 8.93E-09 | -0.008 | 0.007 | 0.272 |  |
| rs827137 | 3 | 158008124 | C | T | 0.559 | -0.026 | 0.003 | 3.48E-16 | 0.011 | 0.007 | 0.124 |  |
| rs536445 | 3 | 173120103 | C | T | 0.475 | -0.019 | 0.003 | 1.49E-11 | -0.023 | 0.007 | 0.001 |  |
| rs2298969 | 4 | 3186244 | A | G | 0.483 | 0.017 | 0.003 | 3.05E-09 | 0.004 | 0.007 | 0.573 |  |
| rs9306914 | 4 | 34552161 | A | T | 0.838 | -0.025 | 0.005 | 2.77E-08 | -0.005 | 0.010 | 0.587 |  |
| rs114851235 | 4 | 59833409 | A | G | 0.946 | -0.043 | 0.007 | 9.34E-11 | -0.005 | 0.015 | 0.761 |  |
| rs34207047 | 4 | 80199323 | C | A | 0.478 | 0.021 | 0.003 | 1.42E-13 | -0.004 | 0.007 | 0.607 |  |
| rs6813752 | 4 | 91099481 | T | A | 0.373 | -0.018 | 0.003 | 1.05E-09 | 0.002 | 0.008 | 0.802 |  |
| rs6845263 | 4 | 102276682 | T | C | 0.500 | 0.015 | 0.003 | 3.79E-08 | 0.013 | 0.007 | 0.076 | Superior crus of antihelix expression |
| rs45510091 | 4 | 123186393 | A | G | 0.949 | 0.045 | 0.007 | 4.03E-12 | 0.008 | 0.016 | 0.633 | Feeling miserable |
| rs35553410 | 4 | 131237381 | T | C | 0.748 | -0.021 | 0.004 | 1.52E-09 | -0.009 | 0.009 | 0.321 |  |
| rs6536630 | 4 | 140919035 | T | A | 0.465 | 0.018 | 0.003 | 1.12E-09 | 0.016 | 0.007 | 0.024 |  |
| rs17611770 | 4 | 176851045 | A | T | 0.799 | -0.020 | 0.004 | 1.78E-08 | -0.019 | 0.009 | 0.032 | Neuroticism |
| rs7659414 | 4 | 177350956 | A | C | 0.591 | -0.017 | 0.003 | 8.54E-09 | -0.004 | 0.007 | 0.544 |  |
| rs2220599 | 5 | 7378854 | C | G | 0.649 | -0.019 | 0.003 | 2.40E-10 | 0.003 | 0.007 | 0.641 | Subjective well-being |
| rs7700453 | 5 | 20126890 | G | C | 0.472 | 0.016 | 0.003 | 3.09E-08 | 0.017 | 0.007 | 0.020 |  |
| rs10805794 | 5 | 27024536 | C | A | 0.487 | 0.017 | 0.003 | 2.55E-09 | -0.005 | 0.007 | 0.442 |  |
| rs12517438 | 5 | 30842054 | T | G | 0.469 | -0.019 | 0.003 | 3.05E-11 | 0.007 | 0.007 | 0.320 | Smoking status (ever vs never smokers), Smoking status, Smoking initiation (ever regular vs never regular), Smoking initiation (ever regular vs never regular), Age of smoking initiation, Well-being spectrum |
| rs4262121 | 5 | 31078958 | G | C | 0.541 | -0.017 | 0.003 | 2.54E-09 | 0.009 | 0.007 | 0.231 |  |
| rs12521969 | 5 | 61512846 | A | C | 0.489 | -0.022 | 0.003 | 1.68E-15 | -0.001 | 0.007 | 0.879 |  |
| rs389879 | 5 | 77339101 | G | A | 0.809 | 0.021 | 0.004 | 3.52E-09 | 0.000 | 0.009 | 0.983 |  |
| rs1560196 | 5 | 77701982 | G | C | 0.788 | 0.021 | 0.003 | 1.41E-09 | -0.007 | 0.009 | 0.438 |  |
| rs17548339 | 5 | 87687578 | T | G | 0.754 | -0.023 | 0.003 | 4.04E-12 | -0.003 | 0.009 | 0.784 |  |
| rs6882046 | 5 | 87968864 | A | G | 0.739 | -0.020 | 0.003 | 4.58E-10 | 0.008 | 0.008 | 0.294 | General cognitive ability, Educational attainment (years of education), Educational attainment (college completion), Educational attainment, Neuroticism, General factor of neuroticism, Intelligence, Sunburns |
| rs4489042 | 5 | 92428154 | G | C | 0.617 | 0.019 | 0.003 | 1.31E-10 | 0.006 | 0.007 | 0.432 |  |
| rs13167027 | 5 | 93071862 | T | C | 0.814 | 0.028 | 0.004 | 6.13E-12 | -0.002 | 0.010 | 0.820 |  |
| rs40465 | 5 | 103981726 | T | G | 0.676 | -0.032 | 0.003 | 1.57E-20 | -0.006 | 0.008 | 0.476 | Morningness, Number of sexual partners |
| rs288181 | 5 | 107349285 | C | T | 0.670 | 0.017 | 0.003 | 2.26E-08 | 0.013 | 0.007 | 0.087 |  |
| rs62379847 | 5 | 120109119 | A | C | 0.653 | -0.018 | 0.003 | 2.87E-09 | 0.010 | 0.007 | 0.159 |  |
| rs2408225 | 5 | 124262051 | T | C | 0.554 | 0.019 | 0.003 | 3.07E-11 | -0.008 | 0.007 | 0.272 |  |
| rs330340 | 5 | 124911548 | A | C | 0.279 | 0.018 | 0.003 | 1.30E-08 | -0.010 | 0.008 | 0.232 |  |
| rs3756334 | 5 | 140213796 | G | A | 0.482 | -0.017 | 0.003 | 1.03E-09 | 0.018 | 0.007 | 0.009 |  |
| rs2964003 | 5 | 153216733 | A | G | 0.822 | 0.024 | 0.004 | 3.21E-10 | -0.001 | 0.009 | 0.889 |  |
| rs2279020 | 5 | 161322889 | G | A | 0.355 | 0.018 | 0.003 | 1.02E-09 | 0.003 | 0.008 | 0.671 |  |
| rs4434398 | 5 | 164480866 | G | C | 0.462 | -0.029 | 0.003 | 5.98E-19 | 0.010 | 0.008 | 0.201 |  |
| rs4869058 | 5 | 166992247 | T | A | 0.351 | 0.018 | 0.003 | 6.16E-10 | 0.004 | 0.008 | 0.584 | Smoking status (ever vs never smokers) |
| rs12661596 | 6 | 17016400 | A | G | 0.771 | 0.019 | 0.003 | 4.94E-09 | 0.002 | 0.008 | 0.829 |  |
| rs35883476 | 6 | 28368508 | G | C | 0.916 | 0.047 | 0.005 | 1.42E-19 | -0.030 | 0.013 | 0.023 |  |
| rs115938232 | 6 | 29725013 | A | G | 0.946 | -0.046 | 0.006 | 1.14E-14 | 0.008 | 0.018 | 0.661 |  |
| rs10947690 | 6 | 37631768 | A | G | 0.755 | -0.020 | 0.003 | 3.15E-09 | -0.023 | 0.009 | 0.006 | Insomnia symptoms (never/rarely vs. sometimes/usually), Insomnia, Insomnia symptoms (never/rarely vs. usually) |
| rs537362 | 6 | 50650700 | G | C | 0.088 | 0.027 | 0.005 | 2.27E-08 | 0.019 | 0.014 | 0.179 |  |
| rs17752199 | 6 | 51406848 | A | G | 0.891 | 0.028 | 0.005 | 2.30E-09 | 0.013 | 0.011 | 0.247 | Glaucoma (primary open-angle), Glaucoma, Intraocular pressure |
| rs632024 | 6 | 64630933 | T | G | 0.298 | -0.020 | 0.003 | 1.91E-11 | -0.005 | 0.008 | 0.482 |  |
| rs4574611 | 6 | 66602204 | T | A | 0.623 | 0.021 | 0.003 | 7.83E-11 | 0.003 | 0.008 | 0.692 |  |
| rs1414592 | 6 | 76249698 | C | G | 0.492 | -0.017 | 0.003 | 2.69E-09 | 0.004 | 0.007 | 0.613 |  |
| rs12530388 | 6 | 101329173 | A | C | 0.488 | 0.020 | 0.003 | 4.34E-13 | -0.003 | 0.007 | 0.725 | Smoking initiation (ever regular vs never regular) |
| rs1933802 | 6 | 105365891 | C | G | 0.447 | -0.020 | 0.003 | 3.04E-12 | 0.005 | 0.007 | 0.520 |  |
| rs7752418 | 6 | 111787885 | C | T | 0.770 | -0.019 | 0.003 | 2.38E-08 | 0.003 | 0.008 | 0.764 |  |
| rs728017 | 6 | 124292594 | A | G | 0.401 | -0.019 | 0.003 | 7.81E-11 | -0.009 | 0.008 | 0.255 | Insomnia |
| rs12192791 | 6 | 130552266 | G | A | 0.709 | 0.018 | 0.003 | 9.66E-09 | 0.017 | 0.008 | 0.029 |  |
| rs2876520 | 6 | 142996618 | C | G | 0.534 | -0.018 | 0.003 | 4.88E-11 | 0.012 | 0.007 | 0.092 | Well-being spectrum |
| rs9479138 | 6 | 152215199 | T | G | 0.353 | 0.025 | 0.003 | 3.55E-18 | 0.004 | 0.008 | 0.616 |  |
| rs12190823 | 6 | 163092643 | G | A | 0.778 | -0.022 | 0.003 | 1.19E-10 | -0.010 | 0.008 | 0.256 |  |
| rs2322702 | 6 | 165178877 | G | A | 0.555 | 0.018 | 0.003 | 3.16E-10 | 0.011 | 0.007 | 0.144 |  |
| rs10268609 | 7 | 1962163 | G | T | 0.190 | -0.032 | 0.004 | 2.00E-18 | -0.021 | 0.009 | 0.026 |  |
| rs957360 | 7 | 3660918 | C | G | 0.705 | 0.019 | 0.003 | 6.64E-10 | 0.004 | 0.008 | 0.582 |  |
| rs6460905 | 7 | 12283227 | A | G | 0.595 | -0.023 | 0.003 | 3.04E-16 | -0.029 | 0.007 | 4.63E-05 |  |
| rs66974425 | 7 | 13954631 | C | T | 0.751 | 0.018 | 0.003 | 1.64E-08 | 0.002 | 0.008 | 0.836 |  |
| rs7799431 | 7 | 32828281 | G | A | 0.642 | 0.019 | 0.003 | 2.98E-10 | 0.001 | 0.008 | 0.926 |  |
| rs57389877 | 7 | 68412422 | G | A | 0.567 | -0.016 | 0.003 | 1.11E-08 | -0.008 | 0.007 | 0.242 |  |
| rs12707530 | 7 | 82497972 | G | A | 0.660 | -0.020 | 0.003 | 5.12E-11 | -0.001 | 0.007 | 0.926 |  |
| rs9690219 | 7 | 109106510 | C | T | 0.425 | -0.019 | 0.003 | 8.77E-12 | -0.005 | 0.007 | 0.505 |  |
| rs8180817 | 7 | 114047542 | G | C | 0.554 | 0.019 | 0.003 | 2.57E-11 | 0.010 | 0.007 | 0.168 | Insomnia |
| rs1548461 | 7 | 117567576 | T | C | 0.479 | -0.019 | 0.003 | 1.78E-11 | -0.016 | 0.007 | 0.019 |  |
| rs1986692 | 7 | 133743393 | A | G | 0.611 | 0.017 | 0.003 | 2.31E-09 | 0.001 | 0.007 | 0.858 |  |
| rs3812281 | 7 | 135082751 | T | C | 0.605 | -0.017 | 0.003 | 1.49E-09 | -0.008 | 0.007 | 0.268 | Educational attainment (years of education) |
| rs34269011 | 8 | 14220114 | C | T | 0.726 | 0.019 | 0.003 | 3.50E-10 | 0.000 | 0.008 | 0.961 |  |
| rs7017108 | 8 | 20772332 | C | T | 0.570 | -0.018 | 0.003 | 2.28E-10 | -0.004 | 0.007 | 0.615 |  |
| rs6471757 | 8 | 59805738 | G | A | 0.395 | 0.019 | 0.003 | 4.57E-11 | -0.011 | 0.008 | 0.170 |  |
| rs1425794 | 8 | 64806567 | G | T | 0.551 | -0.016 | 0.003 | 6.46E-09 | 0.005 | 0.007 | 0.467 |  |
| rs7837935 | 8 | 65562019 | T | G | 0.145 | -0.030 | 0.004 | 1.33E-14 | -0.002 | 0.010 | 0.813 | Neurociticism |
| rs1433965 | 8 | 77633344 | T | C | 0.836 | -0.022 | 0.004 | 5.12E-09 | -0.006 | 0.009 | 0.522 |  |
| rs4478545 | 8 | 94672542 | G | A | 0.712 | 0.018 | 0.003 | 2.93E-09 | 0.007 | 0.008 | 0.353 |  |
| rs6470670 | 8 | 129913448 | T | C | 0.299 | -0.020 | 0.003 | 2.57E-10 | -0.013 | 0.008 | 0.098 |  |
| rs10441718 | 9 | 2989299 | C | T | 0.402 | -0.018 | 0.003 | 1.57E-10 | -0.011 | 0.007 | 0.124 |  |
| rs1931388 | 9 | 11203149 | A | G | 0.594 | 0.024 | 0.003 | 4.36E-16 | 0.005 | 0.007 | 0.456 |  |
| rs6474807 | 9 | 14061876 | G | C | 0.290 | -0.017 | 0.003 | 4.46E-08 | -0.013 | 0.009 | 0.113 |  |
| rs12003380 | 9 | 17135434 | G | C | 0.562 | 0.017 | 0.003 | 6.68E-09 | 0.007 | 0.007 | 0.371 |  |
| rs3793577 | 9 | 23737627 | A | G | 0.469 | -0.022 | 0.003 | 2.07E-15 | 0.000 | 0.008 | 0.995 | Well-being spectrum, Neuroticism, Positive affect, Life satisfaction |
| rs4531131 | 9 | 26723702 | A | G | 0.819 | 0.022 | 0.004 | 3.41E-08 | 0.017 | 0.009 | 0.046 |  |
| rs13296641 | 9 | 31160243 | G | A | 0.682 | 0.021 | 0.003 | 1.49E-12 | 0.005 | 0.008 | 0.549 |  |
| rs1329572 | 9 | 37001471 | T | A | 0.600 | -0.025 | 0.003 | 1.41E-17 | -0.013 | 0.007 | 0.070 | Well-being spectrum |
| rs11138500 | 9 | 82737534 | G | A | 0.295 | -0.018 | 0.003 | 3.21E-08 | 0.007 | 0.009 | 0.445 |  |
| rs7023933 | 9 | 96225478 | A | T | 0.834 | -0.026 | 0.004 | 2.31E-12 | -0.010 | 0.010 | 0.279 | Worry |
| rs182923402 | 9 | 98299677 | T | A | 0.915 | -0.030 | 0.005 | 4.73E-09 | -0.021 | 0.012 | 0.080 |  |
| rs2418449 | 9 | 119731359 | T | C | 0.709 | 0.022 | 0.003 | 1.15E-12 | -0.002 | 0.008 | 0.768 |  |
| rs913930 | 9 | 120484009 | G | A | 0.359 | 0.018 | 0.003 | 1.41E-09 | -0.004 | 0.007 | 0.570 | Neuroticism |
| rs12553064 | 9 | 122660054 | C | A | 0.390 | -0.020 | 0.003 | 3.36E-12 | -0.005 | 0.008 | 0.506 | Educational attainment |
| rs667138 | 9 | 126658613 | A | C | 0.852 | -0.025 | 0.004 | 4.23E-09 | 0.004 | 0.011 | 0.721 |  |
| rs10858298 | 9 | 137839527 | G | A | 0.299 | 0.018 | 0.003 | 1.02E-08 | 0.002 | 0.008 | 0.766 |  |
| rs112535944 | 9 | 140278206 | C | A | 0.841 | -0.045 | 0.007 | 1.77E-10 | -0.025 | 0.013 | 0.045 |  |
| rs4880918 | 10 | 1780944 | C | G | 0.484 | 0.017 | 0.003 | 1.98E-09 | 0.017 | 0.008 | 0.022 |  |
| rs1993849 | 10 | 67796008 | G | A | 0.194 | 0.022 | 0.004 | 5.72E-10 | -0.001 | 0.009 | 0.907 |  |
| rs4515864 | 10 | 68530073 | G | A | 0.201 | 0.020 | 0.004 | 9.08E-09 | 0.010 | 0.009 | 0.291 |  |
| rs3088142 | 10 | 76854564 | C | T | 0.575 | -0.018 | 0.003 | 4.92E-11 | -0.004 | 0.007 | 0.586 | Body mass index |
| rs1909696 | 10 | 77582203 | G | T | 0.330 | -0.018 | 0.003 | 2.77E-09 | -0.004 | 0.008 | 0.568 |  |
| rs61691222 | 10 | 104420754 | C | T | 0.774 | -0.019 | 0.003 | 3.72E-09 | 0.010 | 0.009 | 0.246 |  |
| rs17186548 | 10 | 106550944 | G | A | 0.788 | 0.020 | 0.003 | 9.13E-09 | -0.009 | 0.009 | 0.297 |  |
| rs1021363 | 10 | 106610839 | A | G | 0.339 | 0.027 | 0.003 | 5.32E-20 | -0.003 | 0.008 | 0.685 |  |
| rs111442587 | 10 | 114984089 | C | G | 0.976 | -0.053 | 0.009 | 1.54E-08 | -0.040 | 0.025 | 0.110 |  |
| rs10749240 | 10 | 118914837 | T | C | 0.596 | -0.016 | 0.003 | 4.96E-08 | 0.004 | 0.007 | 0.609 |  |
| rs12285422 | 11 | 24591463 | G | C | 0.627 | 0.019 | 0.003 | 1.04E-08 | 0.009 | 0.007 | 0.214 |  |
| rs11030380 | 11 | 28591168 | A | T | 0.399 | -0.022 | 0.003 | 1.02E-14 | -0.007 | 0.007 | 0.328 | Alcohol consumption (drinks per week) |
| rs324300 | 11 | 29674831 | G | A | 0.619 | -0.017 | 0.003 | 1.80E-09 | 0.001 | 0.007 | 0.892 |  |
| rs3026389 | 11 | 31813529 | C | G | 0.205 | 0.023 | 0.003 | 4.90E-12 | -0.003 | 0.009 | 0.758 | Neurociticism, Feeling worry |
| rs80067584 | 11 | 32597748 | C | A | 0.947 | 0.038 | 0.006 | 1.10E-09 | -0.003 | 0.016 | 0.836 |  |
| rs1825119 | 11 | 38556991 | A | G | 0.646 | -0.018 | 0.003 | 1.15E-09 | 0.002 | 0.007 | 0.765 |  |
| rs112181005 | 11 | 47010491 | G | A | 0.855 | 0.025 | 0.004 | 1.51E-09 | -0.022 | 0.010 | 0.028 |  |
| rs2509805 | 11 | 57650796 | T | C | 0.328 | 0.023 | 0.003 | 5.31E-14 | 0.017 | 0.008 | 0.025 |  |
| rs102275 | 11 | 61557803 | T | C | 0.640 | -0.020 | 0.003 | 1.96E-12 | 0.015 | 0.007 | 0.035 | Serum metabolite ratios in chronic kidney disease, Plasma omega-6 polyunsaturated fatty acid levels (arachidonic acid), Plasma omega-3 polyunsaturated fatty acid levels (docosapentaenoic acid), Oleic acid (18:1n-9) levels, Glycerophospholipid levels, Trans fatty acid levels, Serum metabolite concentrations in chronic kidney disease, Metabolic syndrome, Red blood cell count, Plasma omega-3 polyunsaturated fatty acid levels (alphalinolenic acid), Palmitoleic acid (16:1n-7) levels, Irritable mood, Stearic acid (18:0) levels, Phospholipid levels (plasma), Crohn's disease, Metabolite levels |
| rs11600628 | 11 | 63976027 | C | T | 0.845 | 0.022 | 0.004 | 1.25E-08 | 0.018 | 0.010 | 0.076 |  |
| rs12789028 | 11 | 65326154 | G | A | 0.825 | -0.022 | 0.004 | 8.96E-10 | -0.023 | 0.010 | 0.014 |  |
| rs7117514 | 11 | 70544937 | A | G | 0.547 | -0.015 | 0.003 | 4.51E-08 | -0.007 | 0.007 | 0.345 |  |
| rs4753209 | 11 | 88743208 | T | A | 0.439 | 0.024 | 0.003 | 9.57E-16 | 0.013 | 0.008 | 0.106 |  |
| rs4937872 | 11 | 112827715 | A | G | 0.533 | -0.016 | 0.003 | 1.53E-08 | -0.014 | 0.009 | 0.109 | Feeling tense, Worry |
| rs61902811 | 11 | 113370758 | G | A | 0.665 | 0.026 | 0.003 | 1.04E-18 | 0.012 | 0.008 | 0.105 |  |
| rs7130926 | 11 | 113496928 | T | C | 0.863 | -0.027 | 0.004 | 1.10E-10 | -0.032 | 0.011 | 0.004 |  |
| rs7101595 | 11 | 126629031 | C | T | 0.437 | -0.016 | 0.003 | 1.74E-08 | -0.002 | 0.007 | 0.765 |  |
| rs57344483 | 11 | 127022560 | A | G | 0.915 | -0.031 | 0.005 | 1.30E-08 | 0.004 | 0.013 | 0.742 |  |
| rs612823 | 11 | 133834104 | T | C | 0.591 | 0.021 | 0.003 | 1.07E-12 | 0.010 | 0.008 | 0.212 |  |
| rs11062170 | 12 | 2348844 | G | C | 0.675 | -0.017 | 0.003 | 9.56E-09 | -0.016 | 0.007 | 0.034 |  |
| rs10491990 | 12 | 11939528 | G | A | 0.838 | 0.022 | 0.004 | 2.75E-08 | 0.003 | 0.010 | 0.779 |  |
| rs7968921 | 12 | 23960177 | T | C | 0.405 | -0.018 | 0.003 | 3.99E-10 | -0.015 | 0.007 | 0.043 |  |
| rs16926971 | 12 | 24169797 | T | C | 0.792 | 0.021 | 0.004 | 2.30E-09 | 0.004 | 0.009 | 0.662 |  |
| rs11612312 | 12 | 52349088 | T | C | 0.798 | -0.021 | 0.004 | 8.87E-10 | 0.018 | 0.009 | 0.037 | Positive affect |
| rs73123350 | 12 | 58050250 | G | T | 0.579 | -0.016 | 0.003 | 1.91E-08 | -0.015 | 0.007 | 0.042 |  |
| rs56051015 | 12 | 84497937 | C | G | 0.811 | -0.022 | 0.004 | 2.24E-10 | -0.002 | 0.009 | 0.790 |  |
| rs2292996 | 12 | 103556972 | T | C | 0.483 | -0.016 | 0.003 | 1.66E-08 | 0.000 | 0.007 | 0.998 |  |
| rs521048 | 12 | 118830958 | A | G | 0.265 | -0.018 | 0.003 | 2.98E-08 | -0.002 | 0.009 | 0.827 |  |
| rs3213572 | 12 | 121205078 | G | A | 0.530 | -0.021 | 0.003 | 1.02E-13 | 0.006 | 0.007 | 0.412 |  |
| rs7334101 | 13 | 31900245 | G | A | 0.217 | -0.021 | 0.003 | 9.42E-10 | -0.004 | 0.009 | 0.639 |  |
| rs9536383 | 13 | 53865563 | C | T | 0.686 | -0.024 | 0.003 | 9.92E-15 | 0.015 | 0.008 | 0.049 |  |
| rs7988498 | 13 | 66946477 | C | T | 0.503 | 0.019 | 0.003 | 1.88E-11 | 0.000 | 0.007 | 0.954 |  |
| rs7982263 | 13 | 80061074 | T | C | 0.404 | -0.015 | 0.003 | 4.30E-08 | -0.004 | 0.007 | 0.615 |  |
| rs508502 | 13 | 80921519 | C | T | 0.697 | 0.020 | 0.003 | 3.06E-10 | 0.001 | 0.008 | 0.894 |  |
| rs73532287 | 13 | 85234525 | G | T | 0.800 | -0.020 | 0.004 | 2.12E-08 | -0.010 | 0.009 | 0.290 |  |
| rs9561328 | 13 | 94009312 | G | T | 0.873 | -0.025 | 0.004 | 1.51E-09 | -0.015 | 0.011 | 0.154 |  |
| rs7317645 | 13 | 97130674 | T | C | 0.162 | -0.024 | 0.004 | 1.35E-09 | -0.017 | 0.010 | 0.086 |  |
| rs4772087 | 13 | 99115041 | C | T | 0.620 | -0.020 | 0.003 | 8.25E-12 | 0.005 | 0.008 | 0.494 |  |
| rs11157241 | 14 | 42051771 | T | C | 0.506 | 0.023 | 0.003 | 2.12E-16 | 0.011 | 0.007 | 0.131 |  |
| rs2274794 | 14 | 57283934 | C | T | 0.666 | 0.021 | 0.003 | 8.09E-12 | 0.012 | 0.008 | 0.122 |  |
| rs10148598 | 14 | 60147659 | C | T | 0.241 | 0.019 | 0.003 | 1.56E-09 | 0.008 | 0.009 | 0.380 |  |
| rs10142766 | 14 | 64780454 | A | G | 0.445 | -0.019 | 0.003 | 2.77E-11 | 0.009 | 0.007 | 0.203 |  |
| rs11621186 | 14 | 75120628 | C | T | 0.500 | -0.022 | 0.003 | 1.45E-15 | -0.012 | 0.007 | 0.089 |  |
| rs3008497 | 14 | 85038175 | G | T | 0.682 | 0.016 | 0.003 | 4.57E-08 | 0.004 | 0.008 | 0.555 |  |
| rs1286060 | 14 | 91458156 | G | A | 0.250 | -0.018 | 0.003 | 4.34E-08 | 0.023 | 0.008 | 0.005 |  |
| rs7141014 | 14 | 98667928 | T | C | 0.803 | -0.021 | 0.004 | 3.00E-09 | -0.003 | 0.009 | 0.735 |  |
| rs9671376 | 14 | 103370163 | C | T | 0.763 | -0.020 | 0.003 | 2.99E-09 | -0.018 | 0.008 | 0.028 |  |
| rs10149470 | 14 | 104017953 | A | G | 0.485 | -0.023 | 0.003 | 1.96E-15 | 0.010 | 0.007 | 0.162 | Extremely high intelligence, Autism and major depressive disorder, Cognitive ability, Intelligence |
| rs12898464 | 15 | 29853387 | C | T | 0.118 | 0.025 | 0.005 | 4.26E-08 | 0.004 | 0.013 | 0.750 |  |
| rs11073153 | 15 | 36425448 | G | T | 0.403 | -0.016 | 0.003 | 4.94E-08 | 0.003 | 0.008 | 0.709 |  |
| rs8037355 | 15 | 37643831 | C | T | 0.462 | 0.018 | 0.003 | 1.07E-10 | -0.010 | 0.007 | 0.144 |  |
| rs56059718 | 15 | 38836777 | C | A | 0.806 | -0.021 | 0.004 | 2.67E-09 | 0.002 | 0.009 | 0.793 |  |
| rs34488670 | 15 | 47684936 | T | C | 0.785 | -0.024 | 0.003 | 2.40E-12 | 0.005 | 0.009 | 0.561 | Age of smoking initiation, Risk-taking tendency (4-domain principal component model), Number of sexual partners |
| rs8040348 | 15 | 47999319 | C | T | 0.615 | 0.016 | 0.003 | 2.76E-08 | 0.001 | 0.008 | 0.905 |  |
| rs79714693 | 15 | 63993463 | C | T | 0.794 | -0.023 | 0.004 | 4.81E-11 | -0.016 | 0.009 | 0.058 |  |
| rs4776729 | 15 | 66399197 | A | G | 0.869 | -0.026 | 0.004 | 4.13E-10 | -0.002 | 0.011 | 0.828 |  |
| rs2060337 | 15 | 70615407 | T | G | 0.472 | 0.018 | 0.003 | 6.36E-10 | 0.012 | 0.008 | 0.099 | Highest math class taken, Self-reported math ability |
| rs8030745 | 15 | 74133091 | T | C | 0.879 | 0.029 | 0.004 | 1.53E-11 | 0.011 | 0.011 | 0.306 |  |
| rs12900865 | 15 | 78075910 | G | A | 0.425 | -0.017 | 0.003 | 1.43E-09 | -0.005 | 0.007 | 0.468 |  |
| rs11855600 | 15 | 88963259 | C | T | 0.828 | 0.021 | 0.004 | 2.55E-08 | 0.006 | 0.010 | 0.574 |  |
| rs4702 | 15 | 91426560 | G | A | 0.452 | 0.020 | 0.003 | 1.66E-12 | -0.019 | 0.008 | 0.012 | Insomnia, Autism spectrum disorder or schizophrenia, General risk tolerance, Number of sexual partners, Age at first sexual intercourse, Schizophrenia, Feeling hurt |
| rs7193263 | 16 | 6315880 | G | A | 0.340 | 0.020 | 0.003 | 3.51E-11 | 0.005 | 0.008 | 0.531 |  |
| rs11077206 | 16 | 7667332 | G | C | 0.373 | -0.016 | 0.003 | 2.63E-08 | -0.006 | 0.008 | 0.408 |  |
| rs7200826 | 16 | 13066833 | C | T | 0.730 | -0.024 | 0.003 | 1.07E-13 | 0.005 | 0.008 | 0.548 | Well-being spectrum, Neuroticism |
| rs7184632 | 16 | 13750997 | C | G | 0.284 | 0.027 | 0.004 | 4.88E-14 | -0.003 | 0.008 | 0.692 |  |
| rs2369818 | 16 | 21614009 | C | T | 0.574 | -0.021 | 0.003 | 3.25E-13 | 0.007 | 0.008 | 0.386 |  |
| rs4785307 | 16 | 49494029 | A | G | 0.426 | 0.018 | 0.003 | 8.54E-10 | -0.006 | 0.008 | 0.401 |  |
| rs4784296 | 16 | 51479349 | G | A | 0.532 | 0.015 | 0.003 | 3.94E-08 | -0.002 | 0.007 | 0.760 |  |
| rs11643192 | 16 | 72214276 | C | A | 0.594 | -0.017 | 0.003 | 8.51E-09 | -0.006 | 0.007 | 0.402 | Ease of getting up in the morning |
| rs11644513 | 16 | 82868852 | C | T | 0.662 | 0.017 | 0.003 | 1.44E-08 | 0.003 | 0.008 | 0.721 |  |
| rs2585399 | 17 | 8054759 | G | A | 0.395 | -0.017 | 0.003 | 1.21E-08 | -0.005 | 0.007 | 0.506 |  |
| rs75581564 | 17 | 27363750 | G | A | 0.883 | -0.025 | 0.004 | 1.06E-08 | -0.006 | 0.011 | 0.598 |  |
| rs412891 | 17 | 31545583 | G | T | 0.264 | -0.020 | 0.003 | 1.93E-10 | 0.023 | 0.008 | 0.005 |  |
| rs60856912 | 17 | 65892343 | G | T | 0.819 | -0.026 | 0.004 | 1.61E-11 | -0.016 | 0.009 | 0.080 | Endometrial cancer, Smoking initiation (ever regular vs never regular) |
| rs10460051 | 18 | 31413679 | C | T | 0.491 | -0.019 | 0.003 | 4.19E-12 | -0.002 | 0.007 | 0.830 | Neurociticism, Neuroticism |
| rs12967855 | 18 | 35138245 | A | G | 0.321 | 0.026 | 0.003 | 2.86E-18 | 0.001 | 0.008 | 0.910 | Educational attainment (years of education), Depressed affect |
| rs73428490 | 18 | 36614279 | C | A | 0.913 | 0.028 | 0.005 | 7.47E-09 | 0.019 | 0.012 | 0.116 |  |
| rs4267411 | 18 | 39305256 | C | T | 0.821 | 0.022 | 0.004 | 4.84E-10 | 0.009 | 0.009 | 0.356 |  |
| rs7230285 | 18 | 50723080 | G | A | 0.499 | -0.024 | 0.003 | 1.17E-17 | -0.001 | 0.007 | 0.891 |  |
| rs1819881 | 18 | 52471573 | C | G | 0.209 | -0.022 | 0.004 | 1.08E-09 | 0.004 | 0.009 | 0.696 |  |
| rs4131791 | 18 | 52747871 | C | T | 0.587 | 0.021 | 0.003 | 4.80E-14 | 0.005 | 0.007 | 0.500 |  |
| rs12967143 | 18 | 53099012 | G | C | 0.294 | 0.025 | 0.003 | 5.94E-16 | -0.008 | 0.008 | 0.308 | Well-being spectrum |
| rs7241572 | 18 | 77580712 | G | A | 0.797 | -0.026 | 0.004 | 1.31E-11 | -0.004 | 0.010 | 0.682 |  |
| rs1555133 | 20 | 31048382 | G | A | 0.656 | -0.017 | 0.003 | 9.91E-09 | 0.005 | 0.008 | 0.536 | Monocyte count |
| rs6131010 | 20 | 44724305 | A | G | 0.268 | 0.024 | 0.003 | 9.85E-14 | 0.020 | 0.008 | 0.015 | Well-being spectrum |
| rs910187 | 20 | 45841052 | G | A | 0.638 | 0.020 | 0.003 | 6.25E-11 | -0.009 | 0.008 | 0.231 | Insomnia, Neuroticism, Migraine, Well-being spectrum |
| rs2273653 | 20 | 47770756 | A | C | 0.587 | 0.018 | 0.003 | 2.48E-10 | 0.012 | 0.007 | 0.084 | Well-being spectrum |
| rs520838 | 20 | 59002955 | C | A | 0.756 | -0.018 | 0.003 | 2.95E-08 | 0.014 | 0.008 | 0.090 |  |
| rs9974713 | 21 | 38817699 | T | C | 0.233 | -0.019 | 0.004 | 3.85E-08 | -0.002 | 0.009 | 0.806 |  |
| rs2838788 | 21 | 46542374 | T | C | 0.612 | 0.016 | 0.003 | 2.09E-08 | -0.001 | 0.007 | 0.845 |  |
| rs2273085 | 22 | 41615376 | C | T | 0.710 | -0.025 | 0.003 | 2.85E-16 | -0.006 | 0.008 | 0.430 | Neuroticism |
| rs9627391 | 22 | 46447097 | C | T | 0.683 | 0.017 | 0.003 | 1.28E-08 | -0.004 | 0.008 | 0.643 |  |
| SNP, single nucleotide polymorphisms; CHR, chromosome; BP, physical position of SNP (base-pairs); A1, effect allele; A2, alternative allele; EAF: effect allele frequency; Beta, effect allele beta coefficient; SE, standard error of the beta coefficient. | | | | | | | | | | | | |

| **Supplementary Table 3. Characteristics of genetic instruments of stroke and their effect sizes with depression.** | | | | | | | | | | | | |
| --- | --- | --- | --- | --- | --- | --- | --- | --- | --- | --- | --- | --- |
| SNP | CHR | BP | A1 | A2 | EAF | Exposure | | | Outcome | | | Pleiotropic traits |
|  |  |  |  |  |  | Beta | SE | P | Beta | SE | P |  |
| rs2455132 | 1 | 3221083 | T | C | 0.240 | -0.045 | 0.008 | 3.44E-08 | -0.006 | 0.004 | 0.117 |  |
| rs880315 | 1 | 10796866 | T | C | 0.633 | -0.044 | 0.007 | 2.89E-10 | -0.006 | 0.003 | 0.060 | Diastolic blood pressure, Urinary albumin-to-creatinine ratio, Mean arterial pressure, Systolic blood pressure, Blood pressure, Medication use (calcium channel blockers), Pulse pressure, Cardiovascular disease, Systolic blood pressure (cigarette smoking interaction), Hypertension, Medication use (agents acting on the renin-angiotensin system), Diastolic blood pressure (cigarette smoking interaction), Medication use (diuretics), Atrial fibrillation |
| rs2251636 | 1 | 156202809 | C | G | 0.373 | -0.044 | 0.007 | 4.09E-11 | -0.003 | 0.003 | 0.373 |  |
| rs2351524 | 2 | 203880992 | T | C | 0.124 | -0.063 | 0.010 | 5.40E-10 | -0.008 | 0.005 | 0.107 |  |
| rs6847935 | 4 | 111696651 | A | T | 0.785 | -0.072 | 0.008 | 6.15E-20 | -0.003 | 0.004 | 0.548 | Atrial fibrillation |
| rs6536024 | 4 | 155543369 | T | C | 0.480 | -0.049 | 0.007 | 8.62E-14 | -0.002 | 0.003 | 0.469 | Venous thromboembolism |
| rs17148926 | 5 | 121510586 | A | C | 0.824 | 0.060 | 0.009 | 1.02E-11 | -0.006 | 0.004 | 0.147 | White matter hyperintensity burden |
| rs79318212 | 6 | 1365244 | A | G | 0.885 | -0.078 | 0.011 | 2.79E-13 | 0.002 | 0.005 | 0.644 |  |
| rs2501966 | 6 | 49457835 | A | G | 0.595 | 0.038 | 0.007 | 5.12E-09 | 0.005 | 0.003 | 0.154 |  |
| rs42035 | 7 | 92239531 | A | G | 0.755 | 0.048 | 0.008 | 2.64E-10 | 0.004 | 0.004 | 0.248 |  |
| rs12539561 | 7 | 106437611 | T | C | 0.825 | -0.045 | 0.009 | 4.42E-07 | 0.001 | 0.005 | 0.895 |  |
| rs1549758 | 7 | 150695726 | T | C | 0.302 | 0.045 | 0.008 | 4.46E-09 | 0.006 | 0.004 | 0.074 |  |
| rs7859362 | 9 | 22105927 | T | C | 0.510 | -0.046 | 0.006 | 4.36E-13 | -0.007 | 0.003 | 0.019 |  |
| rs649129 | 9 | 136154304 | T | C | 0.223 | 0.051 | 0.009 | 4.11E-08 | -0.006 | 0.004 | 0.141 | Total cholesterol levels, Low density lipoprotein cholesterol levels, Blood metabolite ratios, Soluble levels of adhesion molecules |
| rs55983834 | 10 | 105601210 | T | C | 0.347 | -0.048 | 0.008 | 4.02E-09 | -0.002 | 0.003 | 0.469 |  |
| rs10886430 | 10 | 121010256 | A | G | 0.876 | -0.069 | 0.011 | 9.31E-11 | -0.008 | 0.005 | 0.108 | Platelet distribution width, Mean platelet volume |
| rs60401382 | 10 | 124227624 | T | C | 0.242 | -0.035 | 0.008 | 4.16E-06 | 0.006 | 0.004 | 0.094 |  |
| rs72985562 | 11 | 102800278 | T | G | 0.902 | -0.051 | 0.011 | 7.37E-06 | -0.006 | 0.006 | 0.282 | Post bronchodilator FEV1/FVC ratio |
| rs10774625 | 12 | 111910219 | A | G | 0.480 | 0.055 | 0.007 | 6.23E-17 | -0.002 | 0.003 | 0.623 | Estimated glomerular filtration rate, Systemic lupus erythematosus, Retinal vascular caliber, Coronary artery disease, Left ventricle diastolic internal dimension, Asthma (childhood onset), Glycated hemoglobin levels, Asthma (age of onset), Hypothyroidism |
| rs9515201 | 13 | 111040798 | A | C | 0.299 | 0.037 | 0.007 | 6.05E-07 | 0.004 | 0.004 | 0.225 | White matter hyperintensity burden |
| rs12445022 | 16 | 87575332 | A | G | 0.330 | 0.044 | 0.007 | 5.65E-10 | -0.001 | 0.003 | 0.854 | Systemic juvenile idiopathic arthritis |
| rs2108911 | 17 | 56570500 | T | C | 0.803 | -0.045 | 0.008 | 8.15E-08 | 0.005 | 0.005 | 0.252 |  |
| rs11907011 | 20 | 33767770 | T | C | 0.110 | -0.063 | 0.010 | 1.15E-09 | -0.011 | 0.005 | 0.044 | Factor VII activity |
| SNP, single nucleotide polymorphisms; CHR, chromosome; BP, physical position of SNP (base-pairs); A1, effect allele; A2, alternative allele; EAF: effect allele frequency; Beta, effect allele beta coefficient; SE, standard error of the beta coefficient. | | | | | | | | | | | | |

| **Supplementary Table 4. Characteristics of genetic instruments of ischemic stroke and their effect sizes with depression.** | | | | | | | | | | | | |
| --- | --- | --- | --- | --- | --- | --- | --- | --- | --- | --- | --- | --- |
| SNP | CHR | BP | A1 | A2 | EAF | Exposure | | | Outcome | | | Pleiotropic traits |
|  |  |  |  |  |  | Beta | SE | P | Beta | SE | P |  |
| rs880315 | 1 | 10796866 | T | C | 0.632 | -0.045 | 0.008 | 2.00E-09 | -0.006 | 0.003 | 0.060 | Diastolic blood pressure, Urinary albumin-to-creatinine ratio, Mean arterial pressure, Systolic blood pressure, Blood pressure, Medication use (calcium channel blockers), Pulse pressure, Cardiovascular disease, Systolic blood pressure (cigarette smoking interaction), Hypertension, Medication use (agents acting on the renin-angiotensin system), Diastolic blood pressure (cigarette smoking interaction), Medication use (diuretics), Atrial fibrillation |
| rs2251636 | 1 | 156202809 | C | G | 0.373 | -0.046 | 0.007 | 1.39E-10 | -0.003 | 0.003 | 0.373 |  |
| rs2351524 | 2 | 203880992 | T | C | 0.124 | -0.069 | 0.011 | 3.37E-10 | -0.008 | 0.005 | 0.107 |  |
| rs6847935 | 4 | 111696651 | A | T | 0.785 | -0.077 | 0.008 | 7.18E-20 | -0.003 | 0.004 | 0.548 | Atrial fibrillation |
| rs6536024 | 4 | 155543369 | T | C | 0.480 | -0.054 | 0.007 | 1.36E-14 | -0.002 | 0.003 | 0.469 | Venous thromboembolism |
| rs4444878 | 4 | 187213883 | A | C | 0.409 | 0.043 | 0.007 | 1.71E-09 | 0.004 | 0.003 | 0.191 | Thrombosis |
| rs17148926 | 5 | 121510586 | A | C | 0.824 | 0.061 | 0.010 | 2.98E-10 | -0.006 | 0.004 | 0.147 | White matter hyperintensity burden |
| rs79318212 | 6 | 1365244 | A | G | 0.885 | -0.086 | 0.012 | 5.40E-14 | 0.002 | 0.005 | 0.644 |  |
| rs36229526 | 6 | 32820656 | T | G | 0.082 | 0.074 | 0.014 | 4.77E-08 | 0.003 | 0.006 | 0.581 |  |
| rs42035 | 7 | 92239531 | A | G | 0.755 | 0.055 | 0.008 | 3.76E-11 | 0.004 | 0.004 | 0.248 |  |
| rs1549758 | 7 | 150695726 | T | C | 0.302 | 0.048 | 0.008 | 8.97E-09 | 0.006 | 0.004 | 0.074 |  |
| rs1487504 | 9 | 17000855 | A | G | 0.112 | 0.062 | 0.011 | 4.66E-08 | -0.012 | 0.005 | 0.016 |  |
| rs7859362 | 9 | 22105927 | T | C | 0.509 | -0.046 | 0.007 | 4.38E-11 | -0.007 | 0.003 | 0.019 |  |
| rs649129 | 9 | 136154304 | T | C | 0.223 | 0.062 | 0.010 | 1.04E-09 | -0.006 | 0.004 | 0.141 | Total cholesterol levels, Low density lipoprotein cholesterol levels, Blood metabolite ratios, Soluble levels of adhesion molecules |
| rs10886430 | 10 | 121010256 | A | G | 0.876 | -0.078 | 0.011 | 1.07E-11 | -0.008 | 0.005 | 0.108 | Platelet distribution width, Mean platelet volume |
| rs60401382 | 10 | 124227624 | T | C | 0.243 | -0.037 | 0.008 | 6.65E-06 | 0.006 | 0.004 | 0.094 |  |
| rs72985562 | 11 | 102800278 | T | G | 0.901 | -0.061 | 0.012 | 4.54E-07 | -0.006 | 0.006 | 0.282 | Post bronchodilator FEV1/FVC ratio |
| rs7304841 | 12 | 20577593 | A | C | 0.593 | 0.040 | 0.007 | 3.38E-08 | 0.002 | 0.003 | 0.538 |  |
| rs10774625 | 12 | 111910219 | A | G | 0.479 | 0.065 | 0.007 | 1.22E-19 | -0.002 | 0.003 | 0.623 | Estimated glomerular filtration rate, Systemic lupus erythematosus, Retinal vascular caliber, Coronary artery disease, Left ventricle diastolic internal dimension, Asthma (childhood onset), Glycated hemoglobin levels, Asthma (age of onset), Hypothyroidism |
| rs7974266 | 12 | 113007602 | T | C | 0.701 | 0.048 | 0.008 | 3.36E-09 | -0.005 | 0.004 | 0.178 | Systolic blood pressure x smoking status (ever vs never) interaction (2df test), Diastolic blood pressure x smoking status (current vs non-current) interaction (2df test), Diastolic blood pressure x smoking status (ever vs never) interaction (2df test), Systolic blood pressure x smoking status (current vs non-current) interaction (2df test) |
| rs842365 | 13 | 47252379 | A | G | 0.730 | 0.045 | 0.008 | 3.56E-08 | -0.004 | 0.004 | 0.249 |  |
| rs1573644 | 15 | 91421283 | T | C | 0.658 | -0.046 | 0.008 | 6.60E-09 | 0.009 | 0.004 | 0.012 |  |
| rs2397816 | 15 | 96119880 | A | G | 0.367 | 0.041 | 0.008 | 2.61E-07 | 0.001 | 0.003 | 0.824 |  |
| rs12445022 | 16 | 87575332 | A | G | 0.329 | 0.050 | 0.008 | 9.14E-11 | -0.001 | 0.003 | 0.854 | Systemic juvenile idiopathic arthritis |
| rs2108911 | 17 | 56570500 | T | C | 0.802 | -0.052 | 0.009 | 9.18E-09 | 0.005 | 0.005 | 0.252 |  |
| rs8106503 | 19 | 11196886 | T | C | 0.890 | 0.065 | 0.011 | 7.85E-09 | -0.002 | 0.005 | 0.681 | Total cholesterol levels |
| rs11907011 | 20 | 33767770 | T | C | 0.111 | -0.071 | 0.011 | 2.54E-10 | -0.011 | 0.005 | 0.044 | Factor VII activity |
| SNP, single nucleotide polymorphisms; CHR, chromosome; BP, physical position of SNP (base-pairs); A1, effect allele; A2, alternative allele; EAF: effect allele frequency; Beta, effect allele beta coefficient; SE, standard error of the beta coefficient. | | | | | | | | | | | | |

| **Supplementary Table 5. Local heritability of depression and stroke, and regions with significant local genetic covariance estimated by SUPERGNOVA.** | | | | | | | |
| --- | --- | --- | --- | --- | --- | --- | --- |
| Locus | Number of SNPs† | Trait-pairs | Local h^2^_depression_ | Local h^2^_stroke_ | Local genetic correlation‡ | Local genetic covariance* | P |
| 11: 113105405-113958177 | 414 | Depression and stroke | 2.47×10^-4^ | 2.29×10^-5^ | 0.80 | 5.98×10^-5^ (1.76×10^-10^) | 6.45×10^-6^ |
| 11: 113105405-113958177 | 414 | Depression and ischemic stroke | 2.47×10^-4^ | 2.05×10^-5^ | 0.82 | 5.86×10^-5^ (1.55×10^-10^) | 2.55×10^-6^ |
| 14: 90075441-92098486 | 749 | Depression and ischemic stroke | 1.15×10^-4^ | 7.04×10^-6^ | -1.65 | -4.68×10^-5^ (1.14×10^-10^) | 1.21×10^-5^ |
| †The number of SNPs involved in the estimation of local genetic covariance in the genomic region. ‡The estimates of local genetic correlation may yield an estimate less than -1 or greater than 1 due to the insufficient study power. *Numbers in parentheses represent variance of the genetic covariance estimated by SUPERGNOVA. h^2^, heritability. | | | | | | | |

| **Supplementary Table 6. Lead SNPs for depression from MTAG analysis between depression and stroke.** | | | | | | | | | | | | | | |
| --- | --- | --- | --- | --- | --- | --- | --- | --- | --- | --- | --- | --- | --- | --- |
| SNP | CHR | BP | A1 | A2 | FREQ | Beta | SE | P | MTAG_Beta | MTAG_SE | MTAG_P | Novel | *r*^2^ | LD_Lead_SNP |
| rs4653218 | 1 | 37197319 | T | C | 0.422 | 0.021 | 0.003 | 2.78E-11 | 0.009 | 0.001 | 3.82E-11 | No | 0.301 | rs2089358 |
| rs12727100 | 1 | 49995988 | G | A | 0.906 | 0.035 | 0.006 | 6.95E-09 | 0.014 | 0.002 | 8.08E-09 | No | 0.928 | rs11579246 |
| rs11579246 | 1 | 50559162 | A | G | 0.888 | 0.033 | 0.005 | 1.06E-09 | 0.014 | 0.002 | 9.95E-10 | No | 1 | rs11579246 |
| rs1890946 | 1 | 52342427 | T | C | 0.473 | -0.022 | 0.003 | 2.79E-12 | -0.010 | 0.001 | 4.26E-12 | No | 1 | rs1890946 |
| rs672230 | 1 | 67224330 | T | C | 0.511 | 0.020 | 0.003 | 4.26E-09 | 0.008 | 0.001 | 2.46E-09 | No |  |  |
| rs7531118 | 1 | 72837239 | T | C | 0.465 | -0.031 | 0.003 | 1.19E-20 | -0.013 | 0.001 | 1.70E-20 | No | 1 | rs7531118 |
| rs11210054 | 1 | 73228715 | C | T | 0.851 | 0.025 | 0.004 | 4.65E-09 | 0.011 | 0.002 | 6.58E-09 | No | 0.063 | rs4571923 |
| rs12136984 | 1 | 73725998 | T | G | 0.499 | -0.026 | 0.003 | 2.53E-16 | -0.011 | 0.001 | 3.25E-16 | No | 0.877 | rs4571923 |
| rs2968485 | 1 | 96869933 | C | T | 0.311 | -0.024 | 0.004 | 3.36E-09 | -0.009 | 0.002 | 4.04E-09 | No | 0.218 | rs7513962 |
| rs1730864 | 1 | 107607810 | C | T | 0.325 | 0.019 | 0.003 | 1.10E-08 | 0.008 | 0.001 | 1.48E-08 | No |  |  |
| rs907662 | 1 | 117848822 | G | A | 0.761 | 0.022 | 0.004 | 2.66E-08 | 0.009 | 0.002 | 3.15E-08 | No |  |  |
| rs57022387 | 1 | 173847020 | G | T | 0.897 | 0.031 | 0.005 | 2.86E-09 | 0.013 | 0.002 | 5.15E-09 | No | 0.989 | rs16846561 |
| rs6680839 | 1 | 175902596 | C | T | 0.483 | 0.019 | 0.003 | 1.34E-08 | 0.008 | 0.001 | 8.82E-09 | No | 0.560 | rs10913112 |
| rs6682968 | 1 | 197608612 | C | T | 0.802 | 0.024 | 0.004 | 2.00E-09 | 0.010 | 0.002 | 2.96E-09 | No | 0.113 | rs2358493 |
| rs12028164 | 1 | 197820790 | C | A | 0.488 | -0.026 | 0.003 | 1.53E-15 | -0.011 | 0.001 | 2.04E-15 | No | 0.996 | rs2358493 |
| rs67446571 | 2 | 22554648 | A | G | 0.600 | -0.021 | 0.003 | 9.63E-11 | -0.009 | 0.001 | 1.72E-10 | No | 0.716 | rs12619197 |
| rs13394164 | 2 | 22960181 | A | G | 0.560 | -0.019 | 0.003 | 7.76E-09 | -0.008 | 0.001 | 1.09E-08 | No |  |  |
| rs10173817 | 2 | 51264319 | C | T | 0.456 | 0.018 | 0.003 | 1.12E-08 | 0.008 | 0.001 | 1.36E-08 | No | 0.594 | rs13031157 |
| rs7599039 | 2 | 51578355 | T | C | 0.797 | 0.021 | 0.004 | 2.95E-08 | 0.010 | 0.002 | 4.19E-08 | No | 1 | rs7599039 |
| rs4971586 | 2 | 52315585 | T | A | 0.534 | 0.018 | 0.003 | 2.60E-08 | 0.008 | 0.001 | 4.31E-08 | No | 1 | rs4971586 |
| rs11688767 | 2 | 57988194 | A | T | 0.526 | -0.018 | 0.003 | 1.15E-08 | -0.008 | 0.001 | 1.72E-08 | No | 0.893 | rs11682175 |
| rs4671458 | 2 | 63374063 | C | T | 0.769 | 0.022 | 0.004 | 2.84E-08 | 0.009 | 0.002 | 4.68E-08 | No | 1 | rs4671458 |
| rs1441102 | 2 | 104479862 | A | T | 0.476 | 0.021 | 0.003 | 2.20E-10 | 0.009 | 0.001 | 1.27E-10 | No | 0.328 | rs2570485 |
| rs62170849 | 2 | 125012926 | T | C | 0.707 | 0.023 | 0.004 | 1.39E-10 | 0.010 | 0.002 | 1.71E-10 | No | 1 | rs62170849 |
| rs6713237 | 2 | 142489456 | T | C | 0.141 | 0.026 | 0.005 | 1.10E-08 | 0.012 | 0.002 | 9.07E-09 | No |  |  |
| rs1320138 | 2 | 144158287 | T | C | 0.437 | 0.018 | 0.003 | 3.09E-08 | 0.008 | 0.001 | 2.90E-08 | No | 1 | rs1320138 |
| rs7564151 | 2 | 161356971 | G | C | 0.616 | 0.021 | 0.003 | 6.70E-10 | 0.009 | 0.001 | 5.07E-10 | No |  |  |
| rs1267079 | 2 | 162033854 | T | C | 0.696 | 0.022 | 0.003 | 1.95E-10 | 0.010 | 0.002 | 1.92E-10 | No | 1 | rs1267079 |
| rs4972656 | 2 | 175240292 | C | T | 0.415 | -0.019 | 0.003 | 3.23E-09 | -0.008 | 0.001 | 3.65E-09 | No | 1 | rs4972656 |
| rs2111593 | 2 | 208046998 | C | T | 0.645 | -0.023 | 0.003 | 3.46E-12 | -0.010 | 0.001 | 4.99E-12 | No | 0.827 | rs55772859 |
| rs55897719 | 2 | 212590841 | C | A | 0.682 | -0.019 | 0.003 | 3.14E-08 | -0.008 | 0.002 | 3.00E-08 | No |  |  |
| rs7629352 | 3 | 16848835 | A | G | 0.695 | -0.022 | 0.003 | 6.80E-11 | -0.010 | 0.002 | 7.00E-11 | No | 0.744 | rs6800583 |
| rs56029819 | 3 | 43478295 | T | C | 0.848 | -0.027 | 0.005 | 1.28E-09 | -0.012 | 0.002 | 1.25E-09 | No | 0.970 | rs77463213 |
| rs1105023 | 3 | 44222351 | T | G | 0.494 | 0.020 | 0.003 | 9.45E-10 | 0.008 | 0.001 | 1.41E-09 | No |  |  |
| rs6765468 | 3 | 44609626 | G | A | 0.670 | -0.024 | 0.003 | 5.20E-13 | -0.011 | 0.001 | 8.50E-13 | No | 0.867 | rs13058913 |
| rs7617480 | 3 | 49210732 | A | C | 0.209 | 0.032 | 0.004 | 2.11E-17 | 0.014 | 0.002 | 4.52E-17 | No | 1 | rs7617480 |
| rs7621797 | 3 | 49798864 | G | A | 0.844 | -0.034 | 0.004 | 8.67E-16 | -0.015 | 0.002 | 9.85E-16 | No | 0.514 | rs7617480 |
| rs3774364 | 3 | 52863605 | G | A | 0.617 | 0.018 | 0.003 | 1.80E-08 | 0.008 | 0.001 | 1.39E-08 | No | 0.381 | rs1010553 |
| rs66511648 | 3 | 117515519 | T | C | 0.738 | -0.023 | 0.004 | 1.25E-10 | -0.010 | 0.002 | 1.70E-10 | No | 0.941 | rs6783233 |
| rs13066362 | 3 | 158009477 | A | G | 0.556 | -0.019 | 0.003 | 1.69E-09 | -0.008 | 0.001 | 2.82E-09 | No |  |  |
| rs536445 | 3 | 173120103 | C | T | 0.473 | -0.018 | 0.003 | 8.66E-09 | -0.008 | 0.001 | 7.77E-09 | No | 1 | rs536445 |
| rs362280 | 4 | 3260057 | A | G | 0.528 | 0.018 | 0.003 | 2.50E-08 | 0.008 | 0.001 | 1.81E-08 | No | 0.268 | rs2298969 |
| rs114851235 | 4 | 59833409 | A | G | 0.941 | -0.048 | 0.008 | 2.70E-10 | -0.019 | 0.003 | 4.35E-10 | No | 1 | rs114851235 |
| rs7441547 | 4 | 59838120 | A | G | 0.511 | 0.019 | 0.003 | 6.11E-10 | 0.009 | 0.001 | 6.24E-10 | No |  |  |
| rs34207047 | 4 | 80199323 | C | A | 0.487 | 0.020 | 0.003 | 1.86E-10 | 0.009 | 0.001 | 2.96E-10 | No | 1 | rs34207047 |
| rs10026036 | 4 | 91091770 | C | A | 0.363 | -0.019 | 0.003 | 1.19E-08 | -0.008 | 0.001 | 1.78E-08 | No | 0.984 | rs6813752 |
| rs78689878 | 4 | 123096690 | G | A | 0.969 | 0.055 | 0.010 | 1.05E-08 | 0.023 | 0.004 | 1.18E-08 | No | 0.576 | rs45510091 |
| rs34973539 | 4 | 140940476 | T | A | 0.475 | 0.020 | 0.003 | 2.66E-10 | 0.009 | 0.001 | 3.06E-10 | No | 0.945 | rs6536630 |
| rs1128956 | 4 | 183724005 | T | G | 0.842 | 0.025 | 0.004 | 3.20E-09 | 0.011 | 0.002 | 3.10E-09 | No |  |  |
| rs993885 | 5 | 7379003 | G | A | 0.662 | -0.019 | 0.003 | 2.97E-08 | -0.008 | 0.001 | 3.38E-08 | No | 1 | rs2220599 |
| rs7700453 | 5 | 20126890 | G | C | 0.479 | 0.018 | 0.003 | 1.91E-08 | 0.008 | 0.001 | 1.06E-08 | No | 1 | rs7700453 |
| rs10057876 | 5 | 61487083 | C | T | 0.484 | -0.023 | 0.003 | 3.67E-13 | -0.010 | 0.001 | 7.46E-13 | No | 0.980 | rs12521969 |
| rs1644039 | 5 | 87909877 | C | T | 0.525 | 0.018 | 0.003 | 2.73E-08 | 0.008 | 0.001 | 4.36E-08 | No | 0.207 | rs17548339 |
| rs10514370 | 5 | 92435481 | G | T | 0.609 | 0.020 | 0.003 | 8.81E-10 | 0.009 | 0.001 | 1.01E-09 | No | 0.983 | rs4489042 |
| rs30266 | 5 | 103972357 | G | A | 0.687 | -0.037 | 0.004 | 2.78E-18 | -0.013 | 0.002 | 3.47E-18 | No |  |  |
| rs62379847 | 5 | 120109119 | A | C | 0.649 | -0.021 | 0.003 | 1.62E-09 | -0.009 | 0.001 | 2.33E-09 | No | 1 | rs62379847 |
| rs1993739 | 5 | 153215007 | C | T | 0.816 | 0.024 | 0.004 | 2.21E-08 | 0.010 | 0.002 | 2.20E-08 | No | 1 | rs2964003 |
| rs12189424 | 5 | 161316596 | A | G | 0.363 | 0.019 | 0.003 | 8.95E-09 | 0.008 | 0.001 | 7.15E-09 | No | 1 | rs2279020 |
| rs11135349 | 5 | 164523472 | A | C | 0.442 | -0.022 | 0.003 | 3.36E-12 | -0.010 | 0.001 | 7.03E-12 | No |  |  |
| rs17068508 | 5 | 166887538 | C | G | 0.957 | -0.051 | 0.008 | 1.99E-09 | -0.020 | 0.003 | 2.86E-09 | No | 0.055 | rs4869058 |
| rs2219498 | 5 | 167059459 | C | T | 0.534 | -0.018 | 0.003 | 1.06E-08 | -0.008 | 0.001 | 2.35E-08 | No |  |  |
| rs13198474 | 6 | 25874423 | G | A | 0.930 | 0.038 | 0.006 | 1.11E-09 | 0.017 | 0.003 | 1.65E-09 | No | 0.548 | rs35883476 |
| rs1624440 | 6 | 26433329 | G | A | 0.472 | 0.020 | 0.003 | 6.99E-10 | 0.008 | 0.001 | 1.70E-09 | No |  |  |
| rs72843784 | 6 | 26498758 | G | T | 0.921 | 0.047 | 0.006 | 2.11E-15 | 0.020 | 0.003 | 1.16E-14 | No | 0.752 | rs35883476 |
| rs67092078 | 6 | 27054772 | G | A | 0.920 | 0.048 | 0.006 | 7.18E-16 | 0.021 | 0.003 | 1.53E-15 | No | 0.767 | rs35883476 |
| rs17737641 | 6 | 27174557 | A | G | 0.972 | -0.061 | 0.009 | 2.54E-11 | -0.028 | 0.004 | 5.67E-11 | No | 0.063 | rs115938232 |
| rs7772160 | 6 | 27412386 | C | T | 0.482 | -0.022 | 0.003 | 7.73E-12 | -0.009 | 0.001 | 1.41E-11 | No | 0.086 | rs35883476 |
| rs115724600 | 6 | 27683371 | G | T | 0.976 | -0.054 | 0.010 | 1.65E-08 | -0.025 | 0.005 | 3.85E-08 | No | 0.136 | rs115938232 |
| rs17693963 | 6 | 27710165 | A | C | 0.908 | 0.050 | 0.006 | 8.12E-19 | 0.021 | 0.002 | 3.16E-18 | No | 0.682 | rs35883476 |
| rs13208096 | 6 | 28225311 | C | G | 0.917 | 0.052 | 0.006 | 5.70E-19 | 0.022 | 0.003 | 2.65E-18 | No | 0.969 | rs35883476 |
| rs140427260 | 6 | 28415601 | T | C | 0.959 | -0.045 | 0.008 | 9.98E-09 | -0.020 | 0.004 | 1.95E-08 | No | 0.242 | rs115938232 |
| rs9257184 | 6 | 28756088 | C | T | 0.915 | 0.056 | 0.006 | 8.42E-22 | 0.024 | 0.003 | 6.27E-21 | No | 0.926 | rs35883476 |
| rs9257405 | 6 | 28922437 | T | A | 0.608 | -0.019 | 0.003 | 4.84E-09 | -0.008 | 0.001 | 4.33E-09 | No |  |  |
| rs149142151 | 6 | 28941134 | C | T | 0.965 | -0.054 | 0.008 | 2.14E-11 | -0.025 | 0.004 | 4.72E-11 | No | 0.340 | rs115938232 |
| rs9257809 | 6 | 29356331 | A | G | 0.910 | 0.057 | 0.006 | 7.99E-23 | 0.024 | 0.002 | 2.72E-22 | No | 0.740 | rs35883476 |
| rs115938232 | 6 | 29725013 | A | G | 0.951 | -0.052 | 0.007 | 7.62E-14 | -0.024 | 0.003 | 1.24E-13 | No | 1 | rs115938232 |
| rs3094146 | 6 | 29970960 | G | C | 0.903 | 0.052 | 0.006 | 1.49E-19 | 0.021 | 0.002 | 3.95E-19 | No | 0.632 | rs35883476 |
| rs3130396 | 6 | 30223490 | T | C | 0.574 | 0.019 | 0.003 | 2.52E-09 | 0.008 | 0.001 | 3.83E-09 | No | 0.079 | rs35883476 |
| rs28780098 | 6 | 30303368 | G | T | 0.947 | -0.043 | 0.007 | 1.15E-10 | -0.020 | 0.003 | 1.68E-10 | No | 0.633 | rs115938232 |
| rs3131781 | 6 | 30937732 | A | G | 0.884 | 0.037 | 0.005 | 6.11E-12 | 0.015 | 0.002 | 1.68E-11 | No | 0.485 | rs35883476 |
| rs13207315 | 6 | 31241127 | T | C | 0.881 | -0.028 | 0.005 | 3.27E-09 | -0.013 | 0.002 | 5.92E-09 | No |  |  |
| rs2736155 | 6 | 31605199 | G | C | 0.547 | -0.018 | 0.003 | 4.60E-08 | -0.008 | 0.001 | 4.09E-08 | No |  |  |
| rs9368699 | 6 | 31802541 | T | C | 0.962 | -0.045 | 0.008 | 1.94E-08 | -0.021 | 0.004 | 2.13E-08 | No | 0.064 | rs115938232 |
| rs537362 | 6 | 50650700 | G | C | 0.082 | 0.031 | 0.006 | 1.70E-08 | 0.015 | 0.003 | 1.35E-08 | No | 1 | rs537362 |
| rs72904286 | 6 | 51414922 | C | T | 0.889 | 0.030 | 0.005 | 9.11E-09 | 0.013 | 0.002 | 1.30E-08 | No | 0.971 | rs17752199 |
| rs632024 | 6 | 64630933 | T | G | 0.294 | -0.020 | 0.003 | 7.05E-09 | -0.009 | 0.002 | 6.30E-09 | No | 1 | rs632024 |
| rs2214123 | 6 | 67000001 | A | G | 0.360 | 0.022 | 0.003 | 5.81E-11 | 0.009 | 0.001 | 1.31E-10 | No |  |  |
| rs9322279 | 6 | 101347943 | G | T | 0.531 | 0.019 | 0.003 | 7.94E-10 | 0.009 | 0.001 | 5.23E-10 | No | 0.722 | rs12530388 |
| rs12110689 | 6 | 103025230 | A | G | 0.607 | 0.018 | 0.003 | 2.38E-08 | 0.008 | 0.001 | 3.37E-08 | No |  |  |
| rs728017 | 6 | 124292594 | A | G | 0.411 | -0.019 | 0.003 | 8.03E-09 | -0.008 | 0.001 | 5.45E-09 | No | 1 | rs728017 |
| rs2876520 | 6 | 142996618 | C | G | 0.541 | -0.019 | 0.003 | 3.70E-09 | -0.008 | 0.001 | 4.55E-09 | No | 1 | rs2876520 |
| rs9373363 | 6 | 143150043 | A | G | 0.723 | 0.022 | 0.004 | 4.17E-09 | 0.009 | 0.002 | 6.53E-09 | No |  |  |
| rs11756123 | 6 | 152218079 | A | T | 0.359 | 0.025 | 0.003 | 3.10E-14 | 0.011 | 0.001 | 2.55E-14 | No | 1 | rs9479138 |
| rs12190823 | 6 | 163092643 | G | A | 0.770 | -0.021 | 0.004 | 1.98E-08 | -0.009 | 0.002 | 1.35E-08 | No | 1 | rs12190823 |
| rs76149263 | 6 | 165178503 | C | T | 0.718 | -0.022 | 0.004 | 1.32E-09 | -0.010 | 0.002 | 9.56E-10 | No | 0.270 | rs2322702 |
| rs868754 | 7 | 2033780 | G | C | 0.784 | 0.034 | 0.004 | 4.18E-17 | 0.014 | 0.002 | 4.24E-17 | No | 0.703 | rs10268609 |
| rs1637736 | 7 | 2246454 | T | C | 0.379 | -0.019 | 0.003 | 8.26E-09 | -0.008 | 0.001 | 1.28E-08 | No |  |  |
| rs957360 | 7 | 3660918 | C | G | 0.699 | 0.020 | 0.004 | 7.66E-09 | 0.009 | 0.002 | 6.51E-09 | No | 1 | rs957360 |
| rs6460906 | 7 | 12283276 | T | C | 0.602 | -0.023 | 0.003 | 3.38E-12 | -0.010 | 0.001 | 8.51E-13 | No | 1 | rs6460905 |
| rs2247523 | 7 | 82454404 | C | G | 0.526 | -0.019 | 0.003 | 5.24E-09 | -0.008 | 0.001 | 1.02E-08 | No | 0.547 | rs12707530 |
| rs2894699 | 7 | 114059156 | C | T | 0.559 | 0.022 | 0.003 | 6.65E-12 | 0.010 | 0.001 | 5.85E-12 | No | 0.973 | rs8180817 |
| rs1548461 | 7 | 117567576 | T | C | 0.471 | -0.020 | 0.003 | 4.84E-10 | -0.009 | 0.001 | 6.88E-10 | No | 1 | rs1548461 |
| rs28897173 | 8 | 14218081 | T | C | 0.722 | 0.021 | 0.004 | 2.71E-09 | 0.009 | 0.002 | 3.22E-09 | No | 0.963 | rs34269011 |
| rs2256035 | 8 | 14628926 | A | G | 0.388 | -0.018 | 0.003 | 1.84E-08 | -0.008 | 0.001 | 3.47E-08 | No |  |  |
| rs10098006 | 8 | 65572919 | C | G | 0.144 | -0.026 | 0.004 | 3.79E-09 | -0.012 | 0.002 | 3.54E-09 | No | 1 | rs7837935 |
| rs4628229 | 8 | 129882550 | T | C | 0.308 | -0.020 | 0.004 | 1.49E-08 | -0.009 | 0.002 | 1.38E-08 | No | 0.986 | rs6470670 |
| rs72694255 | 9 | 11130263 | G | C | 0.896 | 0.034 | 0.006 | 5.85E-10 | 0.014 | 0.002 | 8.58E-10 | No | 0.081 | rs1931388 |
| rs10809385 | 9 | 11208995 | C | A | 0.592 | 0.026 | 0.003 | 8.03E-16 | 0.012 | 0.001 | 5.13E-16 | No | 1 | rs1931388 |
| rs62555663 | 9 | 11724099 | C | A | 0.799 | 0.026 | 0.004 | 2.86E-11 | 0.012 | 0.002 | 4.26E-11 | No | 0.134 | rs1931388 |
| rs3793577 | 9 | 23737627 | A | G | 0.473 | -0.022 | 0.003 | 5.04E-12 | -0.010 | 0.001 | 8.70E-12 | No | 1 | rs3793577 |
| rs13296641 | 9 | 31160243 | G | A | 0.684 | 0.020 | 0.003 | 2.30E-09 | 0.009 | 0.002 | 3.11E-09 | No | 1 | rs13296641 |
| rs1329572 | 9 | 37001471 | T | A | 0.583 | -0.024 | 0.003 | 2.71E-13 | -0.010 | 0.001 | 2.65E-13 | No | 1 | rs1329572 |
| rs62535714 | 9 | 37182655 | G | A | 0.811 | -0.032 | 0.004 | 1.40E-14 | -0.014 | 0.002 | 2.51E-14 | No | 0.075 | rs1329572 |
| rs6479487 | 9 | 96237373 | T | G | 0.839 | -0.030 | 0.004 | 2.60E-12 | -0.013 | 0.002 | 2.52E-12 | No | 0.932 | rs7023933 |
| rs10821163 | 9 | 96343060 | G | C | 0.645 | 0.022 | 0.003 | 1.09E-10 | 0.009 | 0.001 | 2.16E-10 | No | 0.086 | rs7023933 |
| rs182923402 | 9 | 98299677 | T | A | 0.911 | -0.036 | 0.006 | 8.17E-10 | -0.015 | 0.002 | 8.03E-10 | No | 1 | rs182923402 |
| rs1888286 | 9 | 119726051 | T | C | 0.707 | 0.022 | 0.004 | 5.78E-10 | 0.009 | 0.002 | 8.53E-10 | No | 0.943 | rs2418449 |
| rs7025421 | 9 | 122666288 | C | T | 0.392 | -0.021 | 0.003 | 3.29E-11 | -0.010 | 0.001 | 2.54E-11 | No | 0.963 | rs12553064 |
| rs73581580 | 9 | 140251458 | G | A | 0.850 | -0.031 | 0.005 | 4.83E-10 | -0.012 | 0.002 | 3.50E-10 | No | 0.801 | rs112535944 |
| rs4620605 | 10 | 68532027 | C | T | 0.196 | 0.022 | 0.004 | 2.06E-08 | 0.010 | 0.002 | 1.60E-08 | No | 0.987 | rs4515864 |
| rs3088142 | 10 | 76854564 | C | T | 0.570 | -0.019 | 0.003 | 5.98E-09 | -0.008 | 0.001 | 6.43E-09 | No | 1 | rs3088142 |
| rs1909696 | 10 | 77582203 | G | T | 0.323 | -0.019 | 0.003 | 2.54E-08 | -0.008 | 0.001 | 3.58E-08 | No | 1 | rs1909696 |
| rs11191658 | 10 | 105096021 | A | G | 0.678 | 0.020 | 0.004 | 2.30E-08 | 0.008 | 0.001 | 3.90E-08 | No |  |  |
| rs2151971 | 10 | 106445916 | C | T | 0.501 | 0.019 | 0.003 | 7.18E-10 | 0.009 | 0.001 | 5.79E-10 | No | 0.109 | rs1021363 |
| rs11192191 | 10 | 106554691 | T | G | 0.781 | -0.022 | 0.004 | 7.82E-09 | -0.010 | 0.002 | 8.37E-09 | No | 0.081 | rs1021363 |
| rs1021363 | 10 | 106610839 | A | G | 0.329 | 0.030 | 0.003 | 4.93E-19 | 0.013 | 0.001 | 3.65E-18 | No | 1 | rs1021363 |
| rs10400180 | 10 | 106755196 | T | G | 0.773 | 0.027 | 0.004 | 3.53E-12 | 0.012 | 0.002 | 4.77E-12 | No | 0.501 | rs17186548 |
| rs111442587 | 10 | 114984089 | C | G | 0.975 | -0.059 | 0.011 | 2.33E-08 | -0.025 | 0.004 | 2.09E-08 | No | 1 | rs111442587 |
| rs10835361 | 11 | 28618043 | C | G | 0.626 | 0.026 | 0.003 | 2.35E-15 | 0.011 | 0.001 | 2.57E-15 | No | 0.876 | rs11030380 |
| rs927456 | 11 | 29770417 | T | C | 0.675 | -0.020 | 0.003 | 9.42E-09 | -0.009 | 0.001 | 6.75E-09 | No | 0.390 | rs324300 |
| rs145678014 | 11 | 32927778 | G | T | 0.962 | 0.050 | 0.008 | 5.58E-10 | 0.023 | 0.004 | 7.16E-10 | No | 0.608 | rs80067584 |
| rs2509805 | 11 | 57650796 | T | C | 0.334 | 0.023 | 0.003 | 1.61E-11 | 0.010 | 0.001 | 1.59E-11 | No | 1 | rs2509805 |
| rs174546 | 11 | 61569830 | C | T | 0.637 | -0.019 | 0.003 | 1.36E-08 | -0.008 | 0.001 | 2.23E-08 | No | 0.933 | rs102275 |
| rs61903412 | 11 | 88890451 | G | A | 0.490 | 0.021 | 0.003 | 1.60E-11 | 0.009 | 0.001 | 3.06E-11 | No | 0.456 | rs4753209 |
| rs1940729 | 11 | 112852192 | T | C | 0.533 | -0.021 | 0.003 | 1.86E-10 | -0.009 | 0.001 | 8.44E-11 | No | 0.865 | rs4937872 |
| rs11214594 | 11 | 113259332 | G | A | 0.481 | 0.024 | 0.003 | 6.31E-14 | 0.010 | 0.001 | 9.22E-14 | No | 0.069 | rs61902811 |
| rs61902811 | 11 | 113370758 | G | A | 0.684 | 0.028 | 0.003 | 1.54E-17 | 0.013 | 0.002 | 9.80E-18 | No | 1 | rs61902811 |
| rs73004016 | 11 | 113444727 | T | C | 0.889 | 0.035 | 0.005 | 9.42E-11 | 0.015 | 0.002 | 7.21E-11 | No |  |  |
| rs7940164 | 11 | 113451765 | G | T | 0.336 | 0.022 | 0.003 | 9.42E-11 | 0.010 | 0.001 | 9.69E-11 | No | 0.134 | rs7130926 |
| rs612823 | 11 | 133834104 | T | C | 0.579 | 0.023 | 0.003 | 9.20E-12 | 0.010 | 0.001 | 8.37E-12 | No | 1 | rs612823 |
| rs11169666 | 12 | 39211300 | T | C | 0.579 | 0.018 | 0.003 | 3.60E-08 | 0.008 | 0.001 | 3.25E-08 | No |  |  |
| rs11612312 | 12 | 52349088 | T | C | 0.800 | -0.025 | 0.004 | 3.04E-10 | -0.011 | 0.002 | 1.11E-09 | No | 1 | rs11612312 |
| rs11172247 | 12 | 57946510 | G | C | 0.409 | -0.018 | 0.003 | 4.29E-08 | -0.008 | 0.001 | 4.67E-08 | No | 0.253 | rs73123350 |
| rs2292996 | 12 | 103556972 | T | C | 0.462 | -0.018 | 0.003 | 3.77E-08 | -0.008 | 0.001 | 3.98E-08 | No | 1 | rs2292996 |
| rs521048 | 12 | 118830958 | A | G | 0.268 | -0.023 | 0.004 | 2.12E-09 | -0.010 | 0.002 | 1.73E-09 | No | 1 | rs521048 |
| rs4457791 | 12 | 118877949 | T | G | 0.834 | -0.024 | 0.004 | 5.62E-08 | -0.010 | 0.002 | 4.16E-08 | No | 0.083 | rs521048 |
| rs58291418 | 12 | 121264010 | C | T | 0.528 | -0.019 | 0.003 | 8.13E-09 | -0.008 | 0.001 | 9.92E-09 | No | 0.984 | rs3213572 |
| rs9530139 | 13 | 31847324 | C | T | 0.779 | 0.024 | 0.004 | 1.30E-09 | 0.010 | 0.002 | 1.57E-09 | No | 0.860 | rs7334101 |
| rs9527064 | 13 | 53897024 | G | A | 0.672 | -0.021 | 0.003 | 1.00E-09 | -0.009 | 0.001 | 1.82E-09 | No | 0.828 | rs9536383 |
| rs508502 | 13 | 80921519 | C | T | 0.689 | 0.023 | 0.004 | 8.69E-11 | 0.010 | 0.002 | 1.11E-10 | No | 1 | rs508502 |
| rs9561331 | 13 | 94017476 | G | A | 0.876 | -0.027 | 0.005 | 1.06E-08 | -0.012 | 0.002 | 9.39E-09 | No | 1 | rs9561328 |
| rs510339 | 13 | 97286612 | G | A | 0.164 | -0.024 | 0.004 | 1.39E-08 | -0.011 | 0.002 | 1.46E-08 | No | 0.484 | rs7317645 |
| rs4772087 | 13 | 99115041 | C | T | 0.615 | -0.019 | 0.003 | 1.29E-08 | -0.008 | 0.001 | 1.85E-08 | No | 1 | rs4772087 |
| rs1111177 | 14 | 42076337 | A | G | 0.495 | 0.024 | 0.003 | 1.37E-14 | 0.011 | 0.001 | 7.85E-15 | No | 0.953 | rs11157241 |
| rs2933195 | 14 | 47293348 | A | G | 0.553 | -0.019 | 0.003 | 4.25E-09 | -0.008 | 0.001 | 4.66E-09 | No |  |  |
| rs2274793 | 14 | 57283968 | C | T | 0.666 | 0.020 | 0.003 | 2.63E-09 | 0.009 | 0.001 | 3.49E-09 | No | 1 | rs2274794 |
| rs11621186 | 14 | 75120628 | C | T | 0.504 | -0.022 | 0.003 | 6.07E-12 | -0.010 | 0.001 | 9.74E-12 | No | 1 | rs11621186 |
| rs2896460 | 14 | 103306106 | A | T | 0.752 | -0.023 | 0.004 | 1.13E-09 | -0.010 | 0.002 | 1.70E-09 | No | 0.947 | rs9671376 |
| rs10149470 | 14 | 104017953 | A | G | 0.485 | -0.023 | 0.003 | 3.02E-12 | -0.010 | 0.001 | 1.09E-11 | No | 1 | rs10149470 |
| rs111280186 | 14 | 104357988 | C | G | 0.877 | -0.027 | 0.005 | 2.50E-08 | -0.012 | 0.002 | 2.56E-08 | No | 0.070 | rs10149470 |
| rs56059718 | 15 | 38836777 | C | A | 0.806 | -0.022 | 0.004 | 2.24E-08 | -0.010 | 0.002 | 3.17E-08 | No | 1 | rs56059718 |
| rs12901436 | 15 | 47683095 | C | G | 0.784 | -0.024 | 0.004 | 1.04E-09 | -0.010 | 0.002 | 1.02E-09 | No | 1 | rs34488670 |
| rs74927570 | 15 | 63802297 | T | G | 0.805 | -0.025 | 0.004 | 7.94E-10 | -0.011 | 0.002 | 5.15E-10 | No | 0.686 | rs79714693 |
| rs4776729 | 15 | 66399197 | A | G | 0.876 | -0.028 | 0.005 | 5.69E-09 | -0.012 | 0.002 | 9.80E-09 | No | 1 | rs4776729 |
| rs4404022 | 15 | 74010430 | A | T | 0.600 | -0.019 | 0.003 | 6.92E-09 | -0.008 | 0.001 | 5.09E-09 | No |  |  |
| rs72743363 | 15 | 74104811 | G | A | 0.863 | 0.033 | 0.005 | 3.59E-12 | 0.014 | 0.002 | 4.00E-12 | No | 0.651 | rs8030745 |
| rs8035777 | 15 | 88943807 | A | G | 0.778 | 0.022 | 0.004 | 7.09E-09 | 0.010 | 0.002 | 7.00E-09 | No | 0.683 | rs11855600 |
| rs4702 | 15 | 91426560 | G | A | 0.452 | 0.022 | 0.003 | 7.64E-12 | 0.009 | 0.001 | 2.33E-11 | No | 1 | rs4702 |
| rs12935276 | 16 | 13038723 | T | G | 0.708 | -0.022 | 0.004 | 1.63E-09 | -0.009 | 0.002 | 2.03E-09 | No | 0.911 | rs7200826 |
| rs12919291 | 16 | 13800430 | G | C | 0.821 | -0.023 | 0.004 | 2.04E-08 | -0.010 | 0.002 | 2.13E-08 | No |  |  |
| rs2369818 | 16 | 21614009 | C | T | 0.581 | -0.022 | 0.003 | 1.94E-11 | -0.009 | 0.001 | 3.35E-11 | No | 1 | rs2369818 |
| rs55730016 | 16 | 49495173 | C | T | 0.647 | -0.019 | 0.004 | 3.77E-08 | -0.008 | 0.001 | 2.86E-08 | No | 0.576 | rs4785307 |
| rs2111568 | 16 | 49498861 | G | C | 0.721 | -0.022 | 0.004 | 1.75E-08 | -0.009 | 0.002 | 3.22E-08 | No | 0.085 | rs4785307 |
| rs1862743 | 16 | 60743834 | C | A | 0.511 | 0.020 | 0.003 | 7.43E-10 | 0.009 | 0.001 | 4.99E-10 | No |  |  |
| rs407502 | 17 | 31539581 | G | A | 0.283 | -0.021 | 0.004 | 1.27E-08 | -0.009 | 0.002 | 1.81E-08 | No | 0.887 | rs412891 |
| rs9915591 | 17 | 65826090 | C | G | 0.785 | -0.024 | 0.004 | 1.28E-09 | -0.010 | 0.002 | 1.34E-09 | No | 0.757 | rs60856912 |
| rs11081827 | 18 | 31390228 | C | A | 0.517 | -0.020 | 0.003 | 3.01E-10 | -0.009 | 0.001 | 2.80E-10 | No | 0.859 | rs10460051 |
| rs12967855 | 18 | 35138245 | A | G | 0.315 | 0.028 | 0.003 | 1.64E-16 | 0.012 | 0.002 | 1.84E-16 | No | 1 | rs12967855 |
| rs4267411 | 18 | 39305256 | C | T | 0.827 | 0.026 | 0.004 | 4.69E-10 | 0.012 | 0.002 | 4.08E-10 | No | 1 | rs4267411 |
| rs7232516 | 18 | 50389194 | G | A | 0.864 | 0.027 | 0.005 | 1.39E-08 | 0.011 | 0.002 | 2.17E-08 | No | 0.064 | rs7230285 |
| rs4632195 | 18 | 50746748 | C | T | 0.497 | -0.027 | 0.003 | 6.85E-18 | -0.012 | 0.001 | 2.92E-17 | No | 0.942 | rs7230285 |
| rs72925321 | 18 | 50807202 | G | A | 0.934 | 0.046 | 0.007 | 1.67E-11 | 0.019 | 0.003 | 1.14E-11 | No |  |  |
| rs8093506 | 18 | 52474170 | A | G | 0.733 | 0.028 | 0.004 | 1.64E-13 | 0.012 | 0.002 | 1.69E-13 | No | 0.700 | rs1819881 |
| rs4128242 | 18 | 52747689 | T | C | 0.581 | 0.024 | 0.003 | 5.44E-14 | 0.011 | 0.001 | 3.48E-14 | No | 1 | rs4131791 |
| rs1942569 | 18 | 52846725 | T | G | 0.518 | -0.019 | 0.003 | 9.74E-10 | -0.008 | 0.001 | 2.52E-09 | No | 0.087 | rs12967143 |
| rs12967143 | 18 | 53099012 | G | C | 0.287 | 0.030 | 0.004 | 7.52E-18 | 0.013 | 0.002 | 3.07E-17 | No | 1 | rs12967143 |
| rs896686 | 18 | 53123031 | T | G | 0.819 | 0.027 | 0.004 | 2.27E-10 | 0.011 | 0.002 | 2.96E-10 | No | 0.077 | rs12967143 |
| rs1452787 | 18 | 53207207 | A | G | 0.714 | -0.024 | 0.004 | 4.69E-12 | -0.011 | 0.002 | 8.31E-12 | No |  |  |
| rs7241572 | 18 | 77580712 | G | A | 0.794 | -0.029 | 0.005 | 5.39E-10 | -0.011 | 0.002 | 9.09E-10 | No | 1 | rs7241572 |
| rs148108087 | 19 | 51781455 | G | A | 0.937 | 0.041 | 0.007 | 1.90E-08 | 0.016 | 0.003 | 2.57E-08 | No |  |  |
| rs6131010 | 20 | 44724305 | A | G | 0.268 | 0.027 | 0.004 | 3.01E-14 | 0.012 | 0.002 | 2.09E-14 | No | 1 | rs6131010 |
| rs910187 | 20 | 45841052 | G | A | 0.640 | 0.020 | 0.003 | 4.39E-09 | 0.008 | 0.001 | 8.42E-09 | No | 1 | rs910187 |
| rs13056300 | 22 | 41408754 | T | C | 0.719 | -0.023 | 0.004 | 2.53E-10 | -0.010 | 0.002 | 2.52E-10 | No | 0.770 | rs2273085 |
| rs12160976 | 22 | 46438246 | G | A | 0.668 | 0.022 | 0.004 | 2.71E-09 | 0.009 | 0.001 | 3.73E-09 | No | 0.417 | rs9627391 |
| SNP, single nucleotide polymorphisms; CHR, chromosome; BP, physical position of SNP (base-pairs); A1, effect allele; A2, alternative allele; EAF: effect allele frequency; Beta, effect allele beta coefficient; SE, standard error of the beta coefficient. A lead SNP was considered to be novel if the SNP did not reach genome-wide significance in the original single-trait GWAS or the SNP was independent (*r*^2^ < 0.1) of those previously reported genome-wide significant SNPs for single traits. | | | | | | | | | | | | | | |

| **Supplementary Table 7. Lead SNPs for stroke from MTAG analysis between depression and stroke.** | | | | | | | | | | | | | | |
| --- | --- | --- | --- | --- | --- | --- | --- | --- | --- | --- | --- | --- | --- | --- |
| SNP | CHR | BP | A1 | A2 | FREQ | Beta | SE | P | MTAG_Beta | MTAG_SE | MTAG_P | Novel | *r*^2^ | LD_Lead_SNP |
| rs2500281 | 1 | 3220382 | G | A | 0.246 | 0.060 | 0.010 | 3.95E-09 | -0.008 | 0.001 | 2.41E-08 | No | 1 | rs2455132 |
| rs880315 | 1 | 10796866 | T | C | 0.633 | -0.044 | 0.007 | 2.89E-10 | -0.008 | 0.001 | 5.59E-10 | No |  |  |
| rs6427304 | 1 | 156156789 | A | G | 0.645 | 0.053 | 0.007 | 7.13E-15 | 0.010 | 0.001 | 7.20E-14 | No | 0.883 | rs2251636 |
| rs115628302 | 2 | 203690047 | T | G | 0.874 | 0.062 | 0.010 | 1.82E-10 | 0.011 | 0.002 | 7.14E-10 | No | 0.983 | rs2351524 |
| rs72900157 | 4 | 111710793 | C | G | 0.874 | -0.089 | 0.010 | 4.50E-20 | -0.016 | 0.002 | 1.10E-17 | No | 0.567 | rs6847935 |
| rs56010410 | 4 | 155502869 | T | C | 0.731 | -0.056 | 0.007 | 1.14E-14 | -0.010 | 0.001 | 2.26E-13 | No | 0.216 | rs6536024 |
| rs17148926 | 5 | 121510586 | A | C | 0.824 | 0.060 | 0.009 | 1.02E-11 | 0.009 | 0.002 | 4.09E-09 | No | 1 | rs17148926 |
| rs79318212 | 6 | 1365244 | A | G | 0.885 | -0.078 | 0.011 | 2.79E-13 | -0.012 | 0.002 | 5.76E-11 | No | 1 | rs79318212 |
| rs2501966 | 6 | 49457835 | A | G | 0.595 | 0.038 | 0.007 | 5.12E-09 | 0.007 | 0.001 | 1.79E-08 | No | 1 | rs2501966 |
| rs7808568 | 7 | 12271252 | T | C | 0.598 | -0.031 | 0.007 | 3.00E-06 | -0.007 | 0.001 | 4.58E-09 | Yes |  |  |
| rs42043 | 7 | 92247333 | A | G | 0.751 | 0.049 | 0.008 | 1.30E-10 | 0.009 | 0.001 | 6.24E-10 | No | 0.975 | rs42035 |
| rs1549758 | 7 | 150695726 | T | C | 0.302 | 0.045 | 0.008 | 4.46E-09 | 0.008 | 0.001 | 1.02E-08 | No | 1 | rs1549758 |
| rs944797 | 9 | 22115286 | T | C | 0.510 | -0.049 | 0.006 | 2.27E-14 | -0.009 | 0.001 | 6.47E-14 | No | 0.965 | rs7859362 |
| rs532436 | 9 | 136149830 | G | A | 0.803 | 0.057 | 0.008 | 1.16E-12 | -0.009 | 0.002 | 1.13E-09 | No | 0.829 | rs649129 |
| rs55983834 | 10 | 105601210 | C | T | 0.653 | -0.048 | 0.008 | 4.02E-09 | 0.007 | 0.001 | 3.30E-08 | No | 1 | rs55983834 |
| rs10886430 | 10 | 121010256 | A | G | 0.876 | -0.069 | 0.011 | 9.31E-11 | -0.012 | 0.002 | 3.22E-10 | No | 1 | rs10886430 |
| rs150986675 | 11 | 102777303 | T | C | 0.851 | -0.056 | 0.009 | 1.08E-09 | -0.010 | 0.002 | 6.40E-09 | No | 0.581 | rs72985562 |
| rs10774625 | 12 | 111910219 | A | G | 0.480 | 0.055 | 0.007 | 6.23E-17 | 0.009 | 0.001 | 5.38E-14 | No | 1 | rs10774625 |
| rs17696736 | 12 | 112486818 | A | G | 0.571 | -0.050 | 0.007 | 1.53E-14 | -0.008 | 0.001 | 3.98E-11 | No | 0.782 | rs10774625 |
| rs1975514 | 13 | 110828891 | T | C | 0.638 | -0.038 | 0.007 | 1.51E-08 | -0.007 | 0.001 | 1.67E-08 | No |  |  |
| rs12445022 | 16 | 87575332 | G | A | 0.670 | 0.044 | 0.007 | 5.65E-10 | -0.007 | 0.001 | 2.20E-08 | No | 1 | rs12445022 |
| rs17677363 | 17 | 45036112 | A | T | 0.863 | -0.048 | 0.009 | 2.96E-07 | -0.010 | 0.002 | 3.41E-08 | Yes |  |  |
| rs11906160 | 20 | 33565755 | G | A | 0.879 | -0.063 | 0.010 | 2.99E-10 | 0.012 | 0.002 | 6.20E-10 | No | 0.769 | rs11907011 |
| SNP, single nucleotide polymorphisms; CHR, chromosome; BP, physical position of SNP (base-pairs); A1, effect allele; A2, alternative allele; EAF: effect allele frequency; Beta, effect allele beta coefficient; SE, standard error of the beta coefficient. A lead SNP was considered to be novel if the SNP did not reach genome-wide significance in the original single-trait GWAS or the SNP was independent (*r*^2^ < 0.1) of those previously reported genome-wide significant SNPs for single traits. | | | | | | | | | | | | | | |

| **Supplementary Table 8. Lead SNPs for depression from MTAG analysis between depression and ischemic stroke.** | | | | | | | | | | | | | | |
| --- | --- | --- | --- | --- | --- | --- | --- | --- | --- | --- | --- | --- | --- | --- |
| SNP | CHR | BP | A1 | A2 | FREQ | Beta | SE | P | MTAG_Beta | MTAG_SE | MTAG_P | Novel | *r*^2^ | LD_Lead_SNP |
| rs4653218 | 1 | 37197319 | T | C | 0.422 | 0.021 | 0.003 | 2.78E-11 | 0.009 | 0.001 | 4.31E-11 | No | 0.301 | rs2089358 |
| rs12727100 | 1 | 49995988 | G | A | 0.906 | 0.035 | 0.006 | 6.95E-09 | 0.014 | 0.002 | 8.92E-09 | No | 0.928 | rs11579246 |
| rs11579246 | 1 | 50559162 | A | G | 0.888 | 0.033 | 0.005 | 1.06E-09 | 0.014 | 0.002 | 1.11E-09 | No | 1 | rs11579246 |
| rs1890946 | 1 | 52342427 | T | C | 0.473 | -0.022 | 0.003 | 2.79E-12 | -0.010 | 0.001 | 3.76E-12 | No | 1 | rs1890946 |
| rs672230 | 1 | 67224330 | T | C | 0.511 | 0.020 | 0.003 | 4.26E-09 | 0.008 | 0.001 | 2.54E-09 | No |  |  |
| rs7531118 | 1 | 72837239 | T | C | 0.465 | -0.031 | 0.003 | 1.19E-20 | -0.013 | 0.001 | 1.35E-20 | No | 1 | rs7531118 |
| rs11210054 | 1 | 73228715 | C | T | 0.851 | 0.025 | 0.004 | 4.65E-09 | 0.011 | 0.002 | 5.42E-09 | No | 0.063 | rs4571923 |
| rs12136984 | 1 | 73725998 | T | G | 0.499 | -0.026 | 0.003 | 2.53E-16 | -0.011 | 0.001 | 3.26E-16 | No | 0.877 | rs4571923 |
| rs2968485 | 1 | 96869933 | C | T | 0.311 | -0.024 | 0.004 | 3.36E-09 | -0.009 | 0.002 | 4.14E-09 | No | 0.218 | rs7513962 |
| rs1730864 | 1 | 107607810 | C | T | 0.325 | 0.019 | 0.003 | 1.10E-08 | 0.008 | 0.001 | 1.42E-08 | No |  |  |
| rs907662 | 1 | 117848822 | G | A | 0.761 | 0.022 | 0.004 | 2.66E-08 | 0.009 | 0.002 | 3.25E-08 | No |  |  |
| rs57022387 | 1 | 173847020 | G | T | 0.897 | 0.031 | 0.005 | 2.86E-09 | 0.013 | 0.002 | 4.78E-09 | No | 0.989 | rs16846561 |
| rs6680839 | 1 | 175902596 | C | T | 0.483 | 0.019 | 0.003 | 1.34E-08 | 0.008 | 0.001 | 8.55E-09 | No | 0.560 | rs10913112 |
| rs6682968 | 1 | 197608612 | C | T | 0.802 | 0.024 | 0.004 | 2.00E-09 | 0.010 | 0.002 | 2.83E-09 | No | 0.113 | rs2358493 |
| rs12028164 | 1 | 197820790 | C | A | 0.488 | -0.026 | 0.003 | 1.53E-15 | -0.011 | 0.001 | 2.04E-15 | No | 0.996 | rs2358493 |
| rs67446571 | 2 | 22554648 | A | G | 0.600 | -0.021 | 0.003 | 9.63E-11 | -0.009 | 0.001 | 1.90E-10 | No | 0.716 | rs12619197 |
| rs13394164 | 2 | 22960181 | A | G | 0.560 | -0.019 | 0.003 | 7.76E-09 | -0.008 | 0.001 | 1.17E-08 | No |  |  |
| rs10173817 | 2 | 51264319 | C | T | 0.456 | 0.018 | 0.003 | 1.12E-08 | 0.008 | 0.001 | 1.31E-08 | No | 0.594 | rs13031157 |
| rs7598476 | 2 | 51588582 | T | C | 0.652 | 0.018 | 0.003 | 4.45E-08 | 0.008 | 0.001 | 4.30E-08 | No | 0.523 | rs7599039 |
| rs4971586 | 2 | 52315585 | T | A | 0.534 | 0.018 | 0.003 | 2.60E-08 | 0.008 | 0.001 | 4.27E-08 | No | 1 | rs4971586 |
| rs11682175 | 2 | 57987593 | T | C | 0.551 | -0.018 | 0.003 | 9.65E-09 | -0.008 | 0.001 | 1.74E-08 | No | 1 | rs11682175 |
| rs4671458 | 2 | 63374063 | C | T | 0.769 | 0.022 | 0.004 | 2.84E-08 | 0.009 | 0.002 | 4.69E-08 | No | 1 | rs4671458 |
| rs1441102 | 2 | 104479862 | A | T | 0.476 | 0.021 | 0.003 | 2.20E-10 | 0.009 | 0.001 | 1.43E-10 | No | 0.328 | rs2570485 |
| rs62170849 | 2 | 125012926 | T | C | 0.707 | 0.023 | 0.004 | 1.39E-10 | 0.010 | 0.002 | 1.67E-10 | No | 1 | rs62170849 |
| rs6713237 | 2 | 142489456 | T | C | 0.141 | 0.026 | 0.005 | 1.10E-08 | 0.012 | 0.002 | 9.27E-09 | No |  |  |
| rs1320138 | 2 | 144158287 | T | C | 0.437 | 0.018 | 0.003 | 3.09E-08 | 0.008 | 0.001 | 3.51E-08 | No | 1 | rs1320138 |
| rs7564151 | 2 | 161356971 | G | C | 0.616 | 0.021 | 0.003 | 6.70E-10 | 0.009 | 0.001 | 6.52E-10 | No |  |  |
| rs1267079 | 2 | 162033854 | T | C | 0.696 | 0.022 | 0.003 | 1.95E-10 | 0.010 | 0.002 | 1.79E-10 | No | 1 | rs1267079 |
| rs4972656 | 2 | 175240292 | C | T | 0.415 | -0.019 | 0.003 | 3.23E-09 | -0.008 | 0.001 | 4.11E-09 | No | 1 | rs4972656 |
| rs2111593 | 2 | 208046998 | C | T | 0.645 | -0.023 | 0.003 | 3.46E-12 | -0.010 | 0.001 | 4.86E-12 | No | 0.827 | rs55772859 |
| rs55897719 | 2 | 212590841 | C | A | 0.682 | -0.019 | 0.003 | 3.14E-08 | -0.008 | 0.002 | 3.12E-08 | No |  |  |
| rs7629352 | 3 | 16848835 | A | G | 0.695 | -0.022 | 0.003 | 6.80E-11 | -0.010 | 0.002 | 7.52E-11 | No | 0.744 | rs6800583 |
| rs56029819 | 3 | 43478295 | T | C | 0.848 | -0.027 | 0.005 | 1.28E-09 | -0.012 | 0.002 | 1.47E-09 | No | 0.970 | rs77463213 |
| rs1105023 | 3 | 44222351 | T | G | 0.494 | 0.020 | 0.003 | 9.45E-10 | 0.008 | 0.001 | 1.43E-09 | No | 0.071 | rs13058913 |
| rs6765468 | 3 | 44609626 | G | A | 0.670 | -0.024 | 0.003 | 5.20E-13 | -0.011 | 0.001 | 9.28E-13 | No | 0.867 | rs13058913 |
| rs7617480 | 3 | 49210732 | A | C | 0.209 | 0.032 | 0.004 | 2.11E-17 | 0.014 | 0.002 | 4.52E-17 | No | 1 | rs7617480 |
| rs7621797 | 3 | 49798864 | G | A | 0.844 | -0.034 | 0.004 | 8.67E-16 | -0.015 | 0.002 | 1.08E-15 | No | 0.514 | rs7617480 |
| rs3774364 | 3 | 52863605 | G | A | 0.617 | 0.018 | 0.003 | 1.80E-08 | 0.008 | 0.001 | 1.41E-08 | No | 0.381 | rs1010553 |
| rs66511648 | 3 | 117515519 | T | C | 0.738 | -0.023 | 0.004 | 1.25E-10 | -0.010 | 0.002 | 1.53E-10 | No | 0.941 | rs6783233 |
| rs13066362 | 3 | 158009477 | A | G | 0.556 | -0.019 | 0.003 | 1.69E-09 | -0.008 | 0.001 | 2.50E-09 | No |  |  |
| rs536445 | 3 | 173120103 | C | T | 0.473 | -0.018 | 0.003 | 8.66E-09 | -0.008 | 0.001 | 8.11E-09 | No | 1 | rs536445 |
| rs362280 | 4 | 3260057 | A | G | 0.528 | 0.018 | 0.003 | 2.50E-08 | 0.008 | 0.001 | 1.91E-08 | No | 0.268 | rs2298969 |
| rs114851235 | 4 | 59833409 | A | G | 0.941 | -0.048 | 0.008 | 2.70E-10 | -0.018 | 0.003 | 4.83E-10 | No | 1 | rs114851235 |
| rs7441547 | 4 | 59838120 | A | G | 0.511 | 0.019 | 0.003 | 6.11E-10 | 0.009 | 0.001 | 6.50E-10 | No |  |  |
| rs34207047 | 4 | 80199323 | C | A | 0.487 | 0.020 | 0.003 | 1.86E-10 | 0.009 | 0.001 | 3.05E-10 | No | 1 | rs34207047 |
| rs6831759 | 4 | 90989319 | T | C | 0.340 | -0.019 | 0.003 | 1.72E-08 | -0.008 | 0.001 | 1.73E-08 | No | 0.818 | rs6813752 |
| rs78689878 | 4 | 123096690 | G | A | 0.969 | 0.055 | 0.010 | 1.05E-08 | 0.023 | 0.004 | 1.21E-08 | No | 0.576 | rs45510091 |
| rs34973539 | 4 | 140940476 | T | A | 0.475 | 0.020 | 0.003 | 2.66E-10 | 0.009 | 0.001 | 3.18E-10 | No | 0.945 | rs6536630 |
| rs1128956 | 4 | 183724005 | T | G | 0.842 | 0.025 | 0.004 | 3.20E-09 | 0.011 | 0.002 | 2.82E-09 | No |  |  |
| rs993885 | 5 | 7379003 | G | A | 0.662 | -0.019 | 0.003 | 2.97E-08 | -0.008 | 0.001 | 3.66E-08 | No | 1 | rs2220599 |
| rs1435977 | 5 | 20150939 | T | C | 0.484 | 0.018 | 0.003 | 1.16E-08 | 0.008 | 0.001 | 1.06E-08 | No | 0.437 | rs7700453 |
| rs10057876 | 5 | 61487083 | C | T | 0.484 | -0.023 | 0.003 | 3.67E-13 | -0.010 | 0.001 | 6.13E-13 | No | 0.980 | rs12521969 |
| rs1644039 | 5 | 87909877 | C | T | 0.525 | 0.018 | 0.003 | 2.73E-08 | 0.008 | 0.001 | 4.43E-08 | No | 0.207 | rs17548339 |
| rs10514370 | 5 | 92435481 | G | T | 0.609 | 0.020 | 0.003 | 8.81E-10 | 0.009 | 0.001 | 1.15E-09 | No | 0.983 | rs4489042 |
| rs30266 | 5 | 103972357 | G | A | 0.687 | -0.037 | 0.004 | 2.78E-18 | -0.013 | 0.002 | 3.83E-18 | No |  |  |
| rs62379847 | 5 | 120109119 | A | C | 0.649 | -0.021 | 0.003 | 1.62E-09 | -0.009 | 0.001 | 2.18E-09 | No | 1 | rs62379847 |
| rs2408225 | 5 | 124262051 | T | C | 0.551 | 0.018 | 0.003 | 4.53E-08 | 0.008 | 0.001 | 4.44E-08 | No | 1 | rs2408225 |
| rs1993739 | 5 | 153215007 | C | T | 0.816 | 0.024 | 0.004 | 2.21E-08 | 0.010 | 0.002 | 2.20E-08 | No | 1 | rs2964003 |
| rs12716387 | 5 | 161319595 | T | C | 0.364 | 0.019 | 0.003 | 9.28E-09 | 0.008 | 0.001 | 7.32E-09 | No | 1 | rs2279020 |
| rs11135349 | 5 | 164523472 | A | C | 0.442 | -0.022 | 0.003 | 3.36E-12 | -0.010 | 0.001 | 7.77E-12 | No |  |  |
| rs17068508 | 5 | 166887538 | C | G | 0.957 | -0.051 | 0.008 | 1.99E-09 | -0.021 | 0.003 | 2.65E-09 | No | 0.055 | rs4869058 |
| rs2219498 | 5 | 167059459 | C | T | 0.534 | -0.018 | 0.003 | 1.06E-08 | -0.008 | 0.001 | 2.17E-08 | No |  |  |
| rs13198474 | 6 | 25874423 | G | A | 0.930 | 0.038 | 0.006 | 1.11E-09 | 0.016 | 0.003 | 1.92E-09 | No | 0.548 | rs35883476 |
| rs1624440 | 6 | 26433329 | G | A | 0.472 | 0.020 | 0.003 | 6.99E-10 | 0.008 | 0.001 | 1.88E-09 | No |  |  |
| rs72843784 | 6 | 26498758 | G | T | 0.921 | 0.047 | 0.006 | 2.11E-15 | 0.020 | 0.003 | 1.22E-14 | No | 0.752 | rs35883476 |
| rs67092078 | 6 | 27054772 | G | A | 0.920 | 0.048 | 0.006 | 7.18E-16 | 0.021 | 0.003 | 1.65E-15 | No | 0.767 | rs35883476 |
| rs17737641 | 6 | 27174557 | A | G | 0.972 | -0.061 | 0.009 | 2.54E-11 | -0.028 | 0.004 | 5.52E-11 | No | 0.063 | rs115938232 |
| rs7772160 | 6 | 27412386 | C | T | 0.482 | -0.022 | 0.003 | 7.73E-12 | -0.009 | 0.001 | 1.43E-11 | No | 0.086 | rs35883476 |
| rs115724600 | 6 | 27683371 | G | T | 0.976 | -0.054 | 0.010 | 1.65E-08 | -0.025 | 0.005 | 3.30E-08 | No | 0.136 | rs115938232 |
| rs17693963 | 6 | 27710165 | A | C | 0.908 | 0.050 | 0.006 | 8.12E-19 | 0.021 | 0.002 | 3.26E-18 | No | 0.682 | rs35883476 |
| rs13208096 | 6 | 28225311 | C | G | 0.917 | 0.052 | 0.006 | 5.70E-19 | 0.022 | 0.003 | 2.73E-18 | No | 0.969 | rs35883476 |
| rs140427260 | 6 | 28415601 | T | C | 0.959 | -0.045 | 0.008 | 9.98E-09 | -0.020 | 0.004 | 2.22E-08 | No | 0.242 | rs115938232 |
| rs9257184 | 6 | 28756088 | C | T | 0.915 | 0.056 | 0.006 | 8.42E-22 | 0.024 | 0.003 | 6.55E-21 | No | 0.926 | rs35883476 |
| rs9257405 | 6 | 28922437 | T | A | 0.608 | -0.019 | 0.003 | 4.84E-09 | -0.008 | 0.001 | 4.04E-09 | No |  |  |
| rs149142151 | 6 | 28941134 | C | T | 0.965 | -0.054 | 0.008 | 2.14E-11 | -0.025 | 0.004 | 5.46E-11 | No | 0.340 | rs115938232 |
| rs9257809 | 6 | 29356331 | A | G | 0.910 | 0.057 | 0.006 | 7.99E-23 | 0.024 | 0.002 | 2.32E-22 | No | 0.740 | rs35883476 |
| rs115938232 | 6 | 29725013 | A | G | 0.951 | -0.052 | 0.007 | 7.62E-14 | -0.024 | 0.003 | 1.31E-13 | No | 1 | rs115938232 |
| rs3094146 | 6 | 29970960 | G | C | 0.903 | 0.052 | 0.006 | 1.49E-19 | 0.021 | 0.002 | 4.14E-19 | No | 0.632 | rs35883476 |
| rs3130396 | 6 | 30223490 | T | C | 0.574 | 0.019 | 0.003 | 2.52E-09 | 0.008 | 0.001 | 4.23E-09 | No | 0.079 | rs35883476 |
| rs28780098 | 6 | 30303368 | G | T | 0.947 | -0.043 | 0.007 | 1.15E-10 | -0.020 | 0.003 | 1.65E-10 | No | 0.633 | rs115938232 |
| rs3131781 | 6 | 30937732 | A | G | 0.884 | 0.037 | 0.005 | 6.11E-12 | 0.015 | 0.002 | 1.62E-11 | No | 0.485 | rs35883476 |
| rs13207315 | 6 | 31241127 | T | C | 0.881 | -0.028 | 0.005 | 3.27E-09 | -0.013 | 0.002 | 5.64E-09 | No |  |  |
| rs2736155 | 6 | 31605199 | G | C | 0.547 | -0.018 | 0.003 | 4.60E-08 | -0.008 | 0.001 | 4.31E-08 | No |  |  |
| rs9368699 | 6 | 31802541 | T | C | 0.962 | -0.045 | 0.008 | 1.94E-08 | -0.020 | 0.004 | 2.31E-08 | No | 0.064 | rs115938232 |
| rs537362 | 6 | 50650700 | G | C | 0.082 | 0.031 | 0.006 | 1.70E-08 | 0.014 | 0.003 | 1.51E-08 | No | 1 | rs537362 |
| rs72904286 | 6 | 51414922 | C | T | 0.889 | 0.030 | 0.005 | 9.11E-09 | 0.013 | 0.002 | 1.29E-08 | No | 0.971 | rs17752199 |
| rs632024 | 6 | 64630933 | T | G | 0.294 | -0.020 | 0.003 | 7.05E-09 | -0.009 | 0.002 | 5.99E-09 | No | 1 | rs632024 |
| rs2214123 | 6 | 67000001 | A | G | 0.360 | 0.022 | 0.003 | 5.81E-11 | 0.009 | 0.001 | 1.33E-10 | No |  |  |
| rs9322279 | 6 | 101347943 | G | T | 0.531 | 0.019 | 0.003 | 7.94E-10 | 0.009 | 0.001 | 5.77E-10 | No | 0.722 | rs12530388 |
| rs12110689 | 6 | 103025230 | A | G | 0.607 | 0.018 | 0.003 | 2.38E-08 | 0.008 | 0.001 | 3.18E-08 | No |  |  |
| rs728017 | 6 | 124292594 | A | G | 0.411 | -0.019 | 0.003 | 8.03E-09 | -0.008 | 0.001 | 5.73E-09 | No | 1 | rs728017 |
| rs2876520 | 6 | 142996618 | C | G | 0.541 | -0.019 | 0.003 | 3.70E-09 | -0.008 | 0.001 | 4.73E-09 | No | 1 | rs2876520 |
| rs9373363 | 6 | 143150043 | A | G | 0.723 | 0.022 | 0.004 | 4.17E-09 | 0.009 | 0.002 | 6.40E-09 | No |  |  |
| rs11756123 | 6 | 152218079 | A | T | 0.359 | 0.025 | 0.003 | 3.10E-14 | 0.011 | 0.001 | 2.86E-14 | No | 1 | rs9479138 |
| rs12190823 | 6 | 163092643 | G | A | 0.770 | -0.021 | 0.004 | 1.98E-08 | -0.009 | 0.002 | 1.71E-08 | No | 1 | rs12190823 |
| rs76149263 | 6 | 165178503 | C | T | 0.718 | -0.022 | 0.004 | 1.32E-09 | -0.010 | 0.002 | 1.01E-09 | No | 0.270 | rs2322702 |
| rs868754 | 7 | 2033780 | G | C | 0.784 | 0.034 | 0.004 | 4.18E-17 | 0.014 | 0.002 | 4.09E-17 | No | 0.703 | rs10268609 |
| rs1637736 | 7 | 2246454 | T | C | 0.379 | -0.019 | 0.003 | 8.26E-09 | -0.008 | 0.001 | 1.22E-08 | No |  |  |
| rs957360 | 7 | 3660918 | C | G | 0.699 | 0.020 | 0.004 | 7.66E-09 | 0.009 | 0.002 | 7.07E-09 | No | 1 | rs957360 |
| rs7791726 | 7 | 12283329 | G | C | 0.602 | -0.023 | 0.003 | 3.02E-12 | -0.010 | 0.001 | 1.01E-12 | No | 1 | rs6460905 |
| rs2247523 | 7 | 82454404 | C | G | 0.526 | -0.019 | 0.003 | 5.24E-09 | -0.008 | 0.001 | 8.75E-09 | No | 0.547 | rs12707530 |
| rs2894699 | 7 | 114059156 | C | T | 0.559 | 0.022 | 0.003 | 6.65E-12 | 0.010 | 0.001 | 5.79E-12 | No | 0.973 | rs8180817 |
| rs1548461 | 7 | 117567576 | T | C | 0.471 | -0.020 | 0.003 | 4.84E-10 | -0.009 | 0.001 | 6.73E-10 | No | 1 | rs1548461 |
| rs28897173 | 8 | 14218081 | T | C | 0.722 | 0.021 | 0.004 | 2.71E-09 | 0.009 | 0.002 | 3.52E-09 | No | 0.963 | rs34269011 |
| rs2256035 | 8 | 14628926 | A | G | 0.388 | -0.018 | 0.003 | 1.84E-08 | -0.008 | 0.001 | 3.37E-08 | No |  |  |
| rs10098006 | 8 | 65572919 | C | G | 0.144 | -0.026 | 0.004 | 3.79E-09 | -0.012 | 0.002 | 3.56E-09 | No | 1 | rs7837935 |
| rs4628229 | 8 | 129882550 | T | C | 0.308 | -0.020 | 0.004 | 1.49E-08 | -0.009 | 0.002 | 1.33E-08 | No | 0.986 | rs6470670 |
| rs72694255 | 9 | 11130263 | G | C | 0.896 | 0.034 | 0.006 | 5.85E-10 | 0.014 | 0.002 | 8.52E-10 | No | 0.081 | rs1931388 |
| rs10809385 | 9 | 11208995 | C | A | 0.592 | 0.026 | 0.003 | 8.03E-16 | 0.012 | 0.001 | 4.92E-16 | No | 1 | rs1931388 |
| rs62555663 | 9 | 11724099 | C | A | 0.799 | 0.026 | 0.004 | 2.86E-11 | 0.012 | 0.002 | 4.63E-11 | No | 0.134 | rs1931388 |
| rs3793577 | 9 | 23737627 | A | G | 0.473 | -0.022 | 0.003 | 5.04E-12 | -0.010 | 0.001 | 9.93E-12 | No | 1 | rs3793577 |
| rs13296641 | 9 | 31160243 | G | A | 0.684 | 0.020 | 0.003 | 2.30E-09 | 0.009 | 0.002 | 2.99E-09 | No | 1 | rs13296641 |
| rs1329572 | 9 | 37001471 | T | A | 0.583 | -0.024 | 0.003 | 2.71E-13 | -0.010 | 0.001 | 2.48E-13 | No | 1 | rs1329572 |
| rs62535714 | 9 | 37182655 | G | A | 0.811 | -0.032 | 0.004 | 1.40E-14 | -0.014 | 0.002 | 2.06E-14 | No | 0.075 | rs1329572 |
| rs6479487 | 9 | 96237373 | T | G | 0.839 | -0.030 | 0.004 | 2.60E-12 | -0.013 | 0.002 | 2.67E-12 | No | 0.932 | rs7023933 |
| rs10821163 | 9 | 96343060 | G | C | 0.645 | 0.022 | 0.003 | 1.09E-10 | 0.009 | 0.001 | 2.17E-10 | No | 0.086 | rs7023933 |
| rs182923402 | 9 | 98299677 | T | A | 0.911 | -0.036 | 0.006 | 8.17E-10 | -0.015 | 0.002 | 9.50E-10 | No | 1 | rs182923402 |
| rs1888286 | 9 | 119726051 | T | C | 0.707 | 0.022 | 0.004 | 5.78E-10 | 0.009 | 0.002 | 8.68E-10 | No | 0.943 | rs2418449 |
| rs7025421 | 9 | 122666288 | C | T | 0.392 | -0.021 | 0.003 | 3.29E-11 | -0.010 | 0.001 | 2.37E-11 | No | 0.963 | rs12553064 |
| rs73581580 | 9 | 140251458 | G | A | 0.850 | -0.031 | 0.005 | 4.83E-10 | -0.012 | 0.002 | 3.80E-10 | No | 0.801 | rs112535944 |
| rs4620605 | 10 | 68532027 | C | T | 0.196 | 0.022 | 0.004 | 2.06E-08 | 0.010 | 0.002 | 1.55E-08 | No | 0.987 | rs4515864 |
| rs3088142 | 10 | 76854564 | C | T | 0.570 | -0.019 | 0.003 | 5.98E-09 | -0.008 | 0.001 | 6.43E-09 | No | 1 | rs3088142 |
| rs1909696 | 10 | 77582203 | G | T | 0.323 | -0.019 | 0.003 | 2.54E-08 | -0.008 | 0.001 | 3.97E-08 | No | 1 | rs1909696 |
| rs11191658 | 10 | 105096021 | A | G | 0.678 | 0.020 | 0.004 | 2.30E-08 | 0.008 | 0.001 | 4.10E-08 | No |  |  |
| rs2151971 | 10 | 106445916 | C | T | 0.501 | 0.019 | 0.003 | 7.18E-10 | 0.009 | 0.001 | 5.39E-10 | No | 0.109 | rs1021363 |
| rs11192191 | 10 | 106554691 | T | G | 0.781 | -0.022 | 0.004 | 7.82E-09 | -0.010 | 0.002 | 7.81E-09 | No | 0.081 | rs1021363 |
| rs1021363 | 10 | 106610839 | A | G | 0.329 | 0.030 | 0.003 | 4.93E-19 | 0.013 | 0.001 | 3.49E-18 | No | 1 | rs1021363 |
| rs10400180 | 10 | 106755196 | T | G | 0.773 | 0.027 | 0.004 | 3.53E-12 | 0.012 | 0.002 | 3.93E-12 | No | 0.501 | rs17186548 |
| rs111442587 | 10 | 114984089 | C | G | 0.975 | -0.059 | 0.011 | 2.33E-08 | -0.025 | 0.004 | 2.14E-08 | No | 1 | rs111442587 |
| rs2585811 | 11 | 28618411 | G | A | 0.626 | 0.026 | 0.003 | 2.35E-15 | 0.011 | 0.001 | 2.48E-15 | No | 0.876 | rs11030380 |
| rs927456 | 11 | 29770417 | T | C | 0.675 | -0.020 | 0.003 | 9.42E-09 | -0.009 | 0.001 | 7.02E-09 | No | 0.390 | rs324300 |
| rs145678014 | 11 | 32927778 | G | T | 0.962 | 0.050 | 0.008 | 5.58E-10 | 0.022 | 0.004 | 8.13E-10 | No | 0.608 | rs80067584 |
| rs2509805 | 11 | 57650796 | T | C | 0.334 | 0.023 | 0.003 | 1.61E-11 | 0.010 | 0.001 | 1.58E-11 | No | 1 | rs2509805 |
| rs174546 | 11 | 61569830 | C | T | 0.637 | -0.019 | 0.003 | 1.36E-08 | -0.008 | 0.001 | 2.41E-08 | No | 0.933 | rs102275 |
| rs61903412 | 11 | 88890451 | G | A | 0.490 | 0.021 | 0.003 | 1.60E-11 | 0.009 | 0.001 | 3.09E-11 | No | 0.456 | rs4753209 |
| rs1940729 | 11 | 112852192 | T | C | 0.533 | -0.021 | 0.003 | 1.86E-10 | -0.009 | 0.001 | 9.96E-11 | No | 0.865 | rs4937872 |
| rs11214595 | 11 | 113259658 | G | T | 0.481 | 0.024 | 0.003 | 6.72E-14 | 0.010 | 0.001 | 9.84E-14 | No | 0.069 | rs61902811 |
| rs61902811 | 11 | 113370758 | G | A | 0.684 | 0.028 | 0.003 | 1.54E-17 | 0.013 | 0.002 | 9.96E-18 | No | 1 | rs61902811 |
| rs73004016 | 11 | 113444727 | T | C | 0.889 | 0.035 | 0.005 | 9.42E-11 | 0.015 | 0.002 | 7.46E-11 | No |  |  |
| rs7940164 | 11 | 113451765 | G | T | 0.336 | 0.022 | 0.003 | 9.42E-11 | 0.010 | 0.001 | 1.29E-10 | No | 0.134 | rs7130926 |
| rs612823 | 11 | 133834104 | T | C | 0.579 | 0.023 | 0.003 | 9.20E-12 | 0.010 | 0.001 | 8.97E-12 | No | 1 | rs612823 |
| rs11169666 | 12 | 39211300 | T | C | 0.579 | 0.018 | 0.003 | 3.60E-08 | 0.008 | 0.001 | 3.25E-08 | No |  |  |
| rs11612312 | 12 | 52349088 | T | C | 0.800 | -0.025 | 0.004 | 3.04E-10 | -0.011 | 0.002 | 1.09E-09 | No | 1 | rs11612312 |
| rs11172247 | 12 | 57946510 | G | C | 0.409 | -0.018 | 0.003 | 4.29E-08 | -0.008 | 0.001 | 4.70E-08 | No | 0.253 | rs73123350 |
| rs2292996 | 12 | 103556972 | T | C | 0.462 | -0.018 | 0.003 | 3.77E-08 | -0.008 | 0.001 | 3.65E-08 | No | 1 | rs2292996 |
| rs521048 | 12 | 118830958 | A | G | 0.268 | -0.023 | 0.004 | 2.12E-09 | -0.010 | 0.002 | 1.65E-09 | No | 1 | rs521048 |
| rs4457791 | 12 | 118877949 | T | G | 0.834 | -0.024 | 0.004 | 5.62E-08 | -0.010 | 0.002 | 4.12E-08 | No | 0.083 | rs521048 |
| rs58291418 | 12 | 121264010 | C | T | 0.528 | -0.019 | 0.003 | 8.13E-09 | -0.008 | 0.001 | 9.64E-09 | No | 0.984 | rs3213572 |
| rs9530139 | 13 | 31847324 | C | T | 0.779 | 0.024 | 0.004 | 1.30E-09 | 0.010 | 0.002 | 1.31E-09 | No | 0.860 | rs7334101 |
| rs9527064 | 13 | 53897024 | G | A | 0.672 | -0.021 | 0.003 | 1.00E-09 | -0.009 | 0.001 | 1.65E-09 | No | 0.828 | rs9536383 |
| rs508502 | 13 | 80921519 | C | T | 0.689 | 0.023 | 0.004 | 8.69E-11 | 0.010 | 0.002 | 1.09E-10 | No | 1 | rs508502 |
| rs9561331 | 13 | 94017476 | G | A | 0.876 | -0.027 | 0.005 | 1.06E-08 | -0.012 | 0.002 | 1.01E-08 | No | 1 | rs9561328 |
| rs510339 | 13 | 97286612 | G | A | 0.164 | -0.024 | 0.004 | 1.39E-08 | -0.011 | 0.002 | 1.47E-08 | No | 0.484 | rs7317645 |
| rs4772087 | 13 | 99115041 | C | T | 0.615 | -0.019 | 0.003 | 1.29E-08 | -0.008 | 0.001 | 1.94E-08 | No | 1 | rs4772087 |
| rs2038257 | 14 | 33311424 | T | A | 0.285 | -0.019 | 0.004 | 4.27E-08 | -0.008 | 0.002 | 4.93E-08 | No |  |  |
| rs61990289 | 14 | 42076434 | C | T | 0.494 | 0.024 | 0.003 | 1.07E-14 | 0.011 | 0.001 | 7.18E-15 | No | 0.945 | rs11157241 |
| rs2933195 | 14 | 47293348 | A | G | 0.553 | -0.019 | 0.003 | 4.25E-09 | -0.008 | 0.001 | 5.23E-09 | No |  |  |
| rs2274793 | 14 | 57283968 | C | T | 0.666 | 0.020 | 0.003 | 2.63E-09 | 0.009 | 0.001 | 3.57E-09 | No | 1 | rs2274794 |
| rs11621186 | 14 | 75120628 | C | T | 0.504 | -0.022 | 0.003 | 6.07E-12 | -0.010 | 0.001 | 8.44E-12 | No | 1 | rs11621186 |
| rs2896460 | 14 | 103306106 | A | T | 0.752 | -0.023 | 0.004 | 1.13E-09 | -0.010 | 0.002 | 1.75E-09 | No | 0.947 | rs9671376 |
| rs10149470 | 14 | 104017953 | A | G | 0.485 | -0.023 | 0.003 | 3.02E-12 | -0.010 | 0.001 | 1.08E-11 | No | 1 | rs10149470 |
| rs111280186 | 14 | 104357988 | C | G | 0.877 | -0.027 | 0.005 | 2.50E-08 | -0.012 | 0.002 | 2.42E-08 | No | 0.070 | rs10149470 |
| rs56059718 | 15 | 38836777 | C | A | 0.806 | -0.022 | 0.004 | 2.24E-08 | -0.010 | 0.002 | 3.10E-08 | No | 1 | rs56059718 |
| rs12901436 | 15 | 47683095 | C | G | 0.784 | -0.024 | 0.004 | 1.04E-09 | -0.010 | 0.002 | 1.02E-09 | No | 1 | rs34488670 |
| rs74927570 | 15 | 63802297 | T | G | 0.805 | -0.025 | 0.004 | 7.94E-10 | -0.011 | 0.002 | 6.65E-10 | No | 0.686 | rs79714693 |
| rs4776729 | 15 | 66399197 | A | G | 0.876 | -0.028 | 0.005 | 5.69E-09 | -0.012 | 0.002 | 1.06E-08 | No | 1 | rs4776729 |
| rs4404022 | 15 | 74010430 | A | T | 0.600 | -0.019 | 0.003 | 6.92E-09 | -0.008 | 0.001 | 5.50E-09 | No |  |  |
| rs72743363 | 15 | 74104811 | G | A | 0.863 | 0.033 | 0.005 | 3.59E-12 | 0.014 | 0.002 | 2.99E-12 | No | 0.651 | rs8030745 |
| rs8035777 | 15 | 88943807 | A | G | 0.778 | 0.022 | 0.004 | 7.09E-09 | 0.010 | 0.002 | 6.27E-09 | No | 0.683 | rs11855600 |
| rs4702 | 15 | 91426560 | G | A | 0.452 | 0.022 | 0.003 | 7.64E-12 | 0.009 | 0.001 | 2.16E-11 | No | 1 | rs4702 |
| rs12935276 | 16 | 13038723 | T | G | 0.708 | -0.022 | 0.004 | 1.63E-09 | -0.009 | 0.002 | 1.84E-09 | No | 0.911 | rs7200826 |
| rs12919291 | 16 | 13800430 | G | C | 0.821 | -0.023 | 0.004 | 2.04E-08 | -0.010 | 0.002 | 2.29E-08 | No |  |  |
| rs2369818 | 16 | 21614009 | C | T | 0.581 | -0.022 | 0.003 | 1.94E-11 | -0.009 | 0.001 | 2.70E-11 | No | 1 | rs2369818 |
| rs55730016 | 16 | 49495173 | C | T | 0.647 | -0.019 | 0.004 | 3.77E-08 | -0.008 | 0.001 | 3.05E-08 | No | 0.576 | rs4785307 |
| rs2111568 | 16 | 49498861 | G | C | 0.721 | -0.022 | 0.004 | 1.75E-08 | -0.009 | 0.002 | 3.28E-08 | No | 0.085 | rs4785307 |
| rs1862743 | 16 | 60743834 | C | A | 0.511 | 0.020 | 0.003 | 7.43E-10 | 0.009 | 0.001 | 5.35E-10 | No |  |  |
| rs407502 | 17 | 31539581 | G | A | 0.283 | -0.021 | 0.004 | 1.27E-08 | -0.009 | 0.002 | 1.89E-08 | No | 0.887 | rs412891 |
| rs9915591 | 17 | 65826090 | C | G | 0.785 | -0.024 | 0.004 | 1.28E-09 | -0.010 | 0.002 | 1.24E-09 | No | 0.757 | rs60856912 |
| rs11081827 | 18 | 31390228 | C | A | 0.517 | -0.020 | 0.003 | 3.01E-10 | -0.009 | 0.001 | 2.56E-10 | No | 0.859 | rs10460051 |
| rs12967855 | 18 | 35138245 | A | G | 0.315 | 0.028 | 0.003 | 1.64E-16 | 0.012 | 0.002 | 1.66E-16 | No | 1 | rs12967855 |
| rs4267411 | 18 | 39305256 | C | T | 0.827 | 0.026 | 0.004 | 4.69E-10 | 0.012 | 0.002 | 5.22E-10 | No | 1 | rs4267411 |
| rs7232516 | 18 | 50389194 | G | A | 0.864 | 0.027 | 0.005 | 1.39E-08 | 0.011 | 0.002 | 2.28E-08 | No | 0.064 | rs7230285 |
| rs4632195 | 18 | 50746748 | C | T | 0.497 | -0.027 | 0.003 | 6.85E-18 | -0.012 | 0.001 | 3.04E-17 | No | 0.942 | rs7230285 |
| rs72925321 | 18 | 50807202 | G | A | 0.934 | 0.046 | 0.007 | 1.67E-11 | 0.019 | 0.003 | 1.34E-11 | No |  |  |
| rs8093506 | 18 | 52474170 | A | G | 0.733 | 0.028 | 0.004 | 1.64E-13 | 0.012 | 0.002 | 1.54E-13 | No | 0.700 | rs1819881 |
| rs4128242 | 18 | 52747689 | T | C | 0.581 | 0.024 | 0.003 | 5.44E-14 | 0.011 | 0.001 | 5.03E-14 | No | 1 | rs4131791 |
| rs1942569 | 18 | 52846725 | T | G | 0.518 | -0.019 | 0.003 | 9.74E-10 | -0.008 | 0.001 | 2.09E-09 | No | 0.087 | rs12967143 |
| rs12967143 | 18 | 53099012 | G | C | 0.287 | 0.030 | 0.004 | 7.52E-18 | 0.013 | 0.002 | 2.85E-17 | No | 1 | rs12967143 |
| rs896686 | 18 | 53123031 | T | G | 0.819 | 0.027 | 0.004 | 2.27E-10 | 0.011 | 0.002 | 3.78E-10 | No | 0.077 | rs12967143 |
| rs1452787 | 18 | 53207207 | A | G | 0.714 | -0.024 | 0.004 | 4.69E-12 | -0.011 | 0.002 | 9.60E-12 | No |  |  |
| rs7241572 | 18 | 77580712 | G | A | 0.794 | -0.029 | 0.005 | 5.39E-10 | -0.011 | 0.002 | 7.82E-10 | No | 1 | rs7241572 |
| rs148108087 | 19 | 51781455 | G | A | 0.937 | 0.041 | 0.007 | 1.90E-08 | 0.016 | 0.003 | 2.45E-08 | No |  |  |
| rs6131010 | 20 | 44724305 | A | G | 0.268 | 0.027 | 0.004 | 3.01E-14 | 0.012 | 0.002 | 2.63E-14 | No | 1 | rs6131010 |
| rs910187 | 20 | 45841052 | G | A | 0.640 | 0.020 | 0.003 | 4.39E-09 | 0.008 | 0.001 | 8.56E-09 | No | 1 | rs910187 |
| rs13056300 | 22 | 41408754 | T | C | 0.719 | -0.023 | 0.004 | 2.53E-10 | -0.010 | 0.002 | 2.87E-10 | No | 0.770 | rs2273085 |
| rs12160976 | 22 | 46438246 | G | A | 0.668 | 0.022 | 0.004 | 2.71E-09 | 0.009 | 0.001 | 3.99E-09 | No | 0.417 | rs9627391 |
| SNP, single nucleotide polymorphisms; CHR, chromosome; BP, physical position of SNP (base-pairs); A1, effect allele; A2, alternative allele; EAF: effect allele frequency; Beta, effect allele beta coefficient; SE, standard error of the beta coefficient. A lead SNP was considered to be novel if the SNP did not reach genome-wide significance in the original single-trait GWAS or the SNP was independent (*r*^2^ < 0.1) of those previously reported genome-wide significant SNPs for single traits. | | | | | | | | | | | | | | |

| **Supplementary Table 9. Lead SNPs for ischemic stroke from MTAG analysis between depression and ischemic stroke.** | | | | | | | | | | | | | | |
| --- | --- | --- | --- | --- | --- | --- | --- | --- | --- | --- | --- | --- | --- | --- |
| SNP | CHR | BP | A1 | A2 | FREQ | Beta | SE | P | MTAG_Beta | MTAG_SE | MTAG_P | Novel | *r*^2^ | LD_Lead_SNP |
| rs880315 | 1 | 10796866 | T | C | 0.632 | -0.045 | 0.008 | 2.00E-09 | -0.008 | 0.001 | 3.55E-09 | No | 1 | rs880315 |
| rs11587860 | 1 | 156156951 | G | C | 0.645 | -0.049 | 0.007 | 2.60E-11 | 0.008 | 0.001 | 1.42E-10 | No | 0.883 | rs2251636 |
| rs2351524 | 2 | 203880992 | T | C | 0.124 | -0.069 | 0.011 | 3.37E-10 | -0.012 | 0.002 | 9.05E-10 | No | 1 | rs2351524 |
| rs6847935 | 4 | 111696651 | A | T | 0.785 | -0.077 | 0.008 | 7.18E-20 | -0.013 | 0.002 | 1.28E-17 | No | 1 | rs6847935 |
| rs56010410 | 4 | 155502869 | T | C | 0.730 | -0.065 | 0.008 | 9.81E-17 | -0.011 | 0.001 | 2.53E-15 | No | 0.216 | rs6536024 |
| rs3756011 | 4 | 187206249 | C | A | 0.594 | 0.044 | 0.007 | 6.31E-10 | -0.008 | 0.001 | 2.50E-09 | No | 0.971 | rs4444878 |
| rs17148926 | 5 | 121510586 | A | C | 0.824 | 0.061 | 0.010 | 2.98E-10 | 0.009 | 0.002 | 3.71E-08 | No | 1 | rs17148926 |
| rs79318212 | 6 | 1365244 | A | G | 0.885 | -0.086 | 0.012 | 5.40E-14 | -0.013 | 0.002 | 9.26E-12 | No | 1 | rs79318212 |
| rs7808568 | 7 | 12271252 | T | C | 0.599 | -0.033 | 0.007 | 3.87E-06 | -0.007 | 0.001 | 1.47E-08 | Yes |  |  |
| rs42036 | 7 | 92241451 | C | G | 0.752 | 0.056 | 0.008 | 1.52E-11 | 0.009 | 0.001 | 1.02E-10 | No | 0.970 | rs42035 |
| rs1549758 | 7 | 150695726 | T | C | 0.302 | 0.048 | 0.008 | 8.97E-09 | 0.008 | 0.001 | 1.55E-08 | No | 1 | rs1549758 |
| rs944797 | 9 | 22115286 | T | C | 0.510 | -0.048 | 0.007 | 6.51E-12 | -0.008 | 0.001 | 1.35E-11 | No | 0.965 | rs7859362 |
| rs532436 | 9 | 136149830 | G | A | 0.802 | 0.063 | 0.009 | 5.30E-13 | -0.010 | 0.002 | 3.14E-10 | No | 0.829 | rs649129 |
| rs10886430 | 10 | 121010256 | A | G | 0.876 | -0.078 | 0.011 | 1.07E-11 | -0.012 | 0.002 | 3.48E-11 | No | 1 | rs10886430 |
| rs150986675 | 11 | 102777303 | T | C | 0.850 | -0.062 | 0.010 | 3.40E-10 | -0.010 | 0.002 | 1.59E-09 | No | 0.581 | rs72985562 |
| rs10774625 | 12 | 111910219 | A | G | 0.479 | 0.065 | 0.007 | 1.22E-19 | 0.010 | 0.001 | 1.51E-16 | No | 1 | rs10774625 |
| rs17696736 | 12 | 112486818 | A | G | 0.571 | -0.060 | 0.007 | 3.46E-17 | -0.009 | 0.001 | 1.49E-13 | No | 0.782 | rs10774625 |
| rs12445022 | 16 | 87575332 | G | A | 0.671 | 0.050 | 0.008 | 9.14E-11 | -0.008 | 0.001 | 2.86E-09 | No | 1 | rs12445022 |
| rs17677363 | 17 | 45036112 | A | T | 0.862 | -0.053 | 0.010 | 1.61E-07 | -0.010 | 0.002 | 2.50E-08 | Yes |  |  |
| rs60314748 | 19 | 11166016 | T | C | 0.667 | 0.043 | 0.008 | 2.55E-08 | 0.007 | 0.001 | 2.69E-08 | No | 0.160 | rs8106503 |
| rs11905081 | 20 | 33640920 | C | T | 0.894 | -0.072 | 0.012 | 5.62E-10 | 0.013 | 0.002 | 4.32E-10 | No | 0.927 | rs11907011 |
| SNP, single nucleotide polymorphisms; CHR, chromosome; BP, physical position of SNP (base-pairs); A1, effect allele; A2, alternative allele; EAF: effect allele frequency; Beta, effect allele beta coefficient; SE, standard error of the beta coefficient. A lead SNP was considered to be novel if the SNP did not reach genome-wide significance in the original single-trait GWAS or the SNP was independent (*r*^2^ < 0.1) of those previously reported genome-wide significant SNPs for single traits. | | | | | | | | | | | | | | |

| **Supplementary Table 10. Shared genetic variants identified by MTAG analysis between depression and stroke** | | | | | | | | | | | | | |
| --- | --- | --- | --- | --- | --- | --- | --- | --- | --- | --- | --- | --- | --- |
| Locus | Trait-pairs | CHR | Depression | | | | Stroke | | | | *r*^2^ | PPH4 | CPASSOC for rs7808568 |
|  |  |  | Lead SNP | Beta | SE | P | Lead SNP | Beta | SE | P |  |  |  |
| TMEM106B | Depression and stroke | 7 | rs6460906 | -0.010 | 0.001 | 8.51E-13 | rs7808568 | -0.007 | 0.001 | 4.58E-09 | 0.955 | 0.856 | 1.62E-15 |
| TMEM106B | Depression and ischemic stroke | 7 | rs7791726 | -0.010 | 0.001 | 1.01E-12 | rs7808568 | -0.007 | 0.001 | 1.47E-08 | 0.955 | 0.856 | 2.45E-15 |
| SNP, single nucleotide polymorphisms; CHR, chromosome. | | | | | | | | | | | | | |

| **Supplementary Table 11. Shared TWAS significant genes between depression and stroke across 49 GTEx Tissues (version 8).** | | | | | | | | | | |
| --- | --- | --- | --- | --- | --- | --- | --- | --- | --- | --- |
| Tissue | Ensembl Gene ID | Gene | Trait-pairs | CHR | Depression | | | Stroke | | |
|  |  |  |  |  | BEST.GWAS.ID | TWAS.P | PPH4 | BEST.GWAS.ID | TWAS.P | PPH4 |
| Colon_Transverse | ENSG00000106460.18 | TMEM106B | Depression and stroke | 7 | rs5011432 | 6.26E-13 | 0.99 | rs1468804 | 7.99E-05 | 0.77 |
| Heart_Atrial_Appendage | ENSG00000182511.11 | FES | Depression and stroke | 15 | rs4702 | 1.72E-05 | 0.94 | rs1573643 | 3.59E-05 | 0.95 |
| Stomach | ENSG00000182511.11 | FES | Depression and stroke | 15 | rs4702 | 2.14E-08 | 0.95 | rs1573643 | 1.35E-04 | 0.92 |
| Colon_Transverse | ENSG00000106460.18 | TMEM106B | Depression and ischemic stroke | 7 | rs5011432 | 6.26E-13 | 0.99 | rs1468804 | 7.21E-05 | 0.74 |
| Heart_Atrial_Appendage | ENSG00000182511.11 | FES | Depression and ischemic stroke | 15 | rs4702 | 1.72E-05 | 0.94 | rs4932370 | 4.06E-05 | 0.91 |
| Stomach | ENSG00000182511.11 | FES | Depression and ischemic stroke | 15 | rs4702 | 2.14E-08 | 0.95 | rs4932370 | 5.46E-05 | 0.82 |

| **Supplementary Table 12. Data sources, sample sizes, number of instruments and F-statistics.** | | | | | | | | | |
| --- | --- | --- | --- | --- | --- | --- | --- | --- | --- |
| Phenotype | IVs | Sample size | R^2^ (%) | F statistics | Author | Ethnicity | Journal | Year | Websites for full summary statistics |
| Depression | 251 | N_case_=371,184/N_control_=978,703 | 0.79 | 42.82 | Thomas D. Als | European | Nature Medicine | 2024 | <https://ipsych.dk/en/research/downloads/> |
| Stroke | 23 | N_case_=73,652/N_control_=1,234,808 | 0.07 | 39.85 | Aniket Mishra | European | Nature | 2022 | GCST90104539 |
| Ischemic stroke | 27 | N_case_=62,100/N_control_=1,220,157 | 0.08 | 38.02 |  |  |  |  | GCST90104540 |
| IV, instrumental variables. R^2^=2×β^2^×MAF×(1-MAF)/(2×β^2^×MAF×(1-MAF)+2SE(β)^2^×N×MAF×(1-MAF)), where β denotes the SNP effect, MAF denotes minor allele frequency, N denotes the sample size. F=((N-K-1)/K)×(R^2^/(1-R^2^)), where N denotes the sample size, K denotes the number of IVs. | | | | | | | | | |

| **Supplementary Table 13. Mendelian randomization mediation analysis between depression and stroke.** | | | | | | | | | |
| --- | --- | --- | --- | --- | --- | --- | --- | --- | --- |
| Outcome | Mediator | Depression → Mediator | | Mediator → Outcome^*^ | | Indirect effect^†^ | | Direct effect^‡^ | |
|  |  | OR (95%CI) | P-value | OR (95%CI) | P-value | OR (95%CI) | P-value | OR (95%CI) | P-value |
| Stroke | Smoking initiation | 1.20 (1.17-1.24) | 1.05E-40 | 1.33 (1.23-1.44) | 2.60E-12 | 1.06 (1.04-1.07) | 6.20E-10 | 1.02 (0.96-1.08) | 0.53 |
|  | Hypertension | 1.03 (1.02-1.04) | 1.85E-08 | 3.77 (3.32-4.28) | 8.13E-93 | 1.05 (1.03-1.06) | 6.02E-08 | 1.01 (0.95-1.07) | 0.72 |
|  | Type 2 diabetes | 1.26 (1.17-1.34) | 3.10E-11 | 1.10 (1.09-1.12) | 4.22E-50 | 1.02 (1.01-1.03) | 1.41E-09 | 1.04 (0.99-1.08) | 0.12 |
|  | Atrial fibrillation | 1.09 (1.02-1.16) | 1.59E-02 | 1.17 (1.15-1.19) | 4.10E-68 | 1.02 (1.01-1.03) | 1.71E-02 | 1.05 (1.00-1.11) | 0.06 |
| Ischemic stroke | Smoking initiation | 1.20 (1.17-1.24) | 1.05E-40 | 1.34 (1.22-1.46) | 8.86E-11 | 1.06 (1.04-1.07) | 5.85E-09 | 1.00 (0.94-1.07) | 0.95 |
|  | Hypertension | 1.03 (1.02-1.04) | 1.85E-08 | 4.01 (3.48-4.61) | 1.27E-82 | 1.05 (1.03-1.06) | 6.91E-08 | 0.99 (0.93-1.05) | 0.70 |
|  | Type 2 diabetes | 1.26 (1.17-1.34) | 3.10E-11 | 1.12 (1.11-1.14) | 7.95E-57 | 1.02 (1.01-1.03) | 9.51E-10 | 1.02 (0.97-1.07) | 0.37 |
|  | Atrial fibrillation | 1.09 (1.02-1.16) | 1.59E-02 | 1.18 (1.16-1.20) | 2.90E-61 | 1.02 (1.01-1.03) | 1.72E-02 | 1.03 (0.97-1.09) | 0.36 |

^*^The effect of the mediator on the outcome was estimated by multivariable Mendelian randomization analysis adjusting for the exposure.

^†^The indirect effect was estimated by multiplying the effect of the mediator by the effect of the mediator on the outcome.

^‡^The direct effect was estimated by multivariable Mendelian randomization analysis adjusting for the mediator.
